# Synthesis, Characterization and In Vitro Evaluation of Novel 5-Ene-thiazolo[3,2-*b*][1,2,4]triazole-6(5*H*)-ones as Possible Anticancer Agents

**DOI:** 10.3390/molecules26041162

**Published:** 2021-02-22

**Authors:** Serhii Holota, Sergiy Komykhov, Stepan Sysak, Andrzej Gzella, Andriy Cherkas, Roman Lesyk

**Affiliations:** 1 Department of Pharmaceutical, Organic and Bioorganic Chemistry, Danylo Halytsky Lviv National Medical University, Pekarska 69, 79010 Lviv, Ukraine; stepansysak@pm.me; 2 Department of Organic Chemistry and Pharmacy, Lesya Ukrainka Volyn National University, Volya Avenue 13, 43025 Lutsk, Ukraine; 3 State Scientific Institution “Institute for Single Crystals”, National Academy of Sciences of Ukraine, Nauky Ave 60, 61072 Kharkiv, Ukraine; 4 Applied Chemistry Department, Karazin Kharkiv National University, Svobody Sq. 4, 61022 Kharkiv, Ukraine; 5 Department of Organic Chemistry, Poznan University of Medical Sciences, Grunwaldzka 6, 60-780 Poznan, Poland; akgzella@ump.edu.pl; 6 Department of Internal Medicine #1, Danylo Halytsky Lviv National Medical University, Pekarska 69, 79010 Lviv, Ukraine; cherkasandriy@yahoo.com or; 7 Department of Public Health, Dietetics and Lifestyle Disorders, Faculty of Medicine, University of Information Technology and Management in Rzeszow, Sucharskiego 2, 35-225 Rzeszow, Poland

**Keywords:** thiazolo[3,2-*b*][1,2,4]triazole-6(5*H*)-ones, multicomponent reactions, *Z*/*E*-isomers, anticancer activity, SAR

## Abstract

The present paper is devoted to the search for drug-like molecules with anticancer properties using the thiazolo[3,2-*b*][1,2,4]triazole-6-one scaffold. A series of 24 novel thiazolo-[3,2-*b*][1,2,4]triazole-6-ones with 5-aryl(heteryl)idene- and 5-aminomethylidene-moieties has been synthesized employing three-component and three-stage synthetic protocols. A mixture of *Z/E*-isomers was obtained in solution for the synthesized 5-aminomethylidene-thiazolo[3,2-*b*]-[1,2,4]triazole-6-ones. The compounds have been studied for their antitumor activity in the NCI 60 lines screen. Some compounds present excellent anticancer properties at 10 μM. Derivatives **2h** and **2i** were the most active against cancer cell lines without causing toxicity to normal somatic (HEK293) cells. A preliminary SAR study had been performed for the synthesized compounds.

## 1. Introduction

We are currently witnessing a great progress in research and development of new anti-tumor therapeutic agents. Nevertheless, cancer remains the second most frequent cause of death in the world and the problem is far from being solved [1]. Cancer is a systemic disease and both its internal metabolic and genetic aberrations as well as the efficiency of immunologic protection play a role in tumor development and progression [2]. Chronic inflammation, redox imbalance, metabolic dysfunctions and altered glucose metabolism as well as many other endogenous and exogenous factors play roles in disease biology and define outcomes [3,4,5]. This heterogeneity of cancer requires the application of various approaches to prevention and treatment that may include application of specific small molecules interfering with altered metabolic pathways in cancers, modulation of immune response to transformed cells and precision medicine [6,7,8]. Therefore, the search for novel small molecules capable to modulate selectively metabolic processes including redox regulation remains of significant scientific and practical importance.

**Figure 1:**
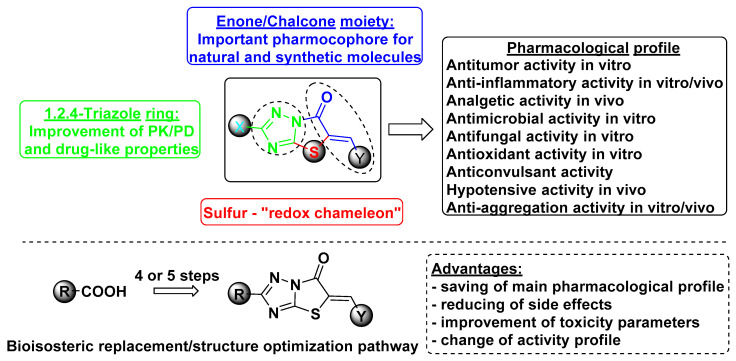
The current state of 5-ene-thiazolo[3,2-*b*][1,2,4]triazole-6(5*H*)-one bearing compounds in medicinal chemistry.

The class of 5-ene-thiazolo[3,2-*b*][1,2,4]triazole-6(5*H*)-ones bicyclic heteroatom-rich compounds [9] containing a 1,2,4-triazole ring and an enone/chalcone system cross-conjugated through a sulfur atom in the molecule has recently attracted the attention of medicinal chemists due to its diverse biological activities. Some 5-ene-thiazolo[3,2-*b*][1,2,4]triazole-6(5*H*)-ones have been studied successfully as potential anti-inflammatory [10,11,12,13,14], analgesic [11,13], antimicrobial [15], antifungal [16], antioxidant [17], anticonvulsant [18,19], antihypertensive [20] and anti-aggregation agents [21] (Figure 1). It is worth mentioning that the structural transformation of the carboxylic group into a 5-ene-thiazolo[3,2-*b*][1,2,4]triazole-6(5*H*)-one moiety has been proposed as a bioisosteric replacement or structure optimization pathway for the synthesis of novel derivatives to keep the main pharmacological profile and to reduce/improve toxicity parameters and activity profiles [10,22,23,24].

**Figure 2:**
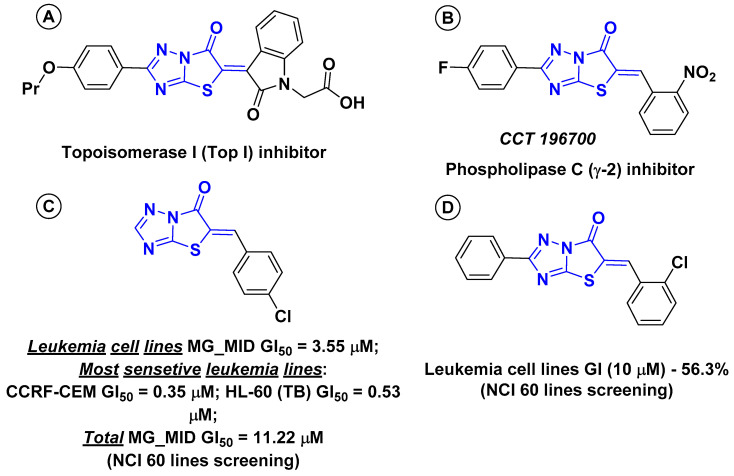
5-Ene-thiazolo[3,2-*b*][1,2,4]triazole-6(5*H*)-ones with anticancer properties.

In addition to the abovementioned pharmacological features, some 5-ene-thiazolo-[3,2-*b*][1,2,4]triazole-6(5*H*)-ones possess promising antitumor properties. A potential non-camptothecin topoisomerase 1 inhibitor (Top1) with the 5-ene-thiazolo[3,2-*b*]-[1,2,4]triazole-6(5*H*)-one scaffold (Figure 2A) had been identified using structure-based virtual screening and in vitro assays [25,26]. The mentioned compound at 10 μM showed superior Top1 inhibitory activity compared with the powerful natural Top1-inhibitor camptothecin. Using a similar screening approach the derivative CCT-196700 (Figure 2B) was identified and reported as a potential phospholipase C-γ2 (PLC-γ2) inhibitor with satisfactory activity parameters at 15 µM in the 3*H*-phosphatidylinositol 4,5-diphosphate biochemical and calcium release cell-based assays [27]. PLC-γ2 is uniquely expressed in hematopoietic cells and considered a plausible target for the treatment of some cancer types [28]. 5-Ene-thiazolo[3,2-*b*][1,2,4]triazole-6(5*H*)-one derivatives (Figure 2C,D) with high impact on leukemia lines in the NCI-60 lines screening program were reported [29,30].

In view of our interest in the search for and study of new low-molecular heterocyclic modulators among thiazole-bearing molecules [31,32,33,34], herein, we report the synthesis, structure characterization and in vitro anticancer activity evaluation of some novel heterocyclic derivatives. This manuscript is intended to draw attention towards the chemistry and pharmacology of the 5-ene-thiazolo[3,2-*b*][1,2,4]triazole-6(5*H*)-one fragment and its further exploration as a prospective source of drug-like molecules.

## 2. Results and Discussion

### 2.1. Chemical Synthesis

**Scheme 1:**
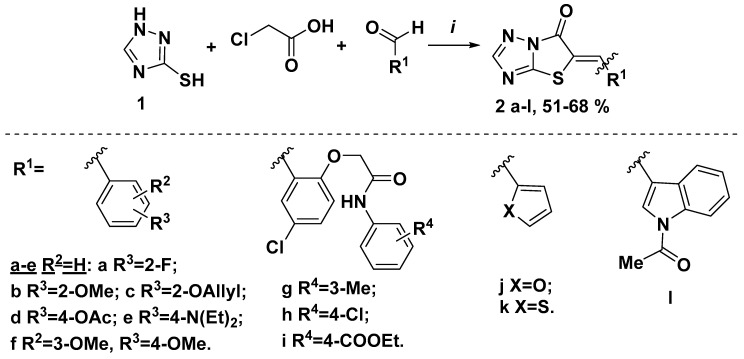
Three-component synthesis of 5-aryl(heteryl)idene-thiazolo[3,2-*b*][1,2,4]triazole-6(5*H*)-ones (**2a–l**). Reagents and conditions: *i*) **1** (10 mmol), ClCH_2_COOH (10 mmol), R^1^-CHO (12 mmol), AcONa (20 mmol), AcOH:Ac_2_O (5 + 5 mL), reflux, 3 h.

According to our synthetic goal and using the retrosynthetic approach, a multicomponent one-pot protocol [29] was employed for the synthesis of 5-aryl(heteryl)idene-derivatives **2a**–**l**. The reported data about the impact of substituents in position C-5 on pharmacological properties of thiazolo[3,2-*b*][1,2,4]triazole-6(5*H*)-ones and data from our study of anticancer activity with structure-activity relationships for this class of heterocyclic compounds [10,11,12,13,14,15,16,17,18,19,20,21,22,23,24,25,26,27,28] were the main reasons for the selection of the substituents R^1^–R^6^. Thereby, by the three-component reaction of **1** with chloroacetic acid and aromatic/heteroaromatic aldehydes in a mixture AcONa + AcOH:Ac_2_O employing convenient heating resulted in compounds **2a**–**l** (Scheme 1) which were purified by recrystallization.

**Scheme 2:**
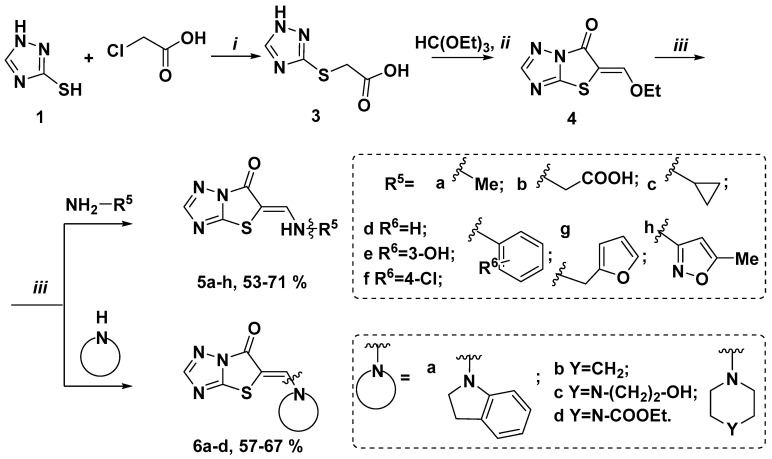
Synthesis of 5-aminomethylidene-thiazolo[3,2-*b*][1,2,4]triazole-6(5*H*)-ones **5a**–**h**, **6a**–**d.** Reagents and conditions: (*i*) **1** (10 mmol), ClCH_2_COOH (10 mmol), AcONa (10 mmol), AcOH 5 mL, reflux, 2 h, 76%; (*ii*) **3** (10 mmol), HC(OEt)_3_, (12 mmol), Ac_2_O (10 mL), reflux, 2 h, 78%; (*iii*) **4** (10 mmol), appropriate amino derivatives (10 mmol), EtOH, reflux, 2 h.

The three-step approach was exploited for the preparation of 5-aminomethylidene-thiazolo[3,2-*b*][1,2,4]triazole-6(5*H*)-ones **5a**–**h**, **6a**–**d** (Scheme 2). Initially the 5-etoxymethylidene derivative **4** was synthesized from **1** based on a published protocol [35]. The aminolysis of **4** by primary or secondary aliphatic/aromatic/heterocyclic amines in an alcoholic medium led to the formation of target derivatives **5a**–**h**, **6a**–**d**. The compounds were obtained in satisfactory yields and were recrystallized for purification (Scheme 2).

### 2.2. Spectral Characterization

The structures of the derivatives **2a**–**l**, **5a**–**h**, **6a**–**d** were confirmed by the ^1^H-NMR, ^13^C-NMR, 2D NMR and LC-MS, (copies of the corresponding spectra are presented in the Supplementary Material).

The ^1^H-NMR spectra of **5a**–**h** contained signals for protons of the double bond at 8.14–8.17 ppm for **5a**–**c** (R^5^ = Alk) and 8.26–8.52 for **5d**–**h** (R^5^ = Ar or Het); for NH proton at 8.78–9.06 ppm for **5a–c** (9.29–10.77 for **5d**–**h**); for the 1,2,4-triazole ring proton at 8.14–8.16 ppm for **5a**–**c** (8.30–8.40 ppm for **5d**–**h**) and signals of appropriate substituents. The ^13^C-NMR spectra contained four respective signals for a bicyclic system and one signal for the ethylene moiety. The assignment in **5b**, **5c** was made based on HSQC and HMBC experiments.

**Scheme 3:**
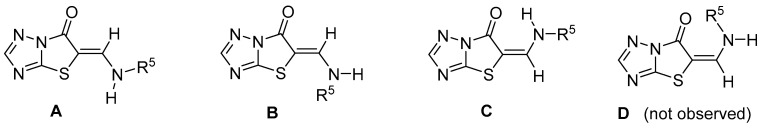
Possible stereoisomeric/rotameric forms of **5a**–**h**.

Compounds **5a**–**h** can exist in four possible stereoisomeric forms A–D (Scheme 3) which significantly vary by stereochemistry of double bond and relative orientation of substituent R^5^.

The ^1^H-NMR spectra of pure samples **5b**,**d**–**h,** beside the signals of the main stereoisomer (I), contained minor signals with relative intensity ~5–15% which were assigned to an additional stereoisomer.

**Figure 3:**
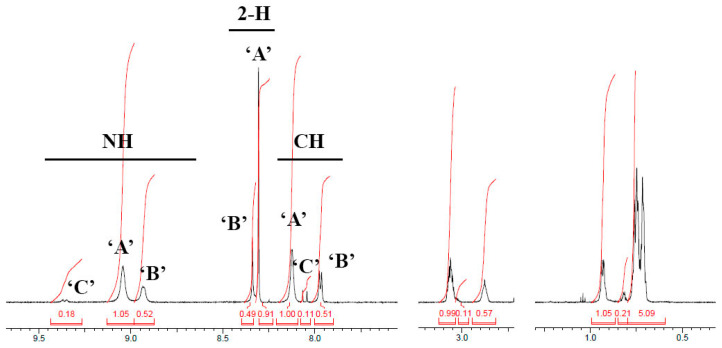
^1^H-NMR spectrum of **5c**. Signals of A, B, C forms of **5c** are shown.

Compound **5c** (R^5^ = cyclopropyl) provided a triple set of signals with relative intensity 62:31:7, presumably for three stereoisomers, **I**, **II** and **III** respectively (Figure 3). The coupling constant *^3^J* between ethylene and NH protons (13.9 Hz) in **III** isomer indicates the *trans*-orientation of these protons which corresponds to either **A** or **C** isomer (Scheme 3). The assignment between **A** and **C** isomers can be performed based on chemical shifts of NH protons. The chemical shift of NH proton in **III** isomer is shifted downfields by 0.30 ppm as compared to the **I** isomer which can be explained by intramolecular interactions which are realized in the **C** isomer (Scheme 3). Therefore, isomers **I** (62%), **II** (31%) and **III** (7%) probably correspond to structures **A**, **B**, and **C**, respectively.

**Figure 4:**
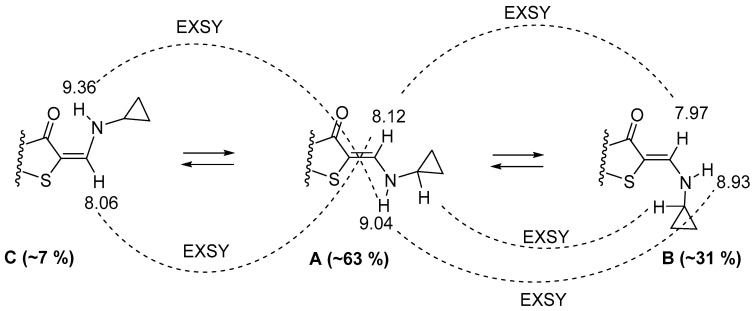
Interpretation of exchange cross-peaks between **A**, **B** and **C** isomers of **5c**.

The NOESY spectrum of **5c** (Figure 4, Supplementary Figure S1) contained EXSY peaks for ethylene, NH and aliphatic protons of **I**, **II** and **III** isomers which indicates the co-existence of those forms of **5c**.

**Scheme 4:**
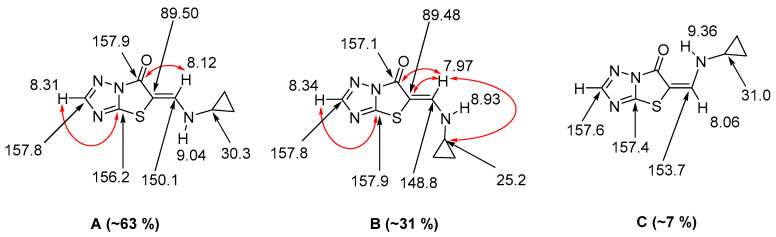
^1^H and ^13^C-NMR chemical shifts of (**A**–**C**) isomers of **5c** with key interactions in the relevant HMBC spectra.

In the ^13^C-NMR spectrum of **5c** signals of **A** and **B** form and some signals of **C** form could be identified (Scheme 4).

The similar pattern of triplicate signals in the ^1^H-NMR spectrum was observed for **5a** (R^5^ = CH_3_) and showed the presence of three stereoisomeric/rotameric forms **A**, **B**, **C** with relative intensity 80:10:10, respectively. The coupling constants between NH and ethylene protons for A and C forms were 11.6 and 14.4 Hz, respectively, whereas for form B the *^3^J* was 7.5 Hz which corresponding of the stereochemistry provided in Scheme 3. Only one type of signals for **5a** (R^5^ = CH_3_) was observed in the ^13^C-NMR spectrum.

The ^1^H-NMR for **5b** (R^5^ = CH_2_COOH) showed the presence of two isomers, **A** and **C**, with relative intensity 85:15, respectively. The coupling constants between NH and ethylene protons for **A** and **C** isomers were both 14.2 Hz, which corresponds to stereochemistry provided in Scheme 3. The NOESY spectrum has showed proximity between CH_2_ and ethylene = CH protons as an additional confirmation of the stereochemistry of **A** and **C** isomer which allows excluding the **B** isomer for this compound. 2D NMR data for **5b** showed that **A** and **C** isomers of **5b** exhibited the stereochemistry similar to those for compound **5c** (R^5^ = cyclopropyl).

Taking into account the similarity in chemical shift values for **A** and **C** forms, the structure **A** can be proposed as the main isomer for all derivatives **5a–h**, **C** as a minor isomer and, no **B** form in most cases (it appears only in **5a** and **5c**).

Isomer A of **5a–c** showed either the presence of AX system with *^3^J* = 11.6…14.2 Hz for NH proton and for the proton of the ethane moiety or two broad singlets for these protons. This can be due to the velocity of **A** > **B** transformation which may be susceptible to different factors such as concentration, trace of water, etc.

To evaluate the presence of the rotamer forms we have studied the dependence of the ^1^H-NMR spectra pattern for compound **5c** on the temperature. The tripling of the signals in the spectra of **5c** had been gradually lost at 60 °C until the signals coalesce completely at 100 °C, therefore, the complication of ^1^H-NMR spectra is connected with hindered rotation. Compounds **2a–l**, **6a–d** showed ^1^H- and ^13^C-NMR data similar to those for **A** isomer of **5a–h**, although no traces of additional stereoisomers were found.

### 2.3. The X-ray Crystal Structure Analysis of (Z)-5-Cyclopropylaminomethylidene-[1,3]thiazolo-[3,2-b][1,2,4]triazol-6(5H)-one *(**5c**)*

**Figure 5:**
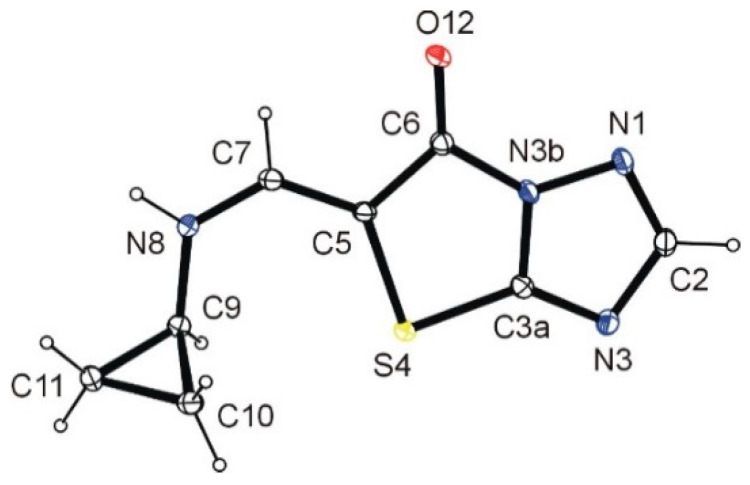
A view of **5c**, showing the atomic labelling scheme. Non-H atoms are drawn as 30% probability displacement ellipsoids and H atoms are drawn as spheres of an arbitrary size.

The molecular structure of **5c** and the atom-labelling scheme are illustrated in Figure 5. The molecule consists of the eight-membered thiazolo[3,2-*b*][1,2,4]triazol-6(5*H*)-one system and the *N*-cyclopropylaminomethylidene residue located at the C5 atom. The fused bicyclic system is approximately planar with an r.m.s. deviation of 0.0245 Å. The interatomic C5–C7 distance observed [1.373(2) Å] confirms the presence of a double bond between these atoms. The *N*-cyclopropylamino moiety is in *Z* configuration with respect to the bicyclic moiety. The torsion angle S4–C5–C7–N8 is −0.1(3)°.

The molecules of **5c** highlight the presence of quite strong resonance effect involving the carbonyl group and the exocyclic N8 atom via the double bond C5–C7. This is indicated by the C6–O12 [1.2293(19) Å], C5–C6 [1.438(2) Å], C5–C7 [1.373(2) Å], and C7-N8 [1.320(2) Å] bonds which are all significantly distorted with respect to the typical lengths of the C*=*O [1.210(1) Å], C*sp*^2^–C*sp*^2^ [1.478(4) Å], C=C [1.331(1) Å] and C*sp*^2^–N [1.355(3) Å] bonds [36].

In the crystal of **5c**, the molecules related by screw axis 2_1_ are connected by the classical N8–H8∙∙∙O12^i^ hydrogen bonds into chains (Supplementary Figure S2a, Table 1). It is worth noting that in the chains, the molecules, or rather their planar thiazolo[3,2-*b*][1,2,4]triazol-6(5*H*)-one ring systems, are arranged coplanar to each other to form tapes with the best plane parallel to the plane (−203). The adjacent anti-parallel chains of molecules are further joined by hydrogen bonds C11–H11A∙∙∙N3^ii^ into layers showing a stepped shape, with mean plane parallel to the plane (−102) (Supplementary Figure S2b, Table 1). There are π-electron interactions between the adjacent layers (Supplementary Figure S3). The perpendicular distances between partially overlapping thiazolo[3,2-*b*][1,2,4]triazol-6(5*H*)-one (ThiTri) systems are 3.4409 (6) Å and 3.4100 (6) Å for π (ThiTri)∙∙∙ π (ThTr)^iii^ and π(ThiTri)∙∙∙ π (ThTr)^iv^, respectively [symmetry codes: (iii) x, 3/2 − *y*, ½ + *z*, (iv) x, 3/2 − *y*,−1/2 + *z*].

### 2.4. In Vitro Evaluation of the Anticancer Activity and Cytotoxicity. Compare Analysis

Compounds **2a**, **2c**–**f**, **2h**–**l**, **3**, **5e**, **5f** were selected for antitumor activity which was performed according to the standard protocols of National Cancer Institute (NCI, Bethesda, MD, USA) Developmental Therapeutic Program (DTP) [37,38,39,40]. At the prescreening stage, the compounds were evaluated for antitumor activity at the concentration of 10 µM against a panel of approximately sixty cancer cell lines representing different types of cancer including leukemia, melanoma, lung, colon, CNS, ovarian, renal, prostate and breast cancers. The results of the prescreening assay are summarized in Table 2.

Overall, the 5-ene-thiazolo[3,2-*b*][1,2,4]triazole-6(5*H*)-ones exhibited a range of potencies against the tumor cell lines tested. Among them, three compounds (**2d**, **2h**, **2i**) presented excellent cytotoxic and four derivatives (**2d**, **2h**, **2i**, **2k**) cytostatic properties in one or more cell lines when applied at a concentration of 10 µM. It worth noting that compounds **2d**, **2h**, **2i** had been possessing cytotoxic effect against leukemia cell lines. Also, compound **2h** has demonstrated a cytotoxic effect on some lines in prostate cancer, NSCLC, breast cancer, renal cancer, melanoma, and CNS cancer subpanels. Since compounds **2h** and **2i** exhibited the highest cytotoxic and cytostatic activity, they were selected for in-depth studies at five different concentrations (0.01, 0.1, 1.0, 10.0, and 100.0 μM) against the same NCI-60 HTCL panel of cell lines. The outcomes were calculated and presented in the form of three response parameters (GI_50_, TGI and LC_50_) for each cell line via growth percentage inhibition curves [41,42]. The GI_50_ value (growth inhibitory activity) corresponds to the concentration of compound causing 50% decrease in net cell growth, the TGI value (cytostatic activity) represents the concentration of compound resulting in total growth inhibition (100% growth inhibition) and LC_50_ value (cytotoxic activity) demonstrate the lethal dose of compound causing net 50% death of initial cells. The results calculated are provided in Supplementary Table S2 and Table 3.

Compound **2h** inhibited each of the 49 cancer cell lines tested in the micromolar range with GI_50_ < 10 μM and average GI_50_, TGI and LC_50_ values of 3.54 μM, 12.88 μM, and 37.15 μM, respectively (Table 3). The moderate selectivity of **2h** was observed for breast cancer cell line MCF7 at GI_50_ (SI = 4.48) and TGI (SI = 3.19) levels, and for leukemia lines: SR at TGI (SI = 3.16), CCRF-CEM at TGI (SI = 4.08) and LC_50_ (SI = 4.90) levels. In stark contrast, compound **2i** exhibited lower inhibitory activity with GI_50_ < 10 μM against 13 cell lines with average GI_50_, TGI, and LC_50_ values of 10.96 μM, 28.18 μM and 63.09 μM, respectively. A moderate selectivity was observed at GI_50_ level for leukemia line CCRF-CEM (SI = 3.81) and breast cancer cell line HS 578T (SI = 3.01).

**Figure 6:**
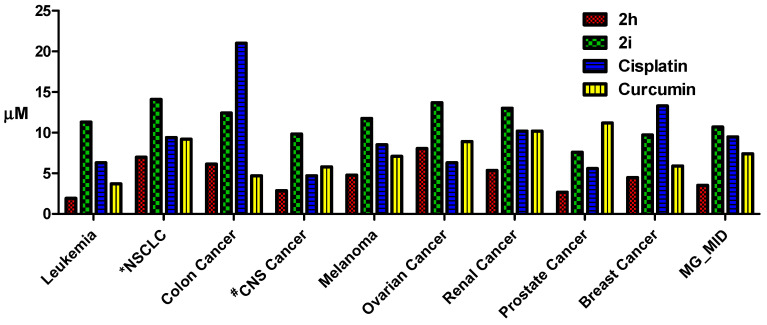
Mean growth inhibitory concentration (MG_MID GI_50_, µM) of compounds **2h** and **2i** in comparison with cisplatin and curcumin. *NSCLC—Non-small Cell Lung Cancer, ^#^CNS—Central nervous system.

The mean GI_50_ values (µM) for compounds **2h** and **2i** have been compared to the same data for standard anticancer agents as the synthetic drug cisplatin and the natural compound curcumin (Figure 6). The data demonstrates that MG_MID (µM) values for **2h** are lower than those for cisplatin except in ovarian cancer subpanel and for curcumin except in the colon cancer subpanel when tested in the same experimental setup. Meanwhile, compound **2i** showed lower activity and its MG_MID (µM) values were superior only for the colon and breast cancer subpanels in comparison with cisplatin and for the prostate cancer subpanel in comparison with curcumin.

The COMPARE algorithm of the NCI allows the prediction of biochemical mechanisms of action of the novel compounds on the basis of their in vitro activity profiles by comparing these to the ones of standard agents. The COMPARE analysis was performed for **2h** and **2i** against the NCI “Standard Agents” database at the GI_50_ level [38,39,40]. The values of the Pearson correlation coefficients (PCC) higher than 0.5 were chosen as noteworthy. However, PCC values obtained did not enable the prediction of the mechanisms of cytotoxicity for the compounds tested with high probability. Both compounds showed the correlation at the GI_50_ level only with one NCI Standard Agent–“Glycoxalic acid” (NSC:S267213, chemical name: (*E*)-2-(2-((4-methoxyphenyl)sulfonyl) hydrazono)acetic acid or glyoxylic acid *p*-methoxybenzenesulfonylhydrazone) and the PCC values were calculated as 0.561 for **2h** and 0.647 for **2i**. The alkylating mechanism of antineoplastic action has been reported for NSC:S267213 and a set of its structural analogs [43,44,45,46]

Compounds **2h** and **2i** were also evaluated in vitro for their cytotoxic potential against human embryonic kidney (HEK 293) cell lines using the MTT assay [47]. The IC_50_ values obtained were 28.99 µM and 24.43 µM for **2h** and **2i**, respectively, suggest that these derivatives could effectively act against cancer cell lines without causing toxicity to normal somatic cells.

### 2.5. The Preliminary Structure-Anticancer Activity Relationship for Novel 5-Ene-thiazolo[3,2-b][1,2,4]triazole-6(5H)-ones

Based on the biological data obtained so far, structure-activity relationship (SAR) analysis was determined. The SAR data revealed that the anticancer activity of synthesized 5-ene-thiazolo[3,2-*b*][1,2,4]triazole-6(5*H*)-ones is dependent upon the nature of substituent connected with a double bond at C-5 of the bicyclic system. Presence of C-5 linked benzylidene moieties resulted in better activity compared to heterocyclic rings (furan (**2j**), thiophene (**2k**), *N*-AcO-indole (**2l**)). Besides it, the presence and position of a chlorine atom in the benzylidene part of molecules also plays a crucial role in the realization of the anticancer effect. So, the derivatives **2h** and **2i** with the chlorine atom and with an additional modification by *N*-(4-R^4^-phenyl)acetamides fragments linked at the O-2 position have been found to exert excellent antitumor activities. It is worth to note that the introduction of an imine-linker between the 4-chlorophenyl moiety and double bond at C-5 (**5f**), drastically decreased the inhibitory activity compared with data reported in [29]. Similarly, the moving of the fluorine atom from C-4 to C-2 position in benzylidene moiety (**2a**) leads to loss of activity [29]. Interestingly, that derivative containing 4-AcO-group in benzylidene core (**2d**), demonstrated inhibition of leukemia lines CCRF-CEM (−73,92), HL-60 (TB) (−58,44), MOLT-4 (−73,62) against the backdrop of the almost complete lack of impact on other cell lines. In addition, it should be noted that for all active derivatives screened at both stages, the activity against leukemia line(s) was observed which is in agreement with data described in the literature [27,29,30].

## 3. Materials and Methods

### 3.1. General Information

Melting points were measured in open capillary tubes on a BÜCHI B-545 melting point apparatus (BÜCHI Labortechnik AG, Flawil, Switzerland) and are uncorrected. The elemental analyses (C, H, N) were performed using the Perkin–Elmer 2400 CHN analyzer (PerkinElmer, Waltham, MA, USA) and were within ±0.4% of the theoretical values. The 500 MHz-^1^H and 126 MHz-^13^C spectra were recorded on Bruker AVANCE-500 spectrometer and 2D spectra were recorded on a Bruker AVANCE-600 spectrometer (Bruker, Bremen, Germany). All spectra were recorded at room temperature except where indicated otherwise and were referenced internally to solvent reference frequencies. Chemical shifts (δ) are quoted in ppm and coupling constants (J) are reported in Hz. LC-MS spectra were obtained on a Finnigan MAT INCOS-50 (Thermo Finnigan LLC, San Jose, CA, USA). Solvents and reagents that are commercially available were used without further purification. The synthetic procedure for compound **1** was described in [48], for derivatives **2a–l** in [29] and for compound **4** in [35].

### 3.2. Preparation and Characterization of Compounds

#### 3.2.1. Characterization of Compounds **2a**–**l**

*(Z)-5-(2-Fluorobenzylidene)thiazolo[3,2-b]*[1,2,4]*triazol-6(5H)-one* (**2a**). Yield 58%, light yellow powder, mp 192–193 °C (ethanol–DMF 4:1). ^1^H-NMR (500 MHz, DMSO-*d*_6_, δ): 8.53 (s, 1H, 2-H), 8.19 (s, 1H, =CH), 7.73 (t, *J* = 8.5 Hz, 1H, C_6_H_4_), 7.64–7.69 (m, 1H, C_6_H_4_), 7.46–7.49 (m, 2H, C_6_H_4_). ^13^C-NMR (126 MHz, DMSO-*d*_6_, δ): 161.5, 159.4, 155.7, 134.3, 130.1, 129.2, 127.2, 125.5, 119.9, 116.5, 116.2. LCMS (ESI+) *m*/*z* 248 [M + H]^+^. Anal. calc. for C_11_H_6_FN_3_OS: C 53.44%, H 2.45%, N 16.99%. Found: C 53.60%, H 2.70%, N 17.20%.

*(Z)-5-(2-Methoxybenzylidene)thiazolo[3,2-b]*[1,2,4]*triazol-6(5H)-one* (**2b**). Yield 54%, yellow powder, mp 196–198 °C (ethanol–DMF 4:1). ^1^H-NMR (500 MHz, DMSO-*d*_6_, δ): 8.49 (s, 1H, 2-H), 8.34 (s, 1H, =CH), 7.62–7.56 (m, 2H, C_6_H_4_), 7.22 (d, *J* = 8.0 Hz, 1H, C_6_H_4_), 7.16 (t, *J* = 7.5 Hz, 1H, C_6_H_4_), 3.95 (s, 3H, CH_3_). ^13^C-NMR (126 MHz, DMSO-*d*_6_, δ): 160.0, 159.1, 158.5, 156.5, 134.9, 134.1, 130.2, 124.7, 121.4, 120.7, 112.1, 69.1, 55.9. LCMS (ESI+) *m*/*z* 260 [M + H]^+^. Anal. calc. for C_12_H_9_N_3_O_2_S: C 55.59%, H 3.50%, N 16.21%. Found: C 55.80%, H 3.70%, N 16.40%.

*(Z)-5-(2-(Allyloxy)benzylidene)thiazolo[3,2-b]*[1,2,4]*triazol-6(5H)-one* (**2c**). Yield 63%, brown-yellow powder, mp 133–136 °C (ethanol). ^1^H-NMR (500 MHz, DMSO-*d*_6_, δ): 8.50 (s, 1H, 2-H), 8.40 (s, 1H, =CH), 7.61 (d, *J* = 9.3 Hz, 1H, C_6_H_4_), 7.59–7.54 (m, 1H, C_6_H_4_), 7.23 (d, *J* = 8.5 Hz, 1H, C_6_H_4_), 7.17 (t, *J* = 7.3 Hz, 1H, C_6_H_4_), 6.12 (ddt, *J* = 15.8, 10.5, 5.2 Hz, 1H, CH_2_=CH-CH_2_), 5.45 (d, *J* = 1.7 Hz, 1H, CH_2_=CH-CH_2_), 5.34 (d, *J* = 1.6 Hz, 1H, CH_2_=CH-CH_2_), 4.77–4,79 (m, 2H, CH_2_=CH-CH_2_). ^13^C-NMR (126 MHz, DMSO-*d*_6_, δ): 159.8, 159.0, 157.4, 156.3, 134.1, 133.8, 132.7, 129.7, 124.4, 121.5, 120.6, 118.1, 113.4, 68.9. LCMS (ESI+) *m*/*z* 286 [M + H]^+^. Anal. calc. for C_14_H_11_N_3_O_2_S: C 58.93%, H 3.89%, N 14.73%. Found: C 59.10%, H 4.00%, N 14.90%.

*(Z)-4-((6-Oxothiazolo[3,2-b]*[1,2,4]*triazol-5(6H)-ylidene)methyl)phenyl acetate* (**2d**). Yield 53%, white-yellow powder, mp 211–213 °C (ethanol–DMF 4:1). ^1^H-NMR (500 MHz, DMSO-*d*_6_, δ): 8.70 (s, 1H, =CH), 8.47 (s, 1H, 2-H), 8.01 (d, *J* = 8.5 Hz, 2H, C_6_H_4_), 7.58 (d, *J* = 8.6 Hz, 2H, C_6_H_4_), 2.50 (s, 3H, CH_3_). ^13^C-NMR (126 MHz, DMSO-*d*_6_, δ): 169.0, 159.5, 159.2, 156.0, 152.7, 138.5, 132.0, 129.9, 124.5, 123.2, 49.7, 20.8. LCMS (ESI+) *m*/*z* 288 [M + H]^+^. Anal. calc. for C_13_H_9_N_3_O_3_S: C 54.35%, H 3.16%, N 14.63%. Found: C 54.50%, H 3.40%, N 14.80%.

*(Z)-5-(4-(Diethylamino)benzylidene)thiazolo[3,2-b]*[1,2,4]*triazol-6(5H)-one* (**2e**). Yield 67%, light red-purple powder, mp 232–234 °C (ethanol–DMF 4:1). ^1^H-NMR (500 MHz, DMSO-*d*_6_, δ): 8.46 (s, 1H, 2-H), 8.10 (s, 1H, =CH), 7.60 (d, *J* = 9.1 Hz, 2H, C_6_H_4_), 6.86 (d, *J* = 9.1 Hz, 2H, C_6_H_4_), 3.47 (d, *J* = 7.1 Hz, 4H, 2*CH_2_), 1.14 (t, *J* = 7.0 Hz, 6H, 2*CH_3_). ^13^C-NMR (126 MHz, DMSO-*d*_6_, δ): 158.8, 158.6, 156.5, 150.5, 141.1, 133.9, 118.5, 114.4, 111.9, 44.1, 12.6. LCMS (ESI+) *m*/*z* 301 [M + H]^+^. Anal. calc. for C_15_H_16_N_4_OS: C 59.98%, H 5.37%, N 18.65%. Found: C 60.20%, H 5.50%, N 18.80%.

*(Z)-5-(3,4-Dimethoxybenzylidene)thiazolo[3,2-b]*[1,2,4]*triazol-6(5H)-one* (**2f**). Yield 53%, yellow powder, mp 207–209 °C (ethanol–DMF 4:1). ^1^H-NMR (500 MHz, DMSO-*d*_6_, δ): 8.50 (s, 1H, 2-H), 8.23 (s, 1H, =CH), 7.40 (dd, *J* = 7.4, 2.2 Hz, 1H, C_6_H_3_), 7.36 (d, *J* = 2.1 Hz, 1H, C_6_H_3_), 7.21 (d, *J* = 8.5 Hz, 1H, C_6_H_3_), 3.87 (s, 3H, CH_3_), 3.85 (s, 3H, CH_3_). ^13^C-NMR (126 MHz, DMSO-*d*_6_, δ): 159.3, 159.0, 156.6, 152.4, 152.0, 149.2, 140.4, 125.2, 121.3, 114.0, 112.3, 55.8, 55.5. LCMS (ESI+) *m*/*z* 290 [M + H]^+^. Anal. calc. for C_13_H_11_N_3_O_3_S: C 53.97%, H 3.83%, N 14.52%. Found: C 54.10%, H 4.00%, N 14.80%.

*(Z)-2-(4-Chloro-2-((6-oxothiazolo[3,2-b]*[1,2,4]*triazol-5(6H)-ylidene)methyl)phenoxy)-N-(m-tolyl)acetamide* (**2g**). Yield 68%, white-yellow powder, mp 260–262 °C (ethanol–DMF 4:1). ^1^H-NMR (500 MHz, DMSO-*d*_6_, δ): 10.18 (s, 1H, NH), 8.52 (s, 1H, 2-H), 8.40 (s, 1H, =CH), 7.64–7.58 (m, 2H, C_6_H_4_ + C_6_H_3_), 7.44 (brs, 1H, C_6_H_4_ + C_6_H_3_), 7.36 (d, *J* = 8.1 Hz, 1H, C_6_H_4_ + C_6_H_3_), 7.24–7.08 (m, 2H, C_6_H_4_ + C_6_H_3_), 6.90 (d, *J* = 7.4 Hz, 1H, C_6_H_4_+C_6_H_3_), 4.97 (s, 2H, CH_2_), 2.27 (s, 3H, CH_3_). ^13^C-NMR (126 MHz, DMSO-*d*_6_, δ): 165.5, 159.7, 156.1, 155.9, 138.2, 138.0, 133.5, 132.8, 128.8, 128.6, 126.8, 125.4, 124.4, 122.9, 119.9, 119.9, 116.6, 115.2, 67.5, 21.2. LCMS (ESI+) *m*/*z* 428 [M + H]^+^. Anal. calc. for C_20_H_15_ClN_4_O_3_S: C 56.27%, H 3.54%, N 13.12%. Found: C 56.50%, H 3.70%, N 13.30%.

*(Z)-2-(4-Chloro-2-((6-oxothiazolo[3,2-b]*[1,2,4]*triazol-5(6H)-ylidene)methyl)phenoxy)-N-(4-chlorophenyl)acetamide* (**2h**). Yield 58%, white-yellow powder, mp 224–226 °C (ethanol–DMF 4:1)^1^H-NMR (500 MHz, DMSO-*d*_6_, δ): 10.39 (s, 1H, NH), 8.50 (s, 1H, 2-H), 8.39 (s, 1H, =CH), 7.70–7.54 (m, 4H, C_6_H_4+_C_6_H_3_), 7.38 (d, *J* = 8.9 Hz, 2H, C_6_H_4+_C_6_H_3_), 7.18 (d, *J* = 8.8 Hz, 1H, C_6_H_4+_C_6_H_3_), 4.99 (s, 2H, CH_2_).^13^C-NMR (126 MHz, DMSO-*d*_6_, δ): 166.0, 159.7, 159.4, 156.1, 155.8, 137.4, 133.5, 132.8, 128.8, 128.7, 127.4, 126.9, 125.5, 122.9, 121.1, 115.3, 67.2.LCMS (ESI+) *m*/*z* 449 [M + H]^+^. Anal. calc. for C_19_H_12_Cl_2_N_4_O_3_S: C 51.02%, H 2.70%, N 12.53%. Found: C 51.20%, H 2.90%, N 12.70%.

*Ethyl(Z)-4-(3-(4-chloro-2-((6-oxothiazolo[3,2-b]*[1,2,4]*triazol-5(6H)-ylidene)methyl)phenyl)propanamido)benzoate* (**2i**). Yield 52%, white-yellow powder, mp 176–178 °C (ethanol–DMF 4:1). ^1^H-NMR (500 MHz, DMSO-*d*_6_, δ): 10.60 (s, 1H, NH), 8.51 (s, 1H, 2-H), 8.39 (s, 1H, =CH), 7.93 (d, *J* = 8.9 Hz, 2H, C_6_H_4+_C_6_H_3_), 7.74 (d, *J* = 8.8 Hz, 2H, C_6_H_4+_C_6_H_3_), 7.57–7.64 (m, 2H, C_6_H_4+_C_6_H_3_), 7.20 (d, *J* = 8.7 Hz, 1H, C_6_H_4+_C_6_H_3_), 5.04 (s, 2H, CH_2_), 4.28 (q, *J* = 7.1 Hz, 2H, CH_2_), 1.30 (t, *J* = 7.1 Hz, 3H, CH_3_). ^13^C-NMR (126 MHz, DMSO-*d*_6_, δ): 172.0, 166.3, 165.4, 159.7, 159.4, 156.1, 155.8, 142.6, 133.6, 132.5, 130.4, 128.8, 126.8, 125.5, 124.6, 122.9, 118.8, 115.1, 67.5, 60.6, 21.1, 14.4. LCMS (ESI+) *m*/*z* 486 [M + H]^+^. Anal. calc. for C_22_H_17_ClN_4_O_5_S: C 54.49%, H, 3.53%, N 11.55%. Found: C 54.70%, H 3.80%, N 11.70%.

*(Z)-5-(Furan-2-ylmethylene)thiazolo[3,2-b]*[1,2,4]*triazol-6(5H)-one* (**2j**). Yield 61%, brown-yellow powder, mp 230–232 °C (ethanol–DMF 4:1). ^1^H-NMR (500 MHz, DMSO-*d*_6_, δ): 8.47 (s, 1H, 2-H), 8.22 (d, *J* = 2.3 Hz, 1H, furan), 8.13 (s, 1H, =CH), 7.35 (d, *J* = 4.0 Hz, 1H, furan), 6.86 (dd, *J* = 3.6, 1.8 Hz, 1H). ^13^C-NMR (126 MHz, DMSO-*d*_6_, δ): 160.1, 159.2, 156.3, 149.3, 148.8, 125.7, 122.0, 120.8, 114.1. LCMS (ESI+) *m*/*z* 220 [M + H]^+^. Anal. calc. for C_9_H_5_N_3_O_2_S: C 49.31%, H 2.30%, N 19.17%. Found: C 49.50%, H 2.50%, N 19.30%.

*(Z)-5-(Thiophen-2-y-lmethylene)thiazolo[3,2-b]*[1,2,4]*triazol-6(5H)-one* (**2k**). Yield 64%, brown powder, mp > 250 °C (ethanol–DMF 4:1). ^1^H-NMR (500 MHz, DMSO-*d*_6_, δ): 8.57 (s, 1H, =CH), 8.50 (s, 1H, 2-H), 8.19 (d, *J* = 7.0 Hz, 1H, thiophen), 7.90 (d, *J* = 3.1 Hz, 1H, thiophen), 7.39 (dd, *J* = 3.7 Hz, 1H, thiophen). ^13^C-NMR (126 MHz, DMSO-*d*_6_, δ): 159.3, 156.2, 137.1, 136.7, 136.2, 135.3, 135.2, 132.7, 129.6. LCMS (ESI+) *m*/*z* 236 [M + H]^+^. Anal. calc. for C_9_H_5_N_3_OS_2_: C 45.94%, H 2.14%, N 17.86%. Found: C 46.10%, H 2.30%, N 18.00%.

*(Z)-5-((1-Acetyl-1H-indol-3-yl)methylene)thiazolo[3,2-b]*[1,2,4]*triazol-6(5H)-one* (**2l**). Yield 66%, dark-yellow powder, mp > 250 °C (ethanol–DMF 4:1). ^1^H-NMR (500 MHz, DMSO-*d*_6_, δ): 8.37–8.43 (m, 3H, 2-H, =CH, indole), 8.15 (brs, 1H, indole), 7.91–8.11 (m, 1H, indole), 7.38–7.45 (m, 2H, indole), 2.82 (s, 3H, CH_3_). ^13^C-NMR (126 MHz, DMSO-*d*_6_, δ): 170.4, 166.4, 160.5, 159.2, 156.6, 135.5, 130.6, 130.0, 126.9, 125.1, 124.4, 119.5, 116.7, 114.6, 23.4. LCMS (ESI+) *m*/*z* 311 [M + H]^+^. Anal. calc. for C_15_H_10_N_4_O_2_S: C 58.06; H 3.25; N 18.05%. Found: C 58.30%, H 3.50%, N 18.20%.

#### 3.2.2. Synthesis of 2-((1H-1,2,4-Triazol-3-yl)thio)acetic Acid (**3**)

A mixture of compound **1** (10 mmol) with the chloroacetic acid (10 mmol) and anhydrous sodium acetate was refluxed for 2 h in glacial acetic acid (10 mL). The white solid obtained was filtered, washed with ethanol and recrystallized from glacial acetic acid. Yield 76%, white powder, mp 132–134 °C (acetic acid). ^1^H-NMR (500 MHz, DMSO-*d*_6_, δ): 13.99 (brs, NH), 12.80 (brs, COOH), 8.47 (brs, 2-H), 3.91 (2H, s, CH_2_). ^13^C-NMR (126 MHz, DMSO-*d*_6_, δ): 170.0, 158.1, 144.8, 33.7. LCMS (ESI+) *m*/*z* 160 [M + H]^+^. Anal. calc. for C_4_H_5_N_3_O_2_S: C 30.19; H 3.17; N 26.40. Found: C 30.30%, H 3.40%, N 26.60%.

#### 3.2.3. Synthesis and Characterization of Compounds **5a**–**h**, **6a**–**d**

A mixture of compound **4** (10 mmol) with the appropriate amine (10 mmol) was refluxed for 2 h in ethanol. The solid products obtained were filtered, washed with ethanol and recrystallized from ethanol or the mixture of ethanol–dimethylformamide (4:1).

*(E/Z)-5-((Methylamino)methylene)thiazolo[3,2-b]*[1,2,4]*triazol-6(5H)-one* (**5a**). Yield 53%, bright red-purple powder, mp 239–241 °C (ethanol–DMF 4:1). A form (~80%), ^1^H-NMR (500 MHz, DMSO-*d*_6_, δ): 8.76 (br s, 1H, NH), 8.29 (s, 1H, 2-H), 8.16 (d, *^3^J* = 11.6 Hz, 1H, =CH), 3.09 (3H, s, CH_3_). B form (~10%), ^1^H-NMR (500 MHz, DMSO-*d*_6_, δ): 8.69 (brs, 1H, NH), 8.30 (s, 1H, 2-H), 8.05 (d, *^3^J* = 7.5 Hz, 1H, =CH), 3.10 (s, 3H, CH_3_). C form (~10%), ^1^H-NMR (500 MHz, DMSO-*d*_6_, δ): 9.12–9.24 (m, 1H, NH), 8.33 (s, 1H, 2-H), 7.95 (d, *^3^J* = 14.4 Hz, 1H, =CH), 3.11 (s, 3H, CH_3_). ^13^C-NMR (126 MHz, DMSO-*d*_6_, δ): 157.7, 156.2, 155.9, 151.6, 88.5, 35.2. LCMS (ESI+) *m*/*z* 183 [M + H]^+^. Anal. calc. for C_6_H_6_N_4_OS: C 39.55%, H 3.32%, N 30.75%. Found: C 39.70%, H 3.50%, N 30.90%.

*(E/Z)-((6-Oxothiazolo[3,2-b]*[1,2,4]*triazol-5(6H)-ylidene)methyl)glycine* (**5b**). Yield 62%, yellow powder, mp 229 (with decomp.) °C (ethanol–DMF 4:1). A form (~85%), ^1^H-NMR (500 MHz, DMSO-*d*_6_, δ): 13.07 (brs, 1H, COOH), 8.95–9.08 (m, 1H, NH), 8.32 (s, 1H, 2-H), 8.17 (d, ^3^*J* = 14.2 Hz, 1H, =CH), 4.19 (d, ^3^*J* = 5.6 Hz, 2H, CH_2_). ^13^C-NMR (126 MHz, DMSO-*d*_6_, δ): 170.9 (COOH), 157.9 (C-2), 156.5 (C-6), 156.4 (C-3a), 151.6 (=CH), 89.95 (C-5), 49.0 (CH_2_). NOESY: 8.17–9.03 (=CH…HN), 4.19–8.17 (CH_2_…=CH), 4.19–9.03 (CH_2_…NH). C form, (~15%), ^1^H-NMR (500 MHz, DMSO-*d*_6_, δ): 13.07 (brs, 1H, OH), 9.22–9.29 (m, 1H, NH), 8.33 (s, 1H, 2-H), 7.91 (d, ^3^*J* = 14.2 Hz, 1H, =CH), 4.16 (d, ^3^*J* = 6.3 Hz, 2H, CH_2_). ^13^C-NMR (126 MHz, DMSO-*d*_6_, δ): 170.7 (COOH), 157.7 (C-2), 157.4 (C-3a), 156.1 (C-6), 154.9 (=CH), 88.9 (C-5), 49.7 (CH_2_). NOESY: 9.26–7.91 (weak, NH…HC=), 9.26–4.16 (NH…H_2_C), 4.16–7.91 (CH_2_…HC=). EXSY: 9.02–9.25. LCMS (ESI+) *m*/*z* 227 [M + H]^+^. Anal. calc. for C_7_H_6_N_4_O_3_S: C 37.17%, H 2.67%, N 24.77%. Found: C 37.40%, H 2.90%, N 24.90%.

*(E/Z)-5-((Cyclopropylamino)methylene)thiazolo[3,2-b]*[1,2,4]*triazol-6(5H)-one* (**5c**). Yield 54%, beige powder, mp 199–201 °C (ethanol–DMF 4:1). A form (~62%), ^1^H-NMR (500 MHz, DMSO-*d*_6_, δ): 9.04 (brs, 1H, NH), 8.31 (s, 1H, 2-H), 8.12 (brs, 1H, =CH), 3.04–3.10 (m, 1H, cyclopropyl), 0.87–0.98 (m, 1H, cyclopropyl), 0.66–0.80 (m, 3H, cyclopropyl).^13^C-NMR (126 MHz, DMSO-*d*_6_, δ): 157.9 (C-6), 157.8 (C-2), 156.2 (C-3*a*), 150.1 (=CH), 89.51 (C-5), 30.3 (cyclopropyl), 6.0 (cyclopropyl). NOESY: 8.14–9.05 (=CH…HN), 3.07–9.05 (NH…HC<), 3.07–8.14 (=CH…HC<), 0.72–9.05, 0.72–8.14, 0.72–3.07, 0.72–0.95. B form (~31%), ^1^H-NMR (500 MHz, DMSO-*d*_6_, δ): 8.88–8.98 (m, 1H, NH), 8.35 (s, 1H, 2-H), 7.97 (d, ^3^*J* = 7.3 Hz, 1H, =CH), 2.84–2.93 (m, 1H, cyclopropyl), 0.66–0.80 (m, 4H, cyclopropyl). ^13^C-NMR (126 MHz, DMSO-*d*_6_, δ): 157.9 (C-3a), 157.8 (C-2), 157.1 (C-6), 148.8 (=CH), 89.48 (C-5), 25.2 (cyclopropyl), 8.84 (cyclopropyl). C form (~7%), ^1^H-NMR (500 MHz, DMSO-*d*_6_, δ): 9.33–9.39 (m, 1H, NH), 8.06 (d, ^3^*J* = 13.9 Hz, 1H, =CH), 3.01–3.04 (m, 1H, cyclopropyl), 0.80–0.85 (m, 2H, cyclopropyl), 0.66–0.80 (m, 2H, cyclopropyl). ^13^C-NMR (126 MHz, DMSO-*d*_6_, δ): 157.6 (C-3a), 157.4 (C-2), 153.7 (C-6), 31.0 (cyclopropyl), 6.2 (cyclopropyl). EXSY: 9.36–8.93 (C-B), 9.36–9.06 (C-A), 8.93–9.06 (B-A), 8.06–7.97 (C-B), 8.14–8.06 (A-C), 7.97–8.14 (B-A), 2.87–3.07 (B-A). LCMS (ESI+) *m*/*z* 209 [M + H]^+^. Anal. calc. for C_8_H_8_N_4_OS: C 46.14%, H 3.87%, N 26.90%. Found: C 46.30%, H 4.00%, N 27.10%.

*(E/Z)-5-((Phenylamino)methylene)thiazolo[3,2-b]*[1,2,4]*triazol-6(5H)-one* (**5d**). Yield 56%, brown-yellow powder, mp 262–263 °C (ethanol–DMF 4:1). A form (~90%), ^1^H-NMR (500 MHz, DMSO-*d*_6_, δ): 10.76 (brs, 1H, NH), 8.52 (brs, 1H, =CH), 8.38 (s, 1H, 2-H), 7.37–7.42 (m, 4H, C_6_H_5_), 7.13–7.17 (m, 1H, C_6_H_5_). ^13^C-NMR (126 MHz, DMSO-*d*_6_, δ): 158.3, 156.9, 156.8, 155.5, 141.8, 129.7, 124.5, 117.1, 94.7. C form (~10%), ^1^H-NMR (500 MHz, DMSO-*d*_6_, δ): 10.72 (brs, 1H, NH), 8.68 (d, ^3^*J* = 13.6 Hz, 1H, =CH), 8.39 (s, 1H, 2-H), 7.37–7.42 (m, 4H, C_6_H_5_), 7.13–7.17 (m, 1H, C_6_H_5_). LCMS (ESI+) *m*/*z* 245 [M + H]^+^. Anal. calc. for C_11_H_8_N_4_OS: C 54.09%, H 3.30%, N 22.94%. Found: C 54.20%, H 3.50%, N 23.10%.

*(E/Z)-5-(((3-Hydroxyphenyl)amino)methylene)thiazolo[3,2-b]*[1,2,4]*triazol-6(5H)-one* (**5e**). Yield 55%, brown-yellow powder, mp 269–271 °C (ethanol–DMF 4:1). A form (~90%), ^1^H-NMR (500 MHz, DMSO-*d*_6_, δ): 10.68 (brs, 1H, NH), 9.69 (s, 1H, OH), 8.40 (brs, 1H, =CH), 8.38 (s, 1H, 2-H), 7.13–7.23 (m, 1H, C_6_H_4_), 6.78–6.85 (m, 1H, C_6_H_4_), 6.69–6.78 (m, 1H, C_6_H_4_), 6.52–6.62 (m, 1H, C_6_H_4_). ^13^C-NMR (126 MHz, DMSO-*d*_6_, δ): 158.5, 158.3, 157.0, 156.8, 141.5, 140.9, 130.6, 111.8, 107.6, 104.2, 94.5. C form (~10%), ^1^H-NMR (500 MHz, DMSO-*d*_6_, δ): 10.61 (brs, 1H, NH), 9.73 (s, 1H, OH), 8.63 (d, ^3^*J* = 12.6 Hz, 1H, =CH), 8.38 (s, 1H, 2-H), 6.84–6.88 (m, 2H, C_6_H_4_), 6.57–6.61 (m, 2H, C_6_H_4_). LCMS (ESI+) *m*/*z* 261 [M + H]^+^. Anal. calc. for C_11_H_8_N_4_O_2_S: C 50.76%, H 3.10%, N 21.53%. Found: C 50.90%, H 3.40%, N 21.70%.

*(E/Z)-5-(((4-Chlorophenyl)amino)methylene)thiazolo[3,2-b]*[1,2,4]*triazol-6(5H)-one* (**5f**). Yield 64%, yellow powder, mp > 280 °C (ethanol–DMF 4:1). A form (~90%), ^1^H-NMR (500 MHz, DMSO-*d*_6_, δ): 10.77 (1H, s, NH), 8.51 (brs, 1H, =CH), 8.40 (s, 1H, 2-H), 7.39–7.45 (m, 4H, C_6_H_4_). ^13^C-NMR (126 MHz, DMSO-*d*_6_, δ): 158.3, 157.0, 156.7, 155.6, 141.6, 129.5, 128.2, 118.9, 95.6. C form (~10%), ^1^H-NMR (500 MHz, DMSO-*d*_6_, δ): 10.74 (brs, 1H, NH), 8.62 (d, ^3^*J* = 13.2 Hz, 1H, =CH), 8.40 (s, 1H, 2-H), 7.49–7.52 (m, 2H, C_6_H_4_), 7.45–7.48 (m, 2H, C_6_H_4_). LCMS (ESI+) *m*/*z* 280 [M + H]^+^. Anal. calc. for C_11_H_7_ClN_4_OS: C 47.40%, H 2.53%, N 20.10%. Found: C 47.60%, H 2.70%, N 20.30%.

*(E/Z)-5-(((Furan-2-ylmethyl)amino)methylene)thiazolo[3,2-b]*[1,2,4]*triazol-6(5H)-one* (**5g**). Yield 71%, light pink powder, mp 176–178 °C (ethanol). A form (~90%), ^1^H-NMR (500 MHz, DMSO-*d*_6_, δ): 9.29 (s, 1H, NH), 8.30 (s, 1H, 2-H), 8.26 (brs, 1H, =CH), 7.66–7.69 (m, 1H, furan), 6.44–6.46 (m, 1H, furan), 6.41–6.44 (m, 1H, furan), 4.62 (s, 2H, CH_2_). ^13^C-NMR (126 MHz, DMSO-*d*_6_, δ): 157.9. 156.5, 156.3, 150.6, 150.3, 143.3, 110.7, 108.6, 89.8, 44.7. C form (~10%), ^1^H-NMR (500 MHz, DMSO-*d*_6_, δ): 9.43–9.50 (m, 1H, NH), 8.31 (s, 1H, 2-H), 8.06 (d, ^3^*J* = 13.9, 1H, =CH), 7.65–7.66 (m, 1H, furan), 6.39–6.41 (m, 1H, furan), 4.58 (d, ^3^*J* = 4.73, 2H, CH_2_). LCMS (ESI+) *m*/*z* 249 [M + H]^+^. Anal. calc. for C_10_H_8_N_4_O_2_S: C 48.38%, H 3.25%, N 22.57%. Found: C 48.50%, H 3.40%, N 22.80%.

*(E/Z)-5-(((5-Methylisoxazol-3-yl)amino)methylene)thiazolo[3,2-b]*[1,2,4]*triazol-6(5H)-one* (**5h**). Yield 54%, yellow powder, mp 243–245 °C (ethanol–DMF 4:1). A form (~95%) ^1^H-NMR (500 MHz, DMSO-*d*_6_, δ): 11.32 (brs, 1H, NH), 8.40 (s, 1H, 2-H), 8.32 (brs, 1H, =CH), 6.41 (s, 1H, isoxazol), 2.39 (s, 3H, CH_3_). ^13^C-NMR (126 MHz, DMSO-*d*_6_, δ): 171.2, 159.9, 158.7, 157.7, 156.8, 140.5, 98.5, 94.2, 12.3. C form (~5%) ^1^H-NMR (500 MHz, DMSO-*d*_6_, δ): 10.79 (d, ^3^*J* = 13.2 Hz, 1H, NH), 8.46 (d, ^3^*J* = 13.2 Hz, 1H, =CH). LCMS (ESI+) *m*/*z* 250 [M + H]^+^. Anal. calc. for C_9_H_7_N_5_O_2_S: C 43.37%, H 2.83%, N 28.10%. Found: C 43.50%, H 3.00%, N 28.30%.

*(Z)-5-(Indolin-1-ylmethylene)thiazolo[3,2-b]*[1,2,4]*triazol-6(5H)-one* (**6a**). Yield 57%, yellow-brown powder, mp > 250 °C (ethanol–DMF 4:1). ^1^H-NMR (500 MHz, DMSO-*d*_6_, δ): 8.62 (s, 1H, =CH), 8.43 (s, 1H, 2-H), 7.58 (d, *J* = 8.1 Hz, 1H, C_6_H_4_), 7.36 (d, *J* = 7.3 Hz, 1H, C_6_H_4_), 7.29 (t, *J* = 7.7 Hz, 1H, C_6_H_4_), 7.14 (t, *J* = 7.3 Hz, 1H, C_6_H_4_), 4.42 (t, *J* = 8.0 Hz, 2H, CH_2_), 3.37 (t, *J* = 8.0 Hz, 2H, CH_2_). ^13^C-NMR (126 MHz, DMSO-*d*_6_, δ): 158.3, 157.8, 156.7, 142.3, 138.9, 131.5, 128.1, 126.1, 125.0, 111.0, 93.1, 49.2, 27.7. LCMS (ESI+) *m*/*z* 271 [M + H]^+^. Anal. calc. for C_13_H_10_N_4_OS: C 57.76%, H 3.73%, N 20.73%. Found: C 57.90%, H 3.90%, N 20.90%.

*(Z)-5-(Piperidin-1-ylmethylene)thiazolo[3,2-b]*[1,2,4]*triazol-6(5H)-one* (**6b**). Yield 61%, beige powder, mp 190–192 °C (ethanol). ^1^H-NMR (500 MHz, DMSO-*d*_6_, δ): 8.35 (s, 1H, 2-H), 8.12 (s, 1H, =CH), 3.64 (s, 4H, piperidine), 1.65 (s, 6H, piperidine). ^13^C-NMR (126 MHz, DMSO-*d*_6_, δ): 157.8, 157.1, 156.7, 149.1, 148.7, 96.8, 86.2, 22.9. LCMS (ESI+) *m*/*z* 237 [M + H]^+^. Anal. calc. for C_10_H_12_N_4_OS: C 50.83%, H 5.12%, N 23.71%. Found: C 51.10%, H 5.40%, N 23.90%.

*(Z)-5-((4-(2-Hydroxyethyl)piperazin-1-yl)methylene)thiazolo[3,2-b]*[1,2,4]*triazol-6(5H)-one* (**6c**). Yield 67%, light pink powder, mp 189–192 °C (ethanol). ^1^H-NMR (500 MHz, DMSO-*d*_6_, δ): 8.35 (s, 1H, 2-H), 8.13 (s, 1H, =CH), 4.47 (t, *J* = 5.3 Hz, 1H, OH), 3.65 (d, *J* = 5.6 Hz, 4H, piperazine), 3.52 (q, *J* = 6.0 Hz, 2H, CH_2_), 2.58 (dd, *J* = 6.1, 4.2 Hz, 4H, piperazine), 2.45 (t, *J* = 6.1 Hz, 2H, CH_2_). ^13^C-NMR (126 MHz, DMSO-*d*_6_, δ): 157.9, 157.0, 156.0, 155.8, 148.9, 86.8, 59.7, 59.2, 58.4. LCMS (ESI+) *m*/*z* 282 [M + H]^+^. Anal. calc. for C_11_H_15_N_5_O_2_S: C 46.96%, H 5.37%, N 24.89%. Found: C 47.10%, H 5.60%, N 25.00%.

*Ethyl(Z)-4-((6-oxothiazolo[3,2-b]*[1,2,4]*triazol-5(6H)-ylidene)methyl)piperazine-1-carboxylate* (**6d**). Yield 62%, beige powder, mp 204–206 °C (ethanol–DMF 4:1). ^1^H-NMR (500 MHz, DMSO-*d*_6_, δ): 8.37 (s, 1H, 2-H), 8.19 (s, 1H, =CH), 4.07 (q, *J* = 7.1 Hz, 2H, CH_2_), 3.69 (brs, 4H, piperazine), 3.55 (brs, 4H, piperazine), 1.20 (t, *J* = 7.1 Hz, 3H, CH_3_). ^13^C-NMR (126 MHz, DMSO-*d*_6_, δ): 158.1, 157.3, 156.2, 154.5, 149.2, 111.6, 87.5, 61.2, 14.5. LCMS (ESI+) *m*/*z* 310 [M + H]^+^. Anal. calc. for C_12_H_15_N_5_O_3_S: C 46.59%, H 4.89%, N 22.64%. Found: C 46.90%, H 5.00%, N 22.80%.

### 3.3. Crystal Structure Determination of (E/Z)-5-((Cyclopropylamino)methylenethiazolo-[3,2-b]-[1,2,4]-triazol-6(5H)-one *(**5c**)*

#### 3.3.1. Crystal Data

C_8_H_8_N_4_OS, Mr = 208.24, monoclinic, space group P2_1_/c, a = 9.8291(4), b = 12.1556(4), c = 7.4814(3) Å, β = 95.025(4)°, V = 890.44(6) Å^3^, Z = 4, D_calc_ = 1.553 g/cm^3^, µ = 3.005 mm^−1^, T = 130.0(1) K.

#### 3.3.2. Data Collection

A yellow lath crystal (MeOH) of 0.24 × 0.13 × 0.03 mm was used to record 9342 (Cu *Ka*-radiation, *θ*_max_ = 76.52°) intensities on a SuperNova Dual Atlas diffractometer (Rigaku, Oxford, UK) [49] using mirror monochromatized Cu *Kα* radiation from a high-flux microfocus source (*λ* = 1.54178 Å). Accurate unit cell parameters were determined by least-squares techniques from the *θ* values of 3457 reflections, *θ* range 4.48–76.30°. The data were corrected for Lorentz polarization and for absorption effects [49]. The 1850 total reflections (*R*_int_ = 0.034) were used for structure determination.

#### 3.3.3. Structure Solution and Refinement

The structure was solved was solved by dual-space algorithm (SHELXT) [50], and refined against *F*^2^ for all data (SHELXL-97) [51]. The positions of the H atom bonded to N atom were obtained from the difference Fourier map and were refined freely. The remaining H atoms were placed geometrically in calculated positions and were refined with a riding model, with C–H = 0.99 Å (CH_2_), 1.00 Å (C*sp*^3^H), 0.95 Å (C*sp*^2^H) and *U*iso(H) = 1.2*U*eq(C). Final refinement converged with *R* = 0.0305 (for 1639 data with *F*^2^ > 4*σ*(*F*^2^)), wR = 0.0836 (on *F*^2^ for all data), and *S* = 1.105 (on *F*^2^ for all data). The largest difference peak and hole was 0.322 and −0.250 eÅ^3^. The molecular illustrations were drawn using ORTEP-3 for Windows [52]. Software used to prepare material for publication was WINGX [52], OLEX [53] and PLATON [54].

The supplementary crystallographic data of **5c** have been deposited at the Cambridge Crystallography Data Centre (CCDC) as supplementary publication CCDC2046355. Copies of the data can be obtained, free of charge, on application to CCDC, 12 Union Road, Cambridge CB2 1EZ, UK, (Fax: +44-(0)1223–336033 or e-mail: deposit@ccdc.cam.ac.uk).

### 3.4. Anticancer Activity Screening (NCI-60 Human Tumor Cell Lines Screen)

The primary anticancer assay was performed on a panel of approximately sixty human tumor cell lines derived from nine neoplastic diseases (leukemia, melanoma, lung, colon, CNS, ovarian, renal, prostate, and breast cancers), in accordance with the protocol of the National Cancer Institute Drug Evaluation Branch (Bethesda, MD, USA) [37,38,39,40]. Tested compounds were added to the culture at a single concentration (10^−5^ M) and the cultures were incubated for 48 h. End-point determinations were made with a protein-binding dye, sulforhodamine B (SRB). Results for each tested compound were reported as the percentage of the growth of the treated cells when compared to the untreated control cells. The percentage of the growth was evaluated spectrophotometrically versus controls not treated with test agents. The cytotoxic and/or growth inhibitory effects of the most active selected compounds were tested in vitro against the full panel of human tumor cell lines at concentrations ranging from 10^−4^ to 10^−8^ M. 48-h continuous drug exposure protocol was followed and an SRB protein assay was used to estimate cell viability or growth. Using absorbance measurements [time zero (Tz), control growth in the absence of drug (C), and test growth in the presence of the drug (Ti)], the percentage growth was calculated for each drug concentration. Percentage growth inhibition was calculated as:

[(Ti − Tz)/(C − z)] × 100(1)

for concentrations for which Ti ≥ Tz, [(Ti − Tz)/Tz] × 100 for concentrations for which Ti < Tz. Dose-response parameters (GI_50_, TGI) were calculated for each compound. Growth inhibition of 50% (GI_50_) was calculated from [(Ti − Tz)/(C − Tz)] × 100 = 50, which is the drug concentration resulting in a 50% lower net protein increase in the treated cells (measured by SRB staining) as compared to the net protein increase seen in the control cells. The drug concentration resulting in total growth inhibition (TGI) was calculated from Ti = Tz. Values were calculated for each of these parameters if the level of activity was reached; however, if the effect was not reached or was excessive, the value for that parameter was expressed as more or less than the maximum or minimum concentration tested. The lowest values were obtained with the most sensitive cell lines. Compounds having GI_50_ values ≤ 100 μM were declared to be active. The selectivity index (SI) calculated by dividing the full panel MG___MID (full-panel mean-graph midpoint) (μM) of the compounds **2h** and **2i** by their individual subpanel MG_MID of a cell line (μM) was considered as a measure of compounds’ selectivity. Ratios between 3 and 6 mean moderate selectivity, ratios greater than 6 indicate high selectivity toward the corresponding cell line, while compounds not meeting either of these criteria are rated nonselective [38,55].

### 3.5. Cytotoxicity Study

The cytotoxicity of compounds **2h** and **2i** was analyzed in vitro by the MTT colorimetric assay on human embryonic kidney cell lines (HEK 293) [47]. HEK cell lines were cultured in DMEM supplemented with 10% fetal calf serum and 1% antibiotic-antimycotic mixture culture plates at 37 °C and 5% CO2-humidified incubator. Cells were seeded at a density of 1 × 10^5^ cells/well in 96-well plate for 24 h; then, cells were washed and incubated in fresh medium. Compounds **2h** and **2i** were added at a concentration of 1, 5, 10, 25, and 50 μg/mL to triplicate wells and kept for 24 h, after which cells were washed three times with PBS. After washing, 20 μL of MTT solution (5 mg/mL stock solution) were added to each well, and cells were then incubated for an additional 4 h. The unreacted MTT dye and medium were aspirated off, and 100 μL of DMSO was added to each well to ensure solubilization of formazan crystals. The contents of the plates were mixed for 15 min to achieve complete solubilization of the formazan crystals, and the measurement of optical density was carried out at 570 nm with a micro plate spectrophotometer (MRX Microplate Reader, Dynatech Laboratories Inc., Chantilly, VA, USA) at 570 nm.

## 4. Conclusions

The synthesis of a series of novel 5-aryl(heteryl)idene- and 5-aminomethylidene derivatives of thiazolo[3,2-*b*][1,2,4]triazole-6(5*H*)-one has been achieved and the synthetic pathway for 5-aminomethylidene-thiazolo[3,2-*b*][1,2,4]triazole-6(5*H*)-ones confirmed using X-ray analysis. The stereochemistry and *E/Z*-isomerization of 5-aminomethylidene-thiazolo[3,2-*b*][1,2,4]triazole-6(5*H*)-ones have been studied using ^1^H-, ^13^C- and 2D NMR spectral analysis. The derivatives synthesized have been evaluated for their anticancer activity and it has been possible to identify several hits with anticancer properties and low cytotoxicity to the mammalian cells which should be study further as part of more detailed investigations into this special and especially promising class of heterocyclic compounds. The obtained data contributes to the SAR profile of 5-ene-thiazolo[3,2-*b*][1,2,4]triazole-6(5*H*)-ones for further exploration to design improved and optimized molecules with antitumor activity.

## Figures and Tables

**Table 1:** Hydrogen-bond geometry (A) for **5c**.

*D*—H∙∙∙*A*	*D*—H	H∙∙∙*A*	*D*∙∙∙*A*	*D*—H∙∙∙*A*
N8—H8∙∙∙O12 ^i^	0.85 (2)	1.95 (2)	2.7844 (17)	168 (2)
C11—H11B∙∙∙N3 ^ii^	0.99	2.61	3.603 (2)	179

Symmetry codes: ^i^ 1 − *x*, −1/2 + *y*,1/2 − *z*; ^ii^ 2 − *x*, 1 − *y*, 1 − *z.*

**Table 2:** Anticancer screening data in concentration 10 μM.

Compound	60 Cell Lines Assay in One Dose, 10 μM				
	Mean Growth, %	Range of Growth, %	Most Sensitive Cell Line(s) (Growth Inhibition Percent)/Panel	Positive Cytostatic Effect ^a^	Positive Cytotoxic Effect ^b^
**2a**	89.98	52.79 to 122.91	**NCI-H522** (52.79)/**NSCLC**^#^	0/59	0/59
**2c**	102.57	77.10 to 146.05	NCI-H522 (77.10)/**NSCLC**^#^	0/59	0/59
**2d**	99.67	−73.92 to 147.73	**CCRF-CEM** (-73.92), HL-60 (TB) (−58.44), MOLT-4 (−73.62) all **Leukemia**	2/58	3/58
**2e**	101.82	61.87 to 133.86	SR (61.87)/**Leukemia**	0/58	0/58
**2f**	108.16	86.65 to 261.80	OVCAR-5 (86.65)/**Ovarian Cancer**	0/57	0/57
**2h**	28.98	−48.01 to 121.70	HL-60 (TB) (−32.79), MOLT-4 (−11.15), RPMI-8226 (−16,52), SR (−48.01)/all **Leukemia**; HOP-92 (−21.66)/**NSCLC**^#^; BT-549 (−16.67), HS 578T (−15.94)/all **Breast Cancer**; RXF 393 (−15.19)/**Renal Cancer**; LOX IMVI (−9.62)/**Melanoma**; DU-145 (−21.71)/**Prostate Cancer**; SF-539 (−33.43), SNB-75 (−11.46)/**CNS Cancer**	30/57	12/57
**2i**	61.05	−22.54 to 122.94	CCRF-CEM (−3.49); SR (−25.54) all **Leukemia**	17/58	2/58
**2j**	106.32	82.41 to 135.61	K-562 (82.41)/**Leukemia**	0/56	0/56
**2k**	110.51	25.23 to 180.21	SR (25.23)/**Leukemia**	1/56	0/56
**2l**	105.41	66.88 to 141.12	PC-3 (66.88)/**Prostate Cancer**	0/59	0/59
**3**	97.21	74.85 to 122.06	SR (74.85)/**Leukemia**	0/59	0/59
**5e**	100.59	80.34 to 137.75	T-47D (80.34)/**Breast Cancer**	0/59	0/59
**5f**	93.61	54.41 to 111.61	T-47D (54.41)/**Breast Cancer**	0/59	0/59

NSCLC^#^: Non-Small Cell Lung Cancer. ^a^ Ratio between number of cell lines with percent growth from 0 to 50 and total number of cell lines. ^b^ Ratio between number of cell lines with percent growth of <0 and total number of cell lines.

**Table 3:** Influence of compounds **2h** and **2i** on the growth of tumor panels (GI_50_, TGI, LC_50_) and selectivity index (SI) values.

Panel, MG_MID	GI_50_, μM		SI (GI_50_)		TGI, μM		SI (TGI)		LC_50_, μM		SI (LC_50_)	
	2h	2i	2h	2i	2h	2i	2h	2i	2h	2i	2h	2i
**Leukemia**	1.92	11.32	1.84	0.97	6.85	51.55	1.88	0.55	26.81	99.25	1.39	0.64
Range*	1.35–2.69	2.88–18.19	-	-	3.16– 14.45	9.99– >100	-	-	7.58– 48.97	95.49– >100	-	-
**NSCLC**	6.98	14.13	0.51	0.78	18.22	30.03	0.71	0.94	44.01	64.55	0.84	0.97
Range*	2.29–15.48	10.23–19.49	-	-	9.99– 30.90	25.70– 39.81	-	-	31.62– 60.25	50.11– 83.17	-	-
**Colon cancer**	6.13	12.42	0.58	0.88	17.39	26.51	0.74	1.06	41.39	56.32	0.90	1.12
Range*	2.45–9.33	9.55–16.59	-	-	10.96– 21.37	21.37– 33.11	-	-	33.11– 45.70	46.77– 57.60	-	-
**CNS Cancer**	2.87	9.85	1.23	1.11	10.76	27.42	1.19	1.03	38.58	66.25	0.96	0.95
Range*	1.77–4.07	6.02–13.18	-	-	4.46– 15.48	19.05– 44.66	-	-	12.59– 87.09	43.65– >100	-	-
**Melanoma**	4.79	11.77	0.74	0.93	16.49	25.61	0.78	1.10	41.20	55.37	0.90	1.14
Range*	2.63–8.31	5.62–14.79	-	-	11.22– 20.89	18.19– 33.11	-	-	33.11– 51.28	42.65– 77.62	-	-
**Ovarian Cancer**	8.05	13.69	0.44	0.80	20.34	31.15	0.63	0.90	52.09	68.65	0.71	0.92
Range*	2.11– 15.48	6.60–12.88	-	-	5.62– 30.90	20.89– 38.90	-	-	19.49– 97.72	51.28– >100	-	-
**Renal Cancer**	5.37	13.02	0.66	0.84	16.40	27.13	0.78	1.03	40.46	55.02	0.92	1.15
Range*	1.62– 12.58	5.75–17.37	-	-	8.12– 25.11	21.37– 31.62	-	-	28.84– 50.11	51.28– 58.88	-	-
**Prostate Cancer**	2.67	7.6	1.33	1.44	10.34	27.11	1.25	1.04	30.77	68.15	1.21	0.93
Range*	1.54–3.80	3.98–11.22	-	-	4.46– 16.21	16.21– 38.01	-	-	15.84– 45.70	36.30– >100	-	-
**Breast Cancer**	4.48	9.72	0.79	1.13	15.61	30.29	0.82	0.93	46.05	73.18	0.80	0.86
Range*	<0.01– 15.48	3.54–21.87	-	-	4.07– 41.68	15.84– 67.06	-	-	19.49– >100	41.68– >100	-	-
**Total MG_MID**	3.54	10.96	-	-	12.88	28.18	-	-	37.15	63.09	-	-

Range*—Values range inside panel, μM; GI_50_—molar concentration of the compound that inhibits 50% net cell growth; TGI—molar concentration of the compound leading to the total inhibition; LC_50_ molar concentration of the compound leading to 50% net cell death.

## Data Availability

The data presented in this study are available in this article.

## Supplementary Materials

The following are available online. Table S1: NCI numbers of compouds selected for screening. Table S2: Influence of compounds **2h** and **2i** on the growth of tumor panels, Figures S1–S49: Copies of ^1^H, ^13^C-NMR spectra of compounds **2a**, **2c**, **2d**, **2h**, **2i**, **2l**, **3**, **5a**–**h**, **6b**, **6c**; 2D NMR and LC-MS spectra of compounds **5b**,**c**.

## Sample Availability

Samples of the compounds **2a–l**, **4**, **5a–h, 6a–d** are available from the authors.
