# Supplementary material for: Synthesis, Characterization and In Vitro Evaluation of Novel 5-Ene-thiazolo[3,2-b][1,2,4]triazole-6(5H)-ones as Possible Anticancer Agents
Source: Molecules. 2021 Feb 22;26(4):1162. doi: 10.3390/molecules26041162 (PMC7926352; doi:10.3390/molecules26041162)
Supplement: Supplementary file 1 [file molecules-26-01162-s001.pdf]

Supplementary information

Synthesis, Characterization and In vitro Evaluation of Novel 5-Ene-thiazolo[3,2-*b*][1,2,4]triazole-6(5*H*)-ones as Possible Anticancer Agents

Serhii Holota [1,2,*], Sergiy Komykhov [3,4], Stepan Sysak [1], Andrzej Gzella [5], Andriy Cherkas [6,†] and Roman Lesyk [1,7,*]

1 Department of Pharmaceutical, Organic and Bioorganic Chemistry, Danylo Halytsky Lviv National Medical University, Pekarska 69, 79010 Lviv, Ukraine; stepansysak@pm.me
2 Department of Organic Chemistry and Pharmacy, Lesya Ukrainka Volyn National University, Volya Avenue 13, 43025 Lutsk, Ukraine
3 State Scientific Institution "Institute for Single Crystals", National Academy of Sciences of Ukraine, Nauky Ave 60, Kharkiv 61072, Ukraine
4 Applied Chemistry Department, Karazin Kharkiv National University, Svobody Sq. 4, Kharkiv 61022, Ukraine
5 Department of Organic Chemistry, Poznan University of Medical Sciences, Grunwaldzka 6, 60-780 Poznan, Poland; akgzella@ump.edu.pl
6 Department of Internal Medicine #1, Danylo Halytsky Lviv National Medical University, Pekarska 69, 79010 Lviv, Ukraine; cherkasandriy@yahoo.com
7 Department of Public Health, Dietetics and Lifestyle Disorders, Faculty of Medicine, University of Information Technology and Management in Rzeszow, Sucharskiego 2, 35-225 Rzeszow, Poland
* Correspondence: golota_serg@yahoo.com (S.H.); dr_r_lesyk@org.lviv.net (R.L.). Tel.: +380-97-226-00-66 (S.H.); +380-32-275-59-66 (R.L.)
† Present address: Team Early Projects Type 1 Diabetes, Therapeutic Area Diabetes and Cardiovascular Medicine, Research & Development, Sanofi-Aventis Deutschland GmbH., Industriepark Höchst-H831, Frankfurt am Main 65926, Germany; andriy.cherkas@sanofi.com

Supplementary data: Anticancer activity of compounds **2h** and **2i**. The ^{1}H,^{13}C NMR spectra of compounds **2a, 2c, 2d**, **2h, 2i, 2l, 3**, **5a-h, 6b, 6c**; 2D NMR and LC-MS spectra of compounds **5b,c**.

Table of Contents

NCI numbers of compounds selected for DTP 60 lines screening ……………S2

Influence of compounds **2h** and **2i** on the growth of tumor panels…………S3-4

NOESY spectrum of **5c** ………………………………………………………… S6

X-ray of **5c** …………………………………………………………………S7

Copies of ^{1}H, ^{13}C NMR spectra of compounds **2a, 2c, 2d**, **2h, 2i, 2l, 3**, **5a-h, 6b, 6c**; 2D NMR and LC-MS spectra of compounds **5b,c** …………………….. S8-33

Table S1. NCI numbers of compouds selected for screening

Compound	NCI Number
**2a**	NSC D-789593/1
**2c**	NSC D-789587/1
**2d**	NSC 741984/1
**2e**	NSC 741996/1
**2f**	NSC 742002/1
**2h**	NSC 741966/1
**2i**	NSC 741973/1
**2j**	NSC 741974/1
**2k**	NSC 741975/1
**2l**	NSC 741962/1
**3**	NSC 741968/1
**5e**	NSC D-789611/1
**5f**	NSC D-789595/1

Table S2. Influence of compounds **2h** and **2i** on the growth of tumor panels

Panel	Cell lines	GI_{50}, μM		SI (GI_{50})		TGI, μM		SI (TGI)		LC_{50}, μM		SI (LC_{50})	
		**4h**	**4i**	**4h**	**4i**	**4h**	**4i**	**4h**	**4i**	**4h**	**4i**	**4h**	**4i**
Leukemia	CCRF-CEM	1.35	2.88	2.62	3.81	3.16	9.99	4.08	2.82	7.58	>100	4.90	-
	HL-60 (TB)	2.69	12.30	1.32	0.89	9.54	41.68	1.35	0.68	37.11	>100	1.00	-
	K-562	2.51	18.19	1.41	0.60	14.45	>100	0.92	-	48.97	>100	0.76	-
	MOLT-4	1.90	18.19	1.86	0.60	5.01	52.48	2.57	0.54	22.90	>100	1.62	-
	RPMI-8226	1.69	5.37	2.09	2.04	4.89	25.70	2.63	1.09	26.91	95.49	1.38	0.66
	SR	1.38	10.96	2.56	1.00	4.07	79.43	3.16	0.35	17.37	>100	2.13	-
	**MG_MID**	**1.92**	**11.32**	**1.84**	**0.97**	**6.85**	**51.55**	**1.88**	**0.55**	**26.81**	**99.25**	**1.39**	**0.64**
Non-Small Cell Lung Cancer	A549/ATCC	15.48	19.49	0.23	0.56	30.90	39.81	0.42	0.71	60.25	79.43	0.62	0.79
	EKVX	2.51	12.88	1.41	0.85	9.99	25.70	1.29	1.09	31.62	50.11	1.17	1.26
	HOP-62	4.67	13.18	0.76	0.83	16.59	26.91	0.78	1.05	42.65	53.70	0.87	1.17
	HOP-92	2.29	10.23	1.55	1.07	11.74	29.51	1.09	0.95	34.67	83.17	1.07	0.76
	NCI-H226	3.09	16.98	1.15	0.65	17.37	33.11	0.74	0.85	44.66	67.60	0.83	0.93
	NCI-H322M	13.18	15.13	0.27	0.72	25.70	28.84	0.50	0.97	51.28	53.70	0.72	1.17
	NCI-H460	11.48	13.48	0.31	0.81	23.44	27.54	0.55	1.02	48.97	56.23	0.76	1.12
	NCI-H522	3.16	11.74	1.12	0.93	9.99	28.84	1.29	0.97	38.01	72.44	0.98	0.87
	**MG_MID**	**6.98**	**14.13**	**0.51**	**0.78**	**18.22**	**30.03**	**0.71**	**0.94**	**44.01**	**64.55**	**0.84**	**0.97**
Colon cancer	COLO 205	7.58	14.79	0.47	0.74	19.95	31.62	0.65	0.89	44.66	67.60	0.83	0.93
	HCC-2998	7.07	16.59	0.50	0.66	18.62	33.11	0.69	0.85	43.65	66.06	0.85	0.96
	HCT-116	7.58	9.55	0.47	1.15	20.89	23.44	0.62	1.20	45.70	54.95	0.81	1.15
	HCT-15	9.33	13.80	0.38	0.79	21.37	27.54	0.60	1.02	45.70	54.95	0.81	1.15
	HT29	4.67	12.02	0.76	0.91	15.13	24.54	0.85	1.15	38.90	48.97	0.95	1.29
	KM12	2.45	10.23	1.44	1.07	10.96	23.98	1.18	1.17	33.11	54.95	1.12	1.15
	SW-620	4.26	9.99	0.83	1.10	14.79	21.37	0.87	1.32	38.01	46.77	0.97	1.35
	**MG_MID**	**6.13**	**12.42**	**0.58**	**0.88**	**17.39**	**26.51**	**0.74**	**1.06**	**41.39**	**56.32**	**0.90**	**1.12**
CNS Cancer	SF-268	2.39	6.02	1.48	1.82	10.47	28.84	1.23	0.97	37.15	>100	1.00	-
	SF-295	1.86	10.47	1.90	1.05	5.24	23.44	2.46	1.20	18.62	51.28	1.99	1.23
	SF-539	1.77	12.58	2.00	0.87	4.46	25.11	2.88	1.12	12.59	50.11	2.95	1.26
	SNB-19	3.80	10.23	0.93	1.07	14.45	23.44	0.89	1.20	38.01	52.48	0.97	1.20
	SNB-75	3.38	13.18	1.04	0.83	15.48	44.66	0.83	0.63	87.09	>100	0.43	-
	U251	4.07	6.60	0.87	1.66	14.45	19.05	0.89	1.48	38.01	43.65	0.97	1.45
	**MG_MID**	**2.87**	**9.85**	**1.23**	**1.11**	**10.76**	**27.42**	**1.19**	**1.03**	**38.58**	**66.25**	**0.96**	**0.95**
Melanoma	LOX IMVI	2.63	5.62	1.35	1.95	11.22	18.19	1.15	1.55	33.11	42.65	1.12	1.48
	MALME-3M	7.94	14.79	0.45	0.74	20.89	28.18	0.62	1.00	45.70	53.70	0.81	1.17
	M14	8.31	12.58	0.43	0.87	20.89	27.54	0.62	1.02	45.70	58.88	0.81	1.07
	SK-MEL-2	3.98	14.45	0.89	0.75	13.80	33.11	0.93	0.85	38.90	77.62	0.95	0.81
	SK-MEL-28	6.16	12.02	0.57	0.91	20.41	23.98	0.63	1.18	51.28	48.97	0.72	1.29
	SK-MEL-5	3.23	10.47	1.09	1.05	14.79	21.87	0.87	1.29	38.01	46.77	0.97	1.35
	UACC-257	3.38	10.47	1.05	1.05	15.13	25.70	0.85	1.09	38.90	63.09	0.95	1.00
	UACC-62	2.69	13.80	1.32	0.79	14.79	26.30	0.87	1.07	38.01	51.28	0.97	1.23
	**MG_MID**	**4.79**	**11.77**	**0.74**	**0.93**	**16.49**	**25.61**	**0.78**	**1.10**	**41.20**	**55.37**	**0.90**	**1.14**
Ovarian Cancer	IGROV1	-	6.60	-	1.66	-	20.89	-	1.35	-	51.28	-	1.23
	OVCAR-3	2.18	13.80	1.62	0.79	5.62	27.54	2.29	1.02	19.49	54.95	1.90	1.15
	OVCAR-4	8.91	12.88	0.40	0.85	30.90	38.90	0.42	0.72	97.72	>100	0.38	-
	OVCAR-5	11.22	15.48	0.32	0.71	23.44	30.19	0.55	0.93	47.86	58.88	0.77	1.07
	OVCAR-8	3.16	13.48	1.12	0.81	12.88	33.88	1.00	0.83	41.68	85.11	0.89	0.74
	SK-OV-3	15.48	19.95	0.29	0.55	28.84	35.48	0.45	0.79	53.70	61.65	0.69	1.02
	**MG_MID**	**8.05**	**13.69**	**0.44**	**0.80**	**20.34**	**31.15**	**0.63**	**0.90**	**52.09**	**68.65**	**0.71**	**0.92**
Renal Cancer	786-0	4.16	11.22	0.85	0.98	15.84	23.98	0.81	1.17	39.81	51.28	0.93	1.23
	A498	10.47	17.37	0.34	0.63	22.38	31.62	0.58	0.89	46.77	56.23	0.79	1.12
	ACHN	12.58	15.48	0.28	0.71	25.11	28.84	0.51	0.97	50.11	53.70	0.74	1.17
	CAKI-1	2.39	10.47	1.48	1.05	8.12	23.98	1.58	1.17	28.84	54.95	1.28	1.15
	RXF 393	1.90	5.75	1.86	1.91	8.51	21.37	1.51	1.31	36.30	58.88	1.02	1.07
	TK-10	7.58	16.21	0.47	0.67	20.41	29.51	0.63	0.95	35.48	51.28	1.05	1.23
	UO-31	2.29	15.13	1.55	0.72	17.37	29.51	0.74	0.95	44.66	54.95	0.83	1.15
	SN12C	1.62	12.58	2.18	0.87	12.88	25.11	1.00	1.12	41.68	58.88	0.89	1.07
	**MG_MID**	**5.37**	**13.02**	**0.66**	**0.84**	**16.40**	**27.13**	**0.78**	**1.03**	**40.46**	**55.02**	**0.92**	**1.15**
Prostate Cancer	PC-3	3.80	11.22	0.93	0.98	16.21	38.01	0.79	0.74	45.70	>100	0.81	-
	DU-145	1.54	3.98	2.30	2.75	4.46	16.21	2.89	1.74	15.84	36.30	2.34	1.74

	**MG_MID**	**2.67**	**7.60**	**1.33**	**1.44**	**10.34**	**27.11**	**1.25**	**1.04**	**30.77**	**68.15**	**1.21**	**0.93**
Breast Cancer	MCF7	0.79	5.12	4.48	2.14	4.07	19.05	3.16	1.48	19.49	50.11	1.90	1.26
	NCI/ADR-RES	15.48	21.87	0.29	0.50	41.68	67.60	0.31	0.42	>100	>100	-	-
	HS 578T	2.04	3.54	1.74	3.01	5.24	19.49	2.46	1.45	33.88	60.25	1.10	1.05
	MDA-MB-435	2.95	10.47	1.20	1.05	12.58	25.11	1.02	1.12	28.84	>100	1.29	-
	BT-549	2.34	3.80	1.51	2.88	8.51	15.84	1.51	1.78	35.48	60.25	1.05	1.05
	T-47D	7.76	10.96	0.46	1.00	25.70	38.01	0.50	0.74	28.84	41.68	1.29	1.51
	MDA-MB-231/ATCC	<0.01	12.30	-	0.89	11.48	26.91	1.12	1.05	75.85	>100	0.49	-
	**MG_MID**	**4.48**	**9.72**	**0.79**	**1.13**	**15.61**	**30.29**	**0.82**	**0.93**	**46.05**	**73.18**	**0.80**	**0.86**
**MG_MID**		**3.54**	**10.96**			**12.88**	**28.18**			**37.15**	**63.09**		

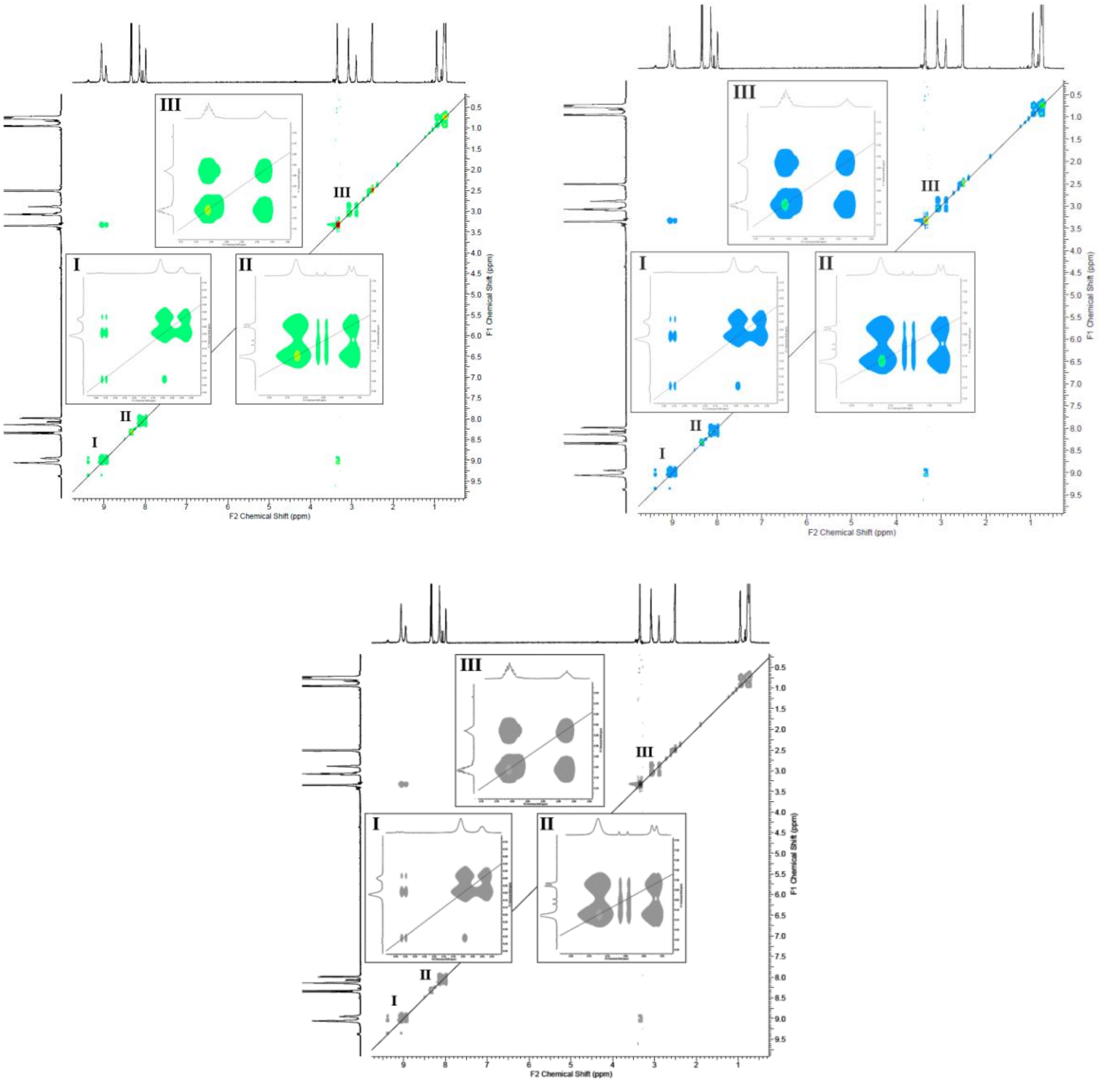

Figure S1. NOESY spectrum of **5c**.

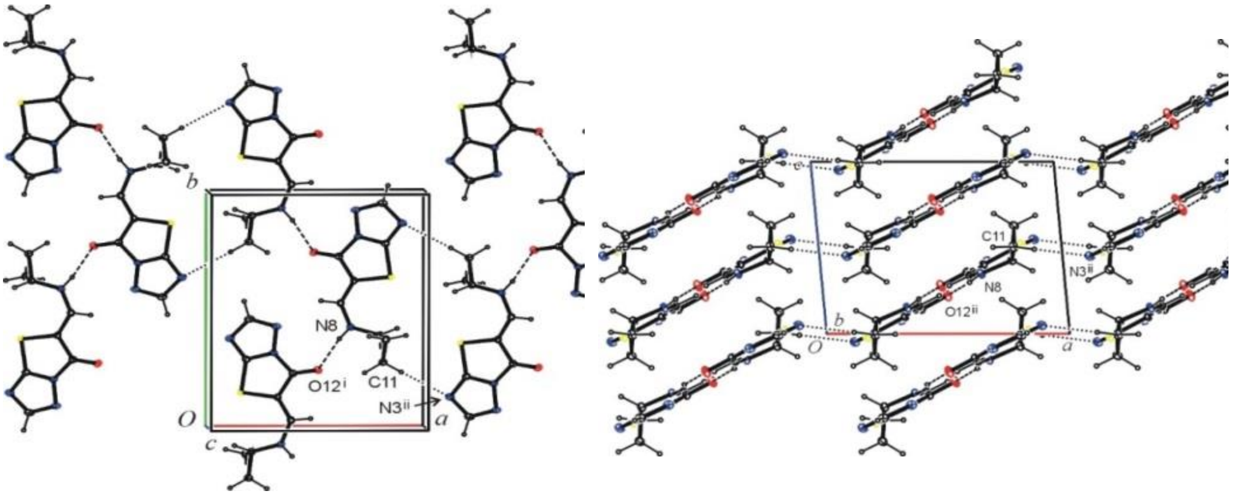

(a) **(b)**

Figure S2. Part of molecular packing in the crystal **5c**, showing (**a**) molecules linked by N8-H8…O12^{i} and C11-H11a…N3^{ii} hydrogen bonds into layers and (**b**) the hydrogen bonded layers parallel to the (-102).

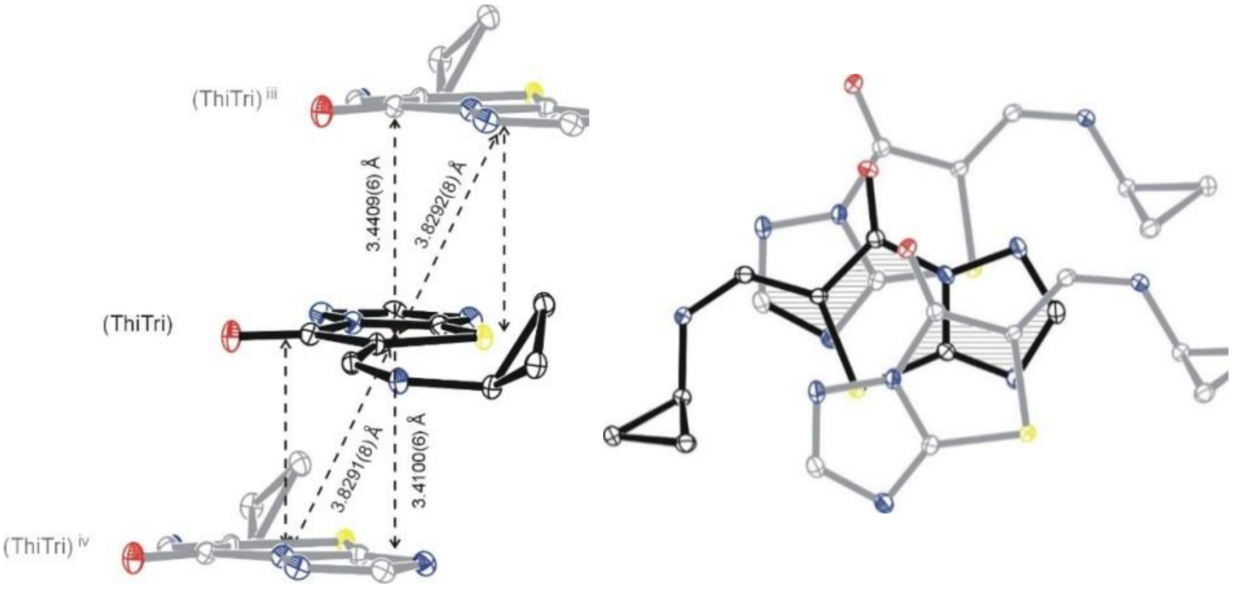

(**a**) (**b**)

Figure S3. $\pi \cdots \pi$ Interactions between thiazolo[3,2-*b*][1,2,4]triazol-6(5*H*)-one systems. The molecules are shown in two orthogonal projections. Hydrogen atoms have been omitted for clarity.

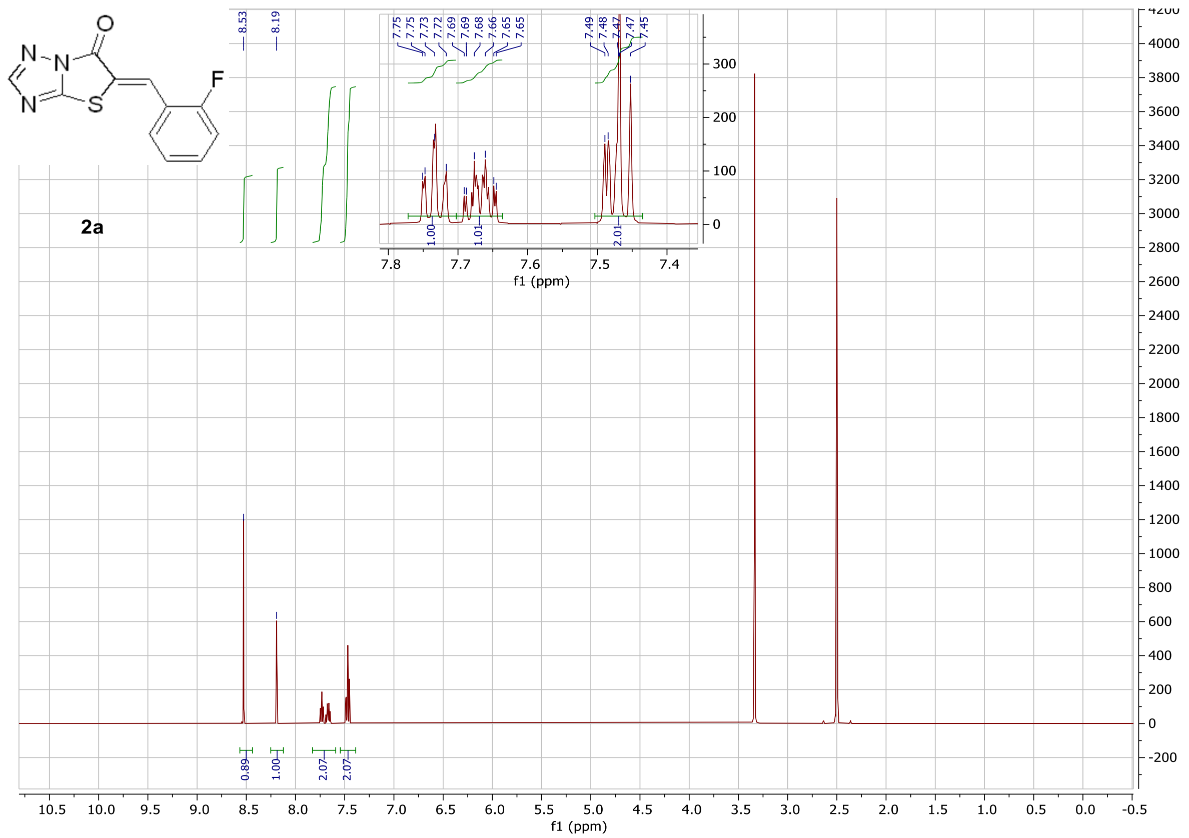

Figure S4. ^{1}H NMR spectrum **2a**

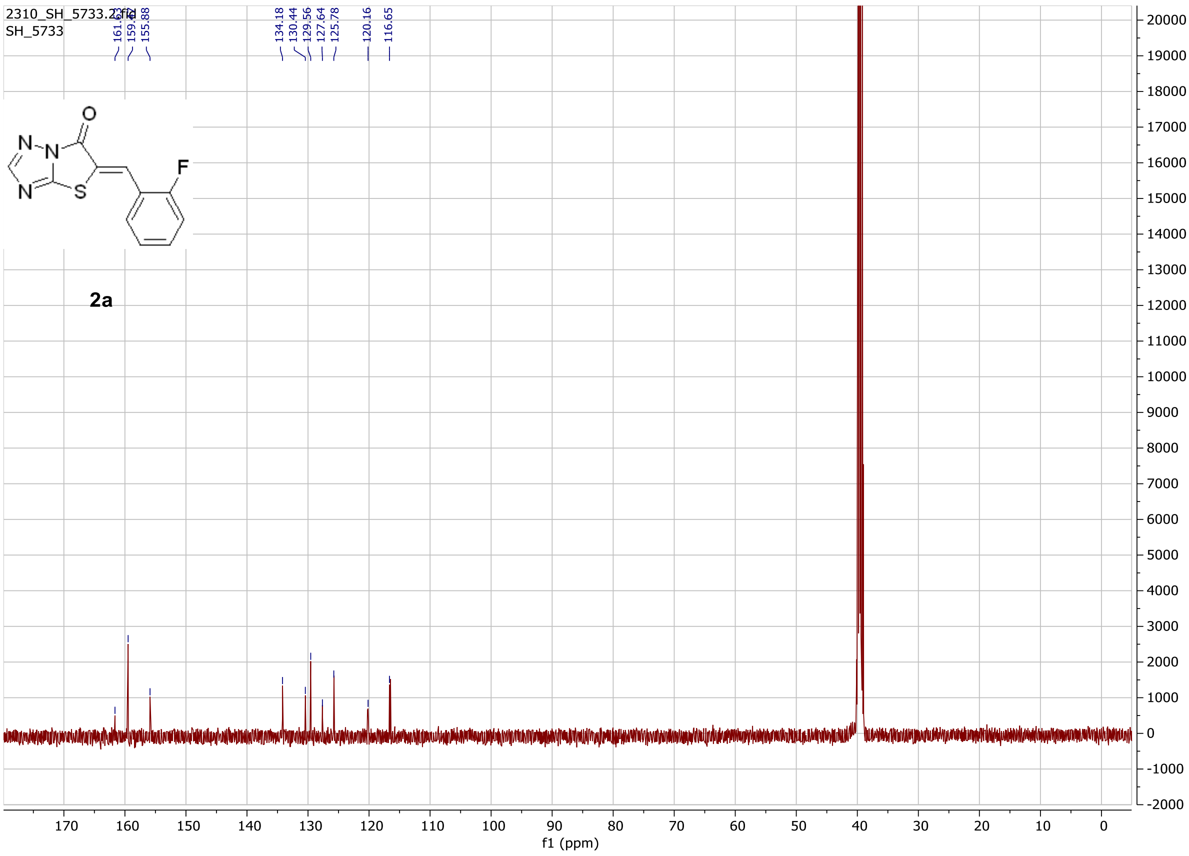

Figure S5. ^{13}C NMR spectrum **2a**

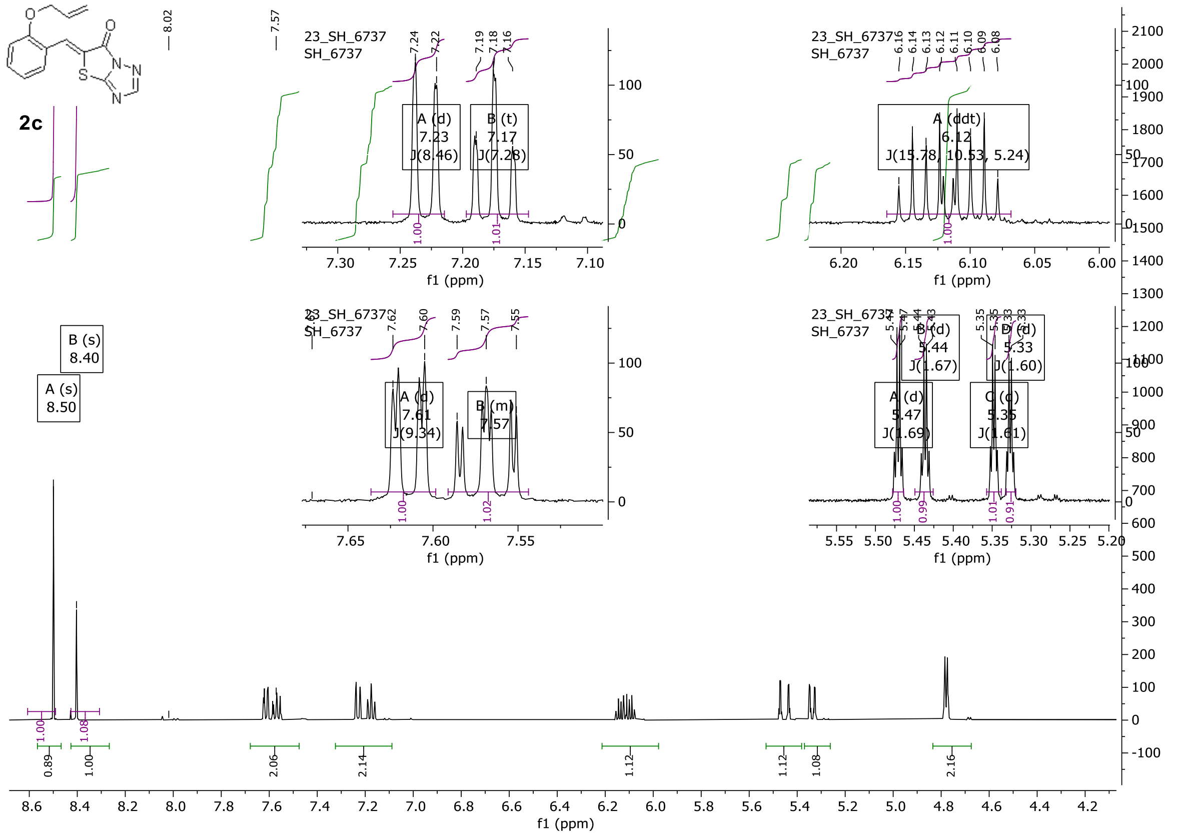

Figure S6. ^{1}H NMR spectrum **2c**.

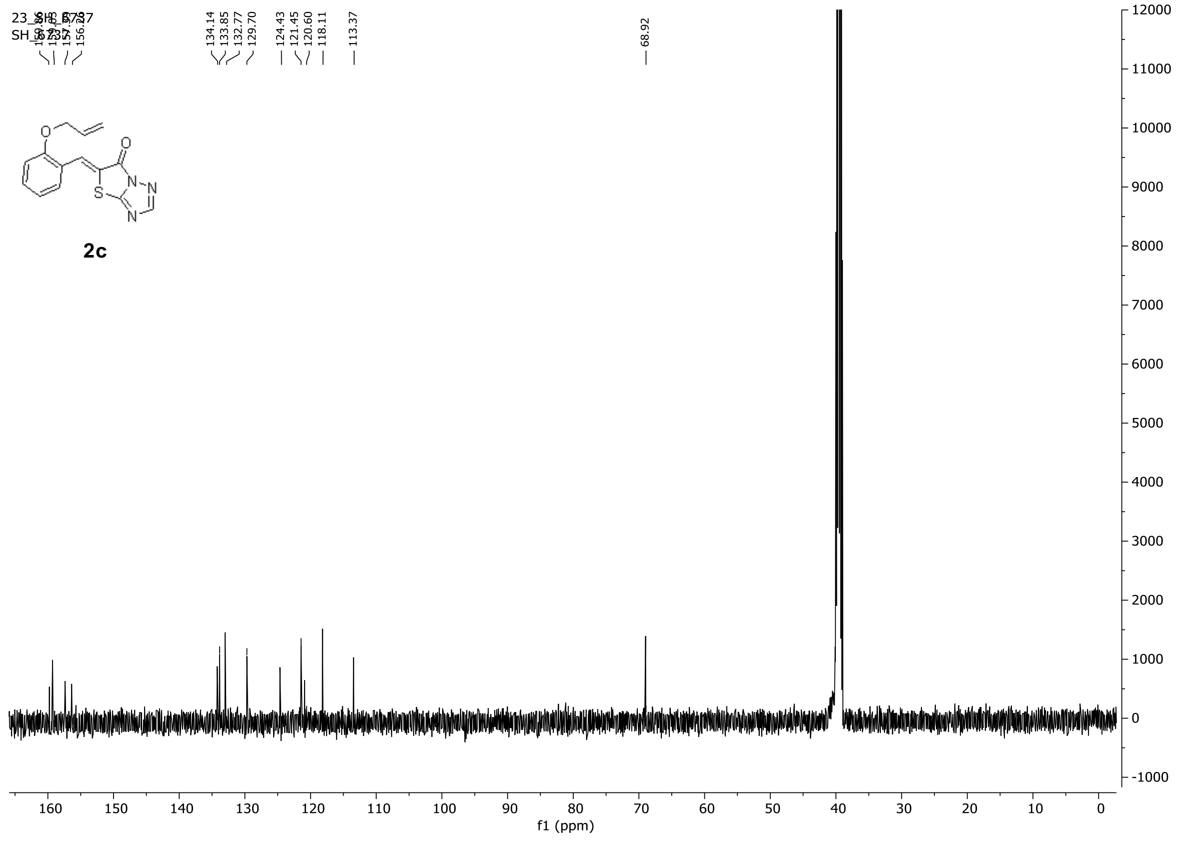

Figure S7. ^{13}C NMR spectrum **2c**

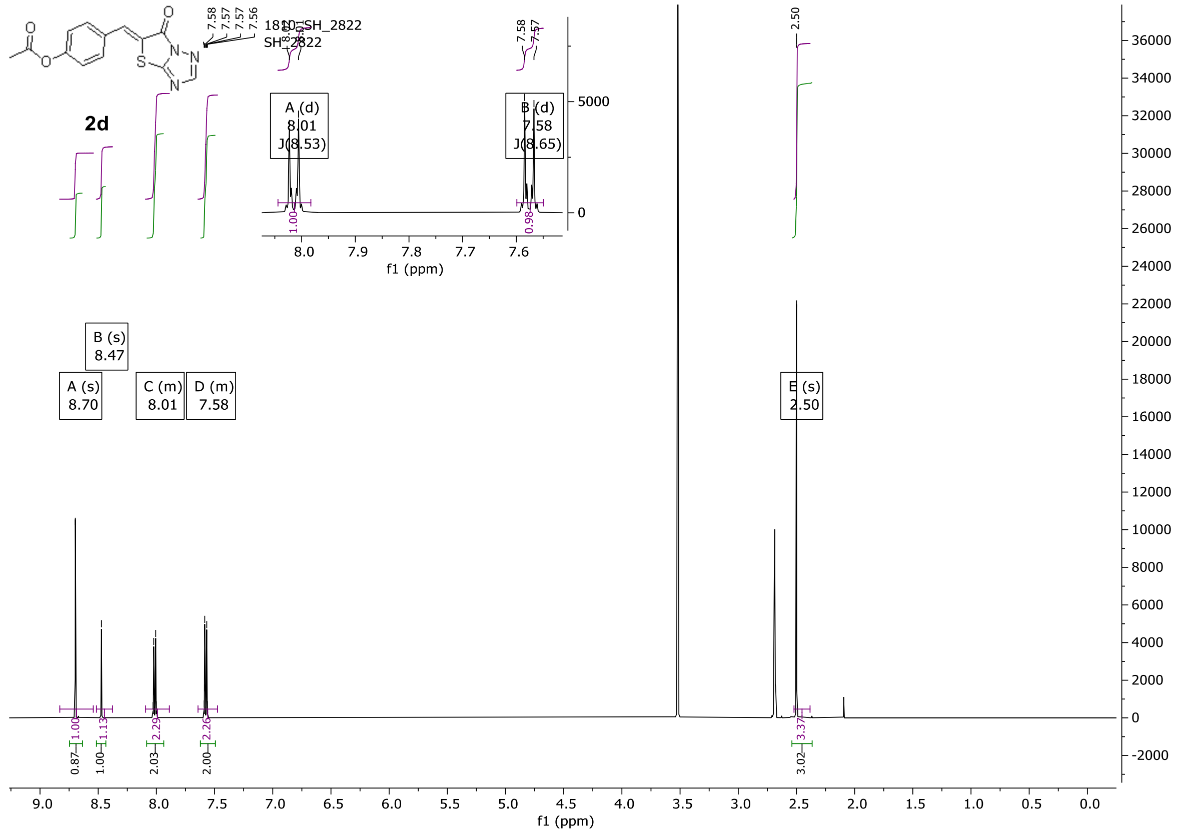

Figure S8. ^{1}H NMR spectrum **2d**

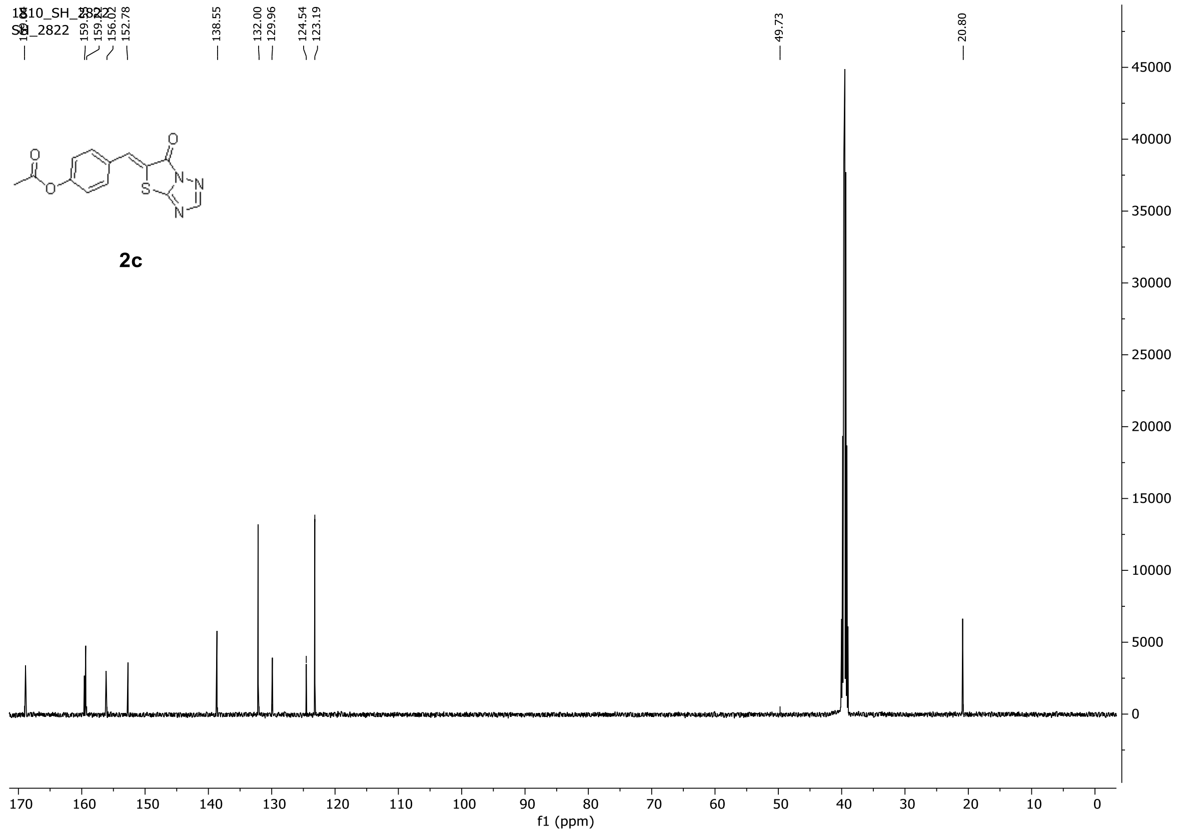

Figure S9. ^{13}C NMR spectrum **2d**

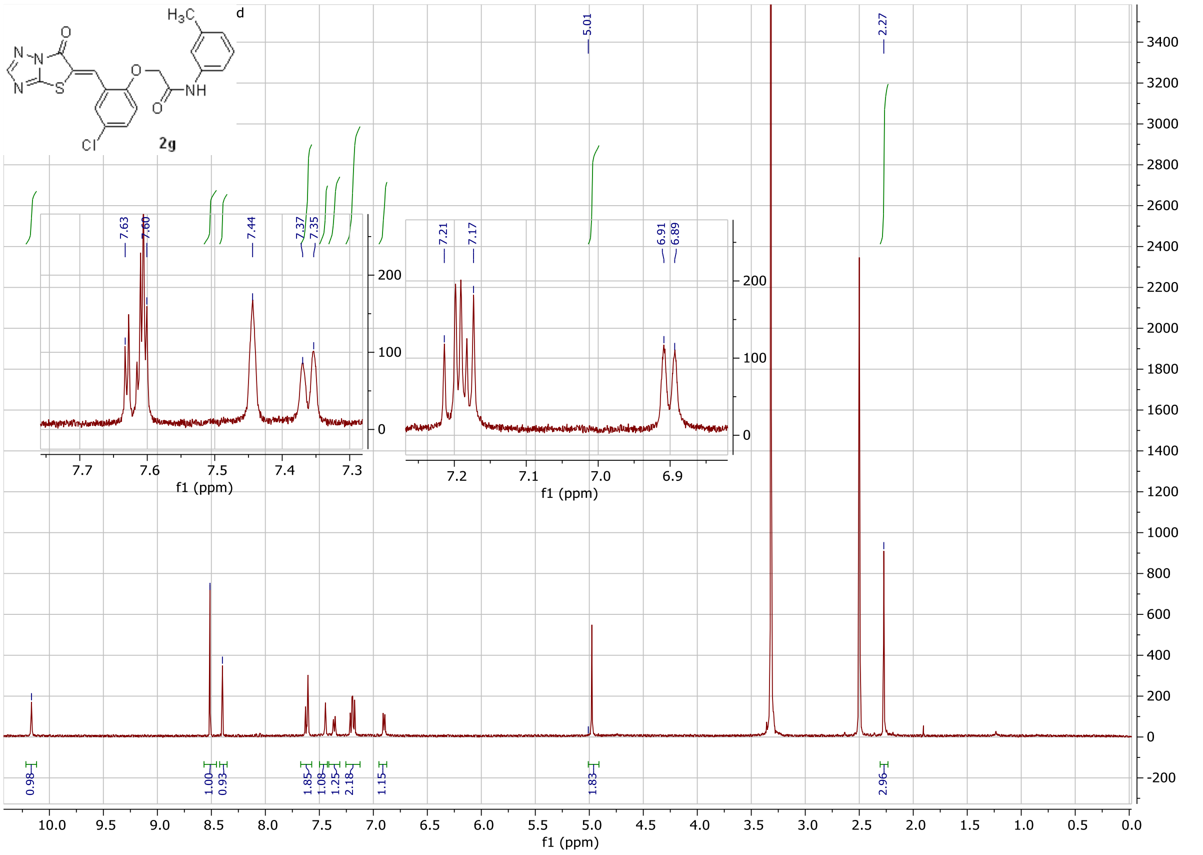

Figure S10. ^{1}H NMR spectrum **2g**

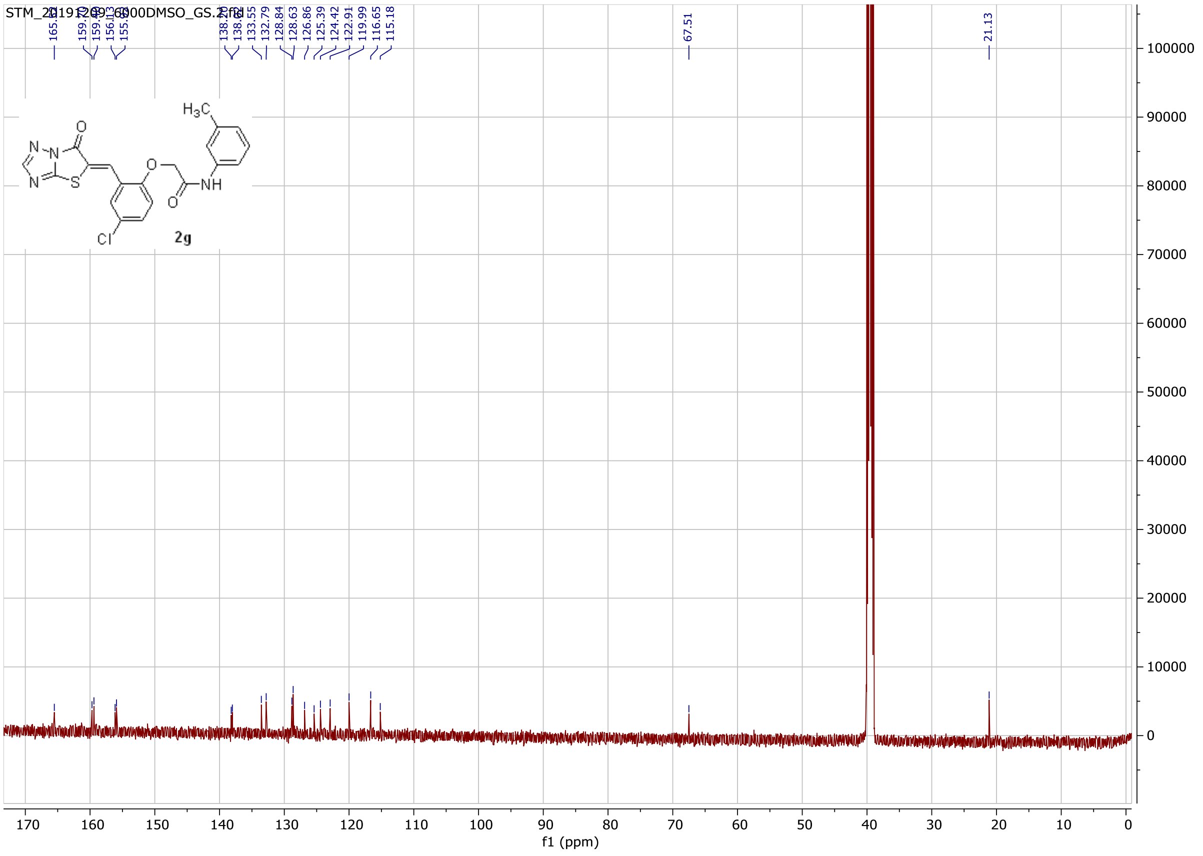

Figure S11. ^{13}C NMR spectrum **2g**

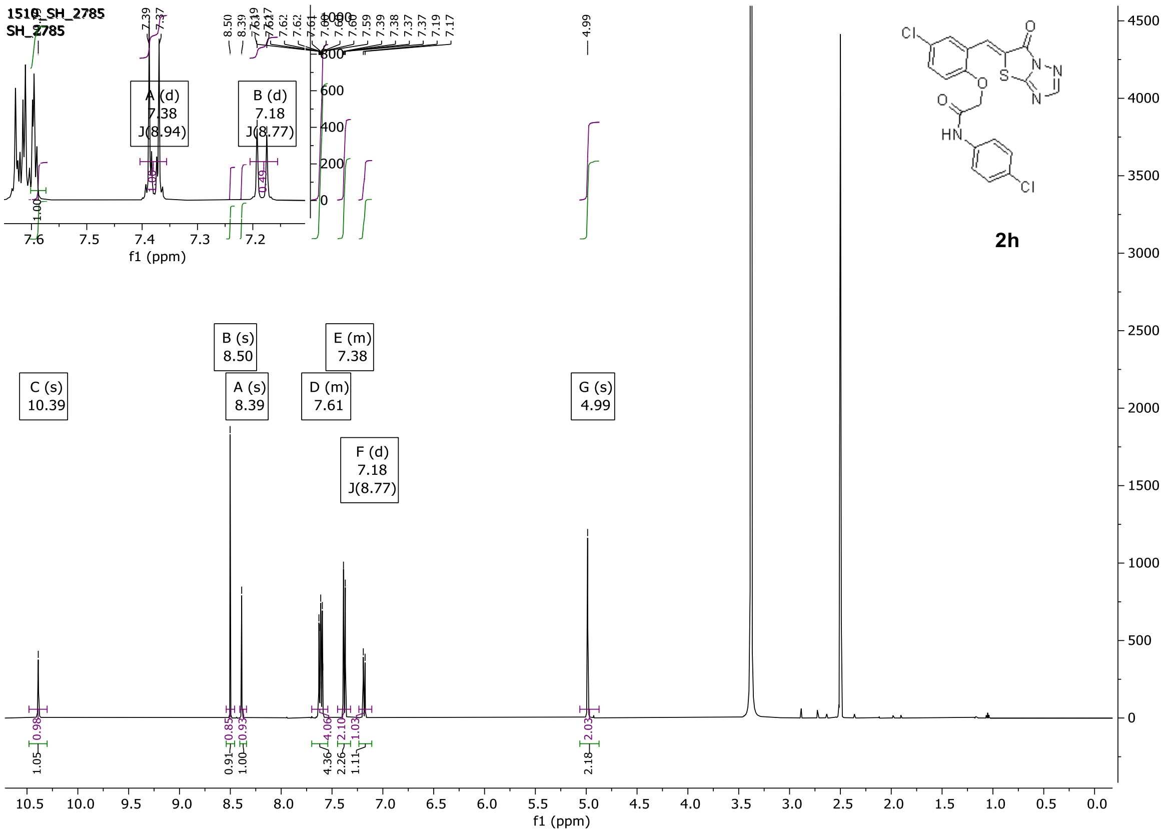

Figure S12. ^{1}H NMR spectrum **2h**

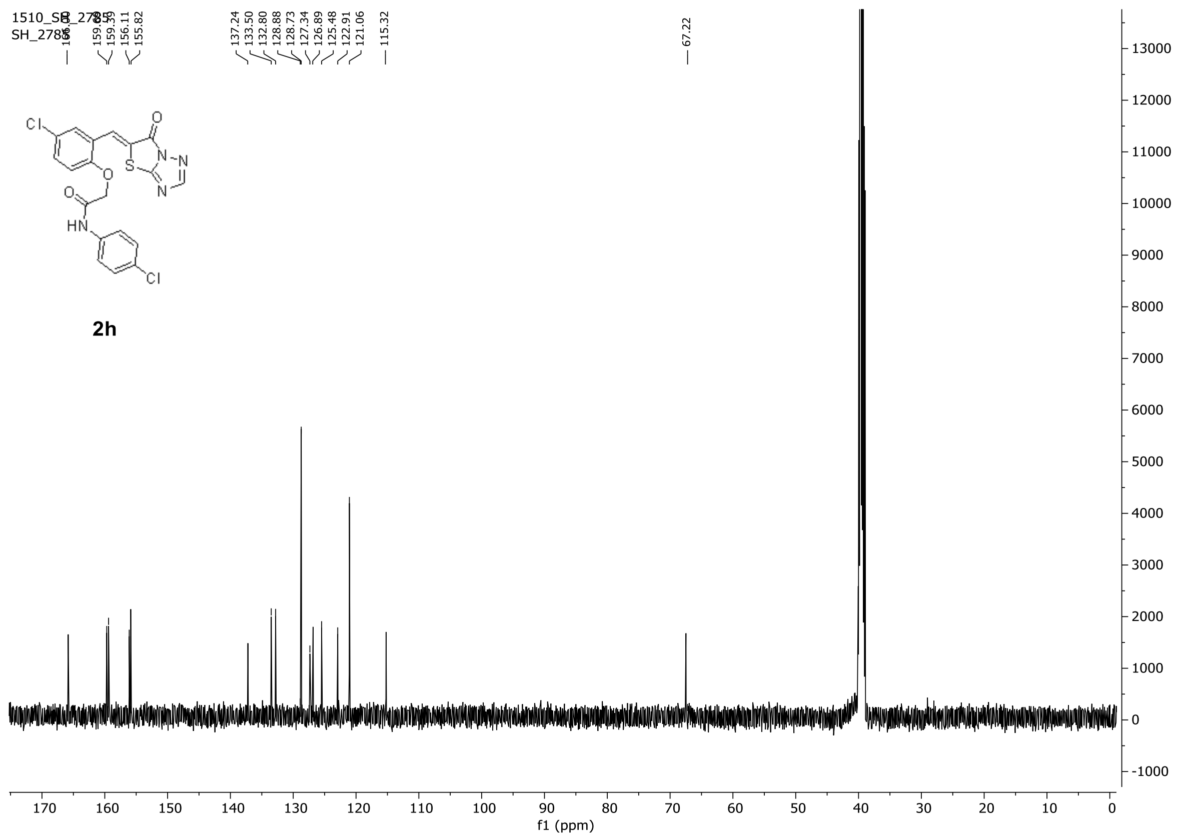

Figure S13. ^{13}C NMR spectrum **2h**

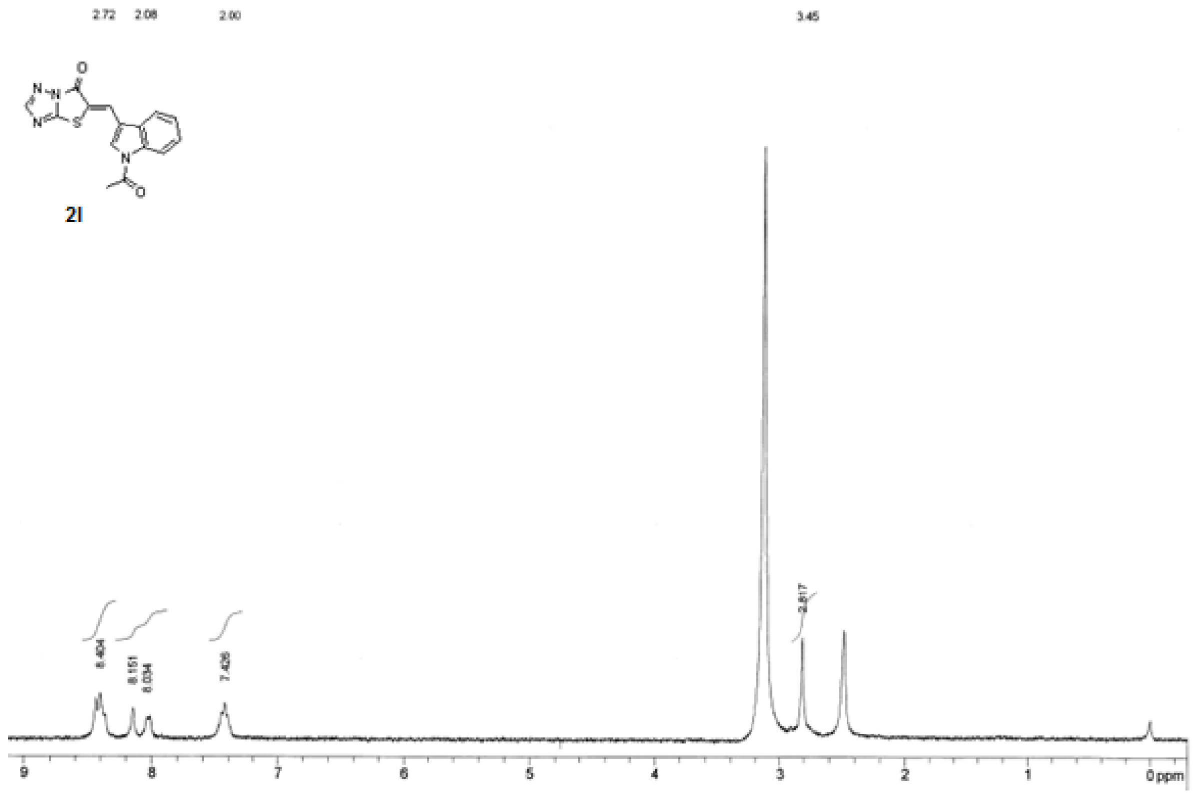

Figure S14. ^{1}H NMR spectrum **2l**

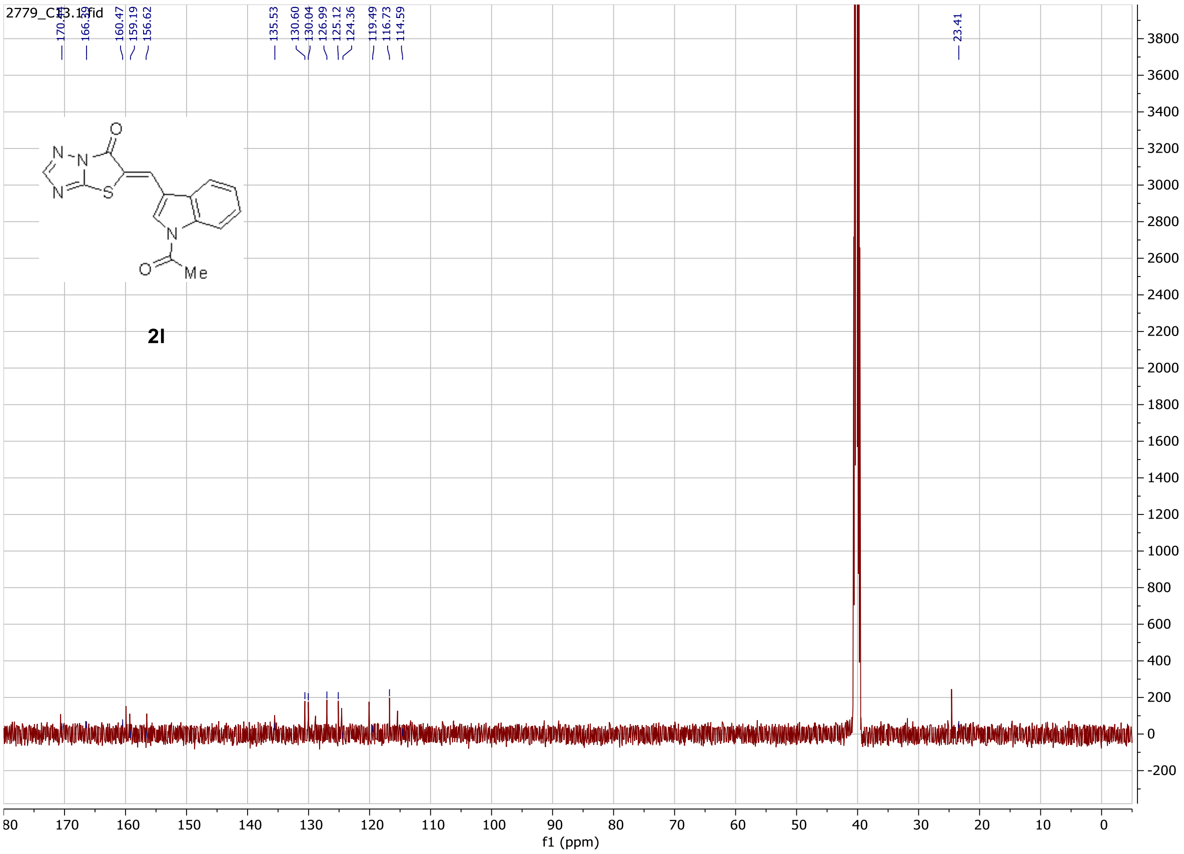

Figure S15. ^{13}C NMR spectrum **2l**

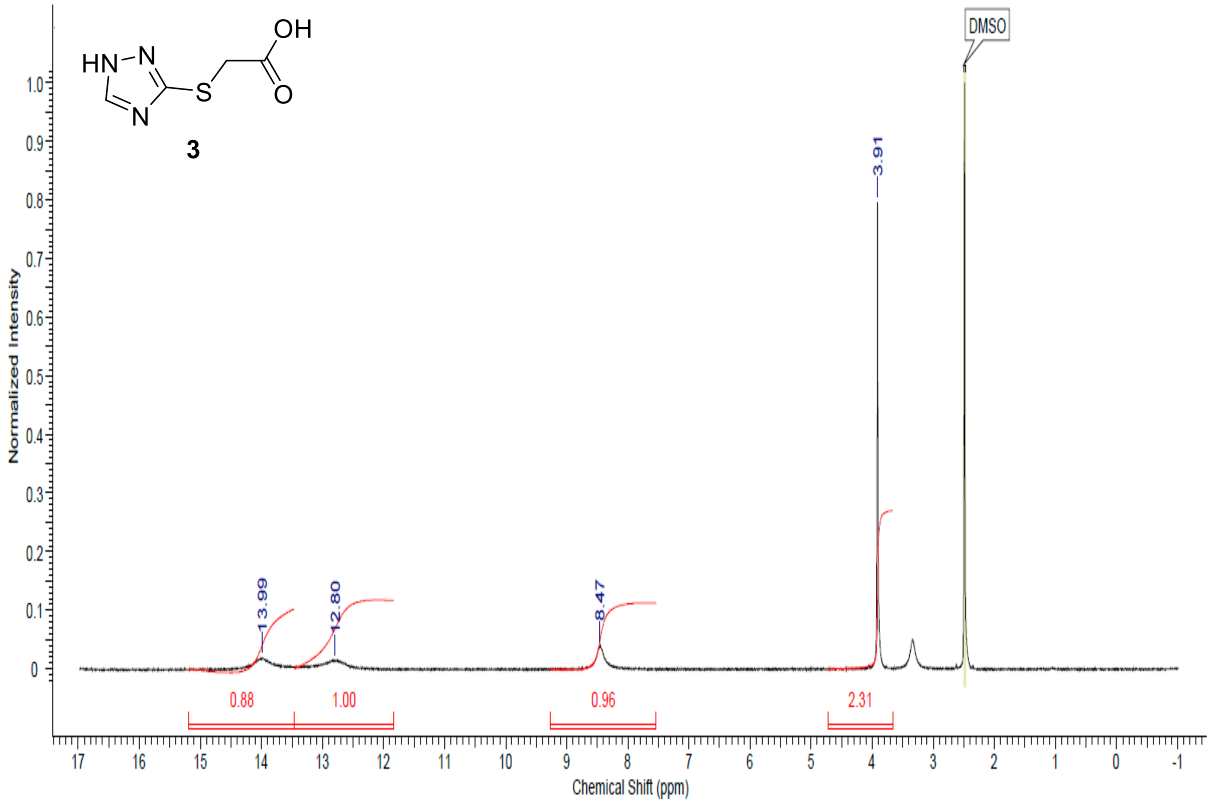

Figure S16. ^{1}H NMR spectrum **3**

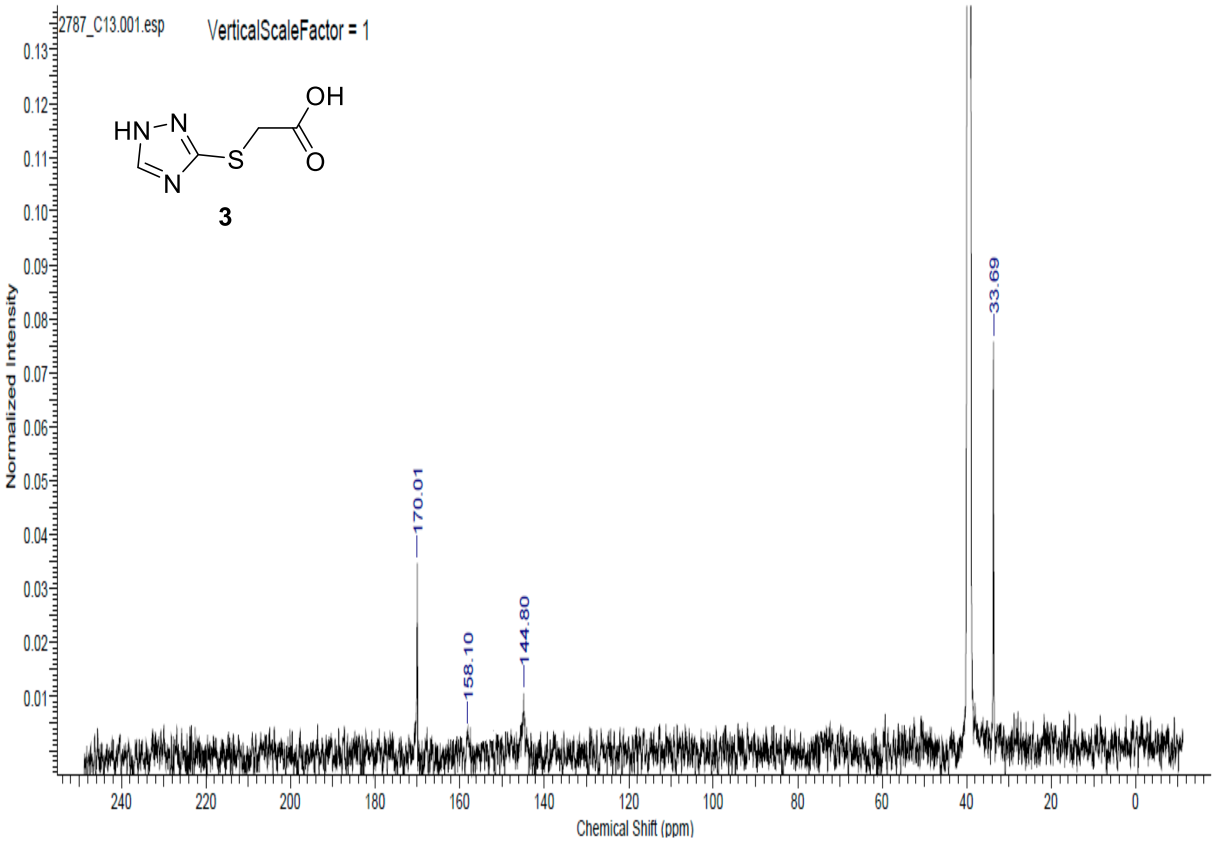

Figure S17. ^{13}C NMR spectrum **3**

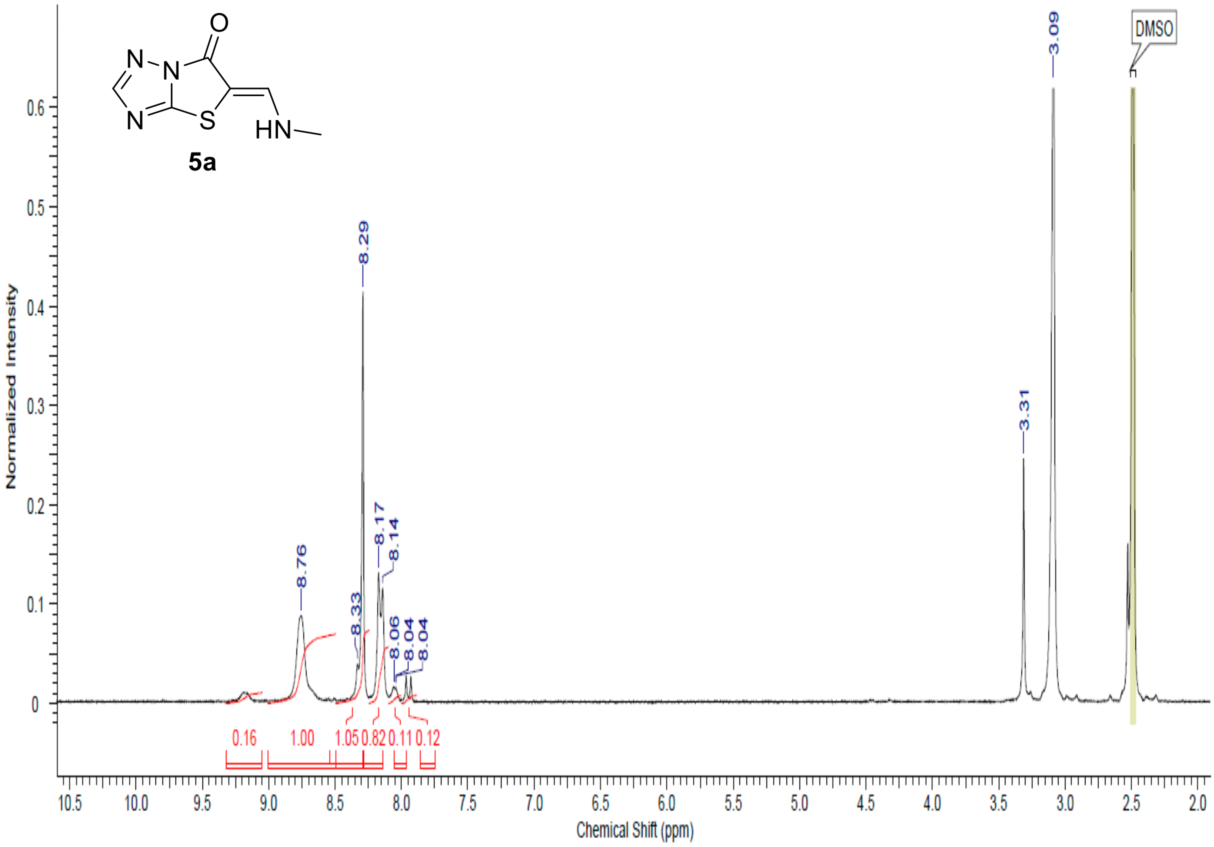

Figure S18. ^{1}H NMR spectrum **5a**

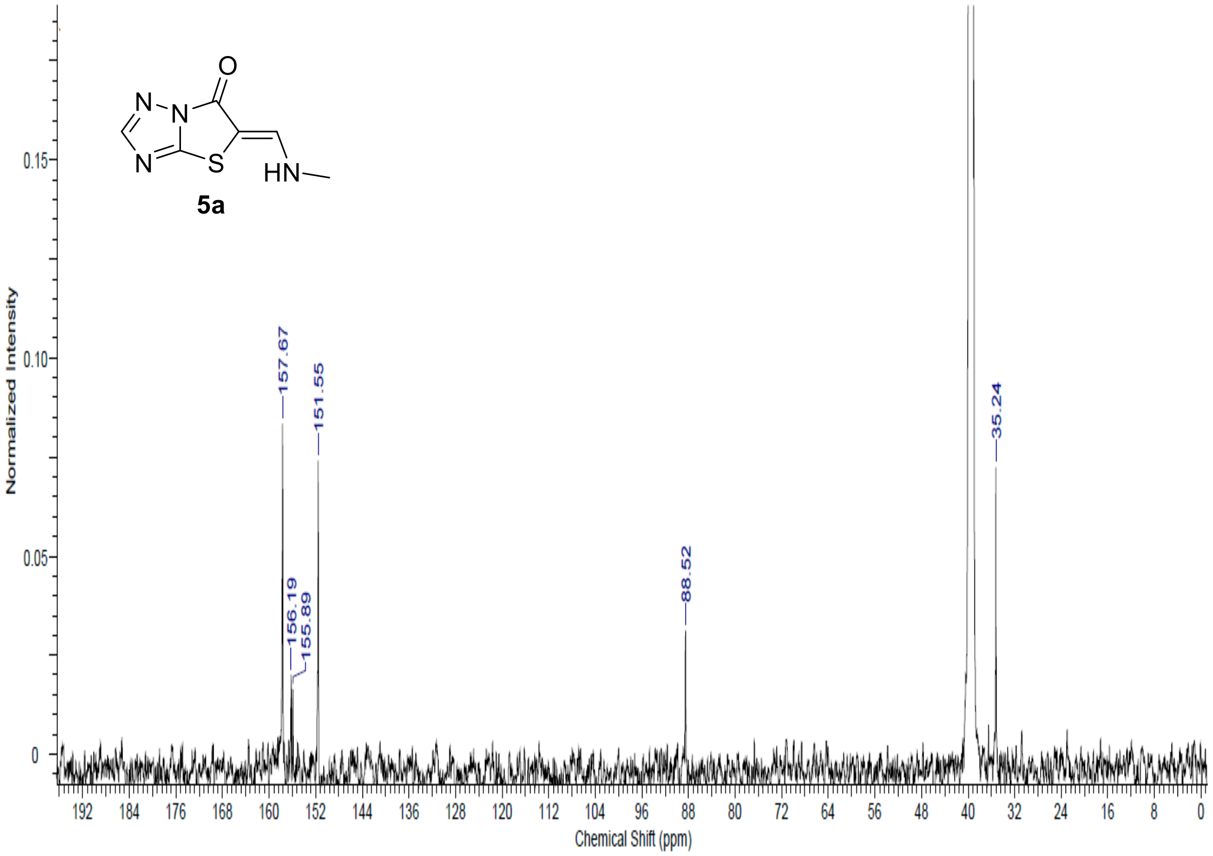

Figure S19. ^{13}C NMR spectrum **5a**

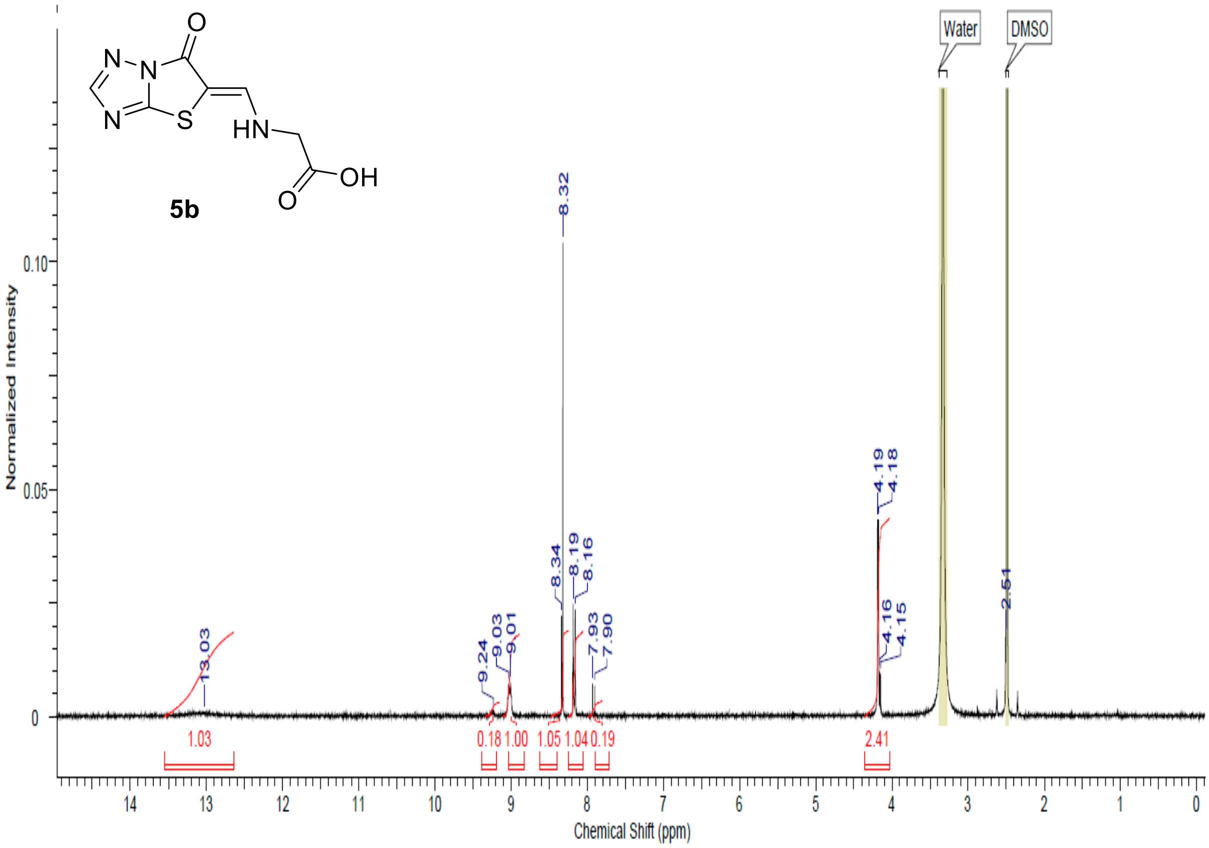

Figure S20. ^{1}H NMR spectrum **5b**

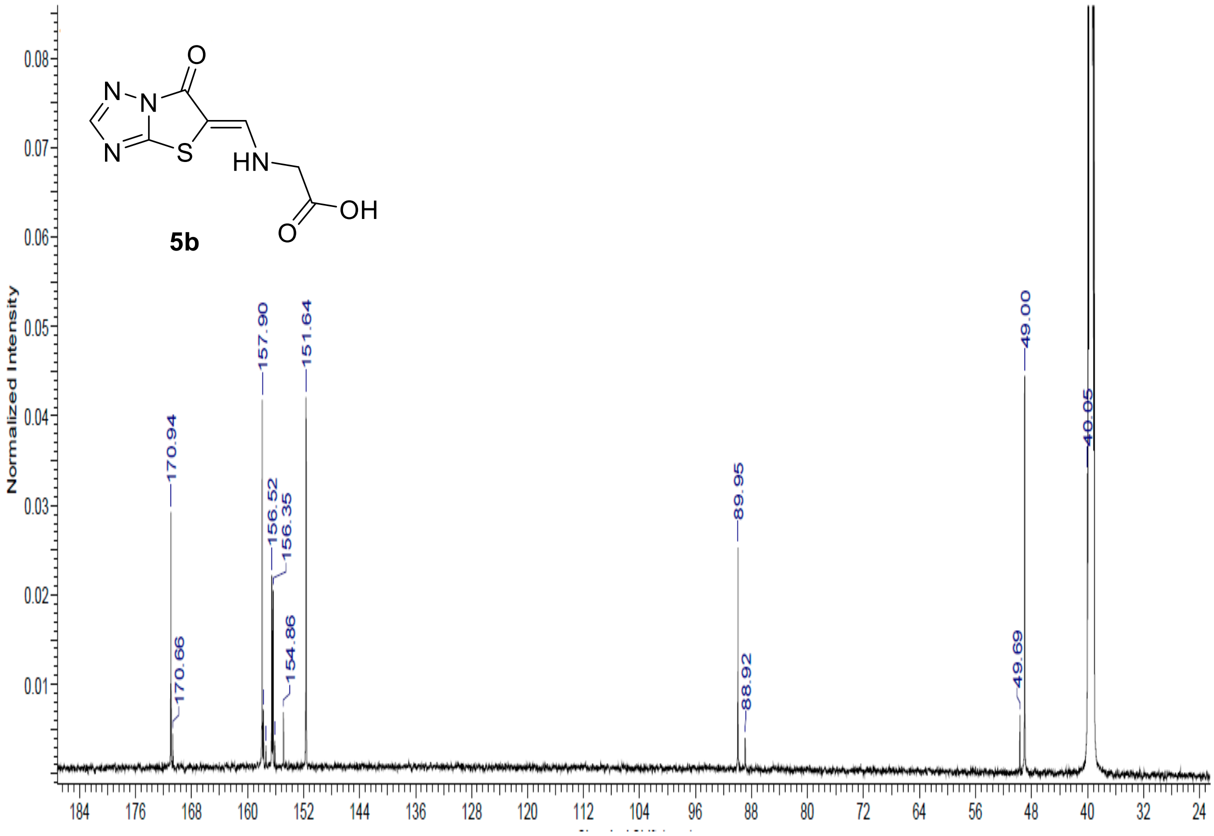

Figure S21. ^{13}C NMR spectrum **5b**

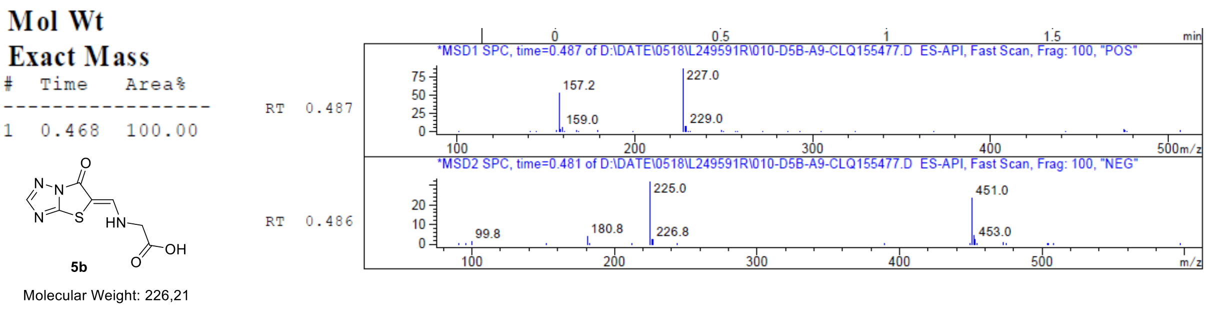

Figure S22. LC-MS spectrum **5b**

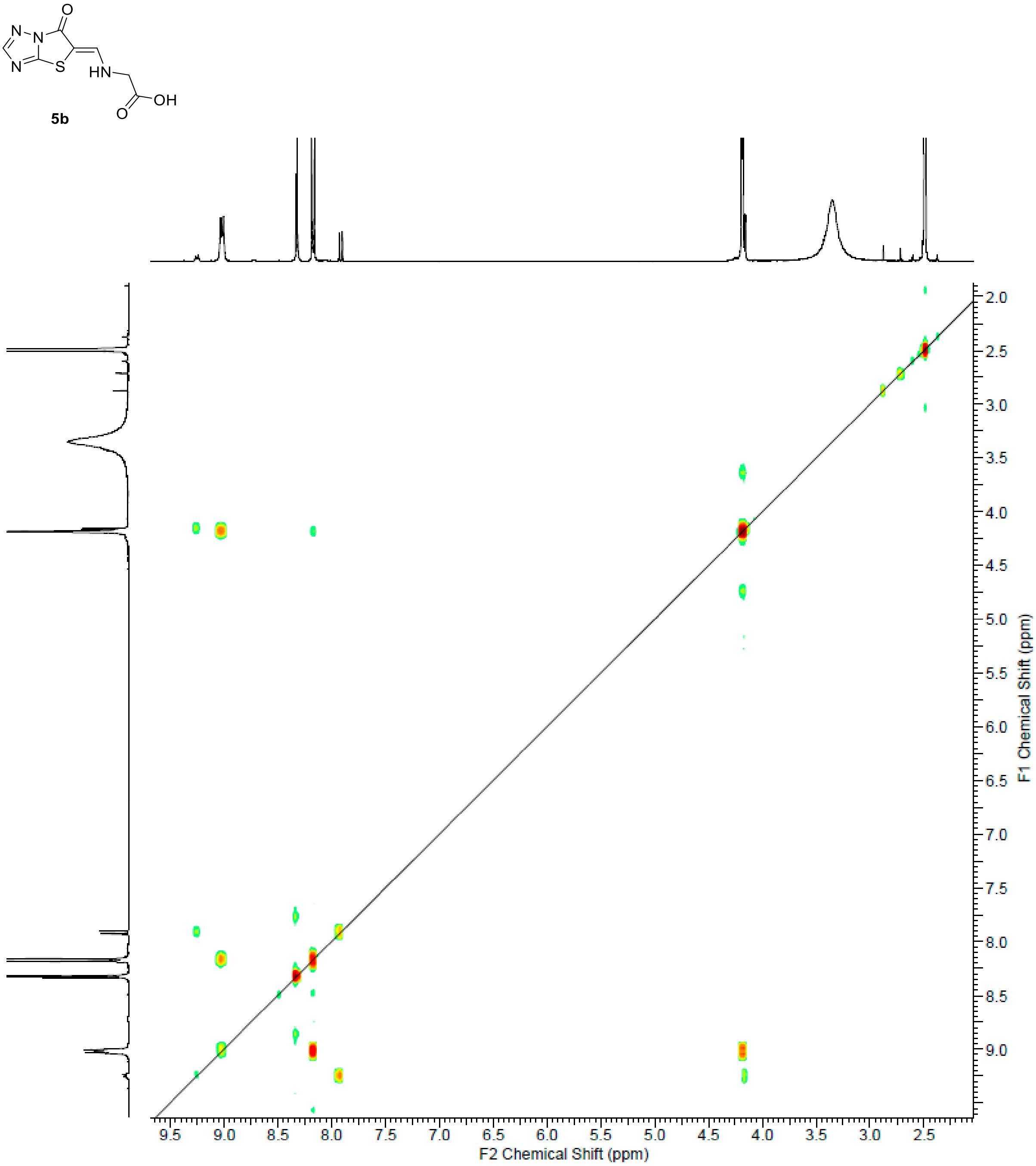

Figure S23. COSY spectrum **5b**

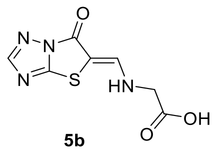

5b

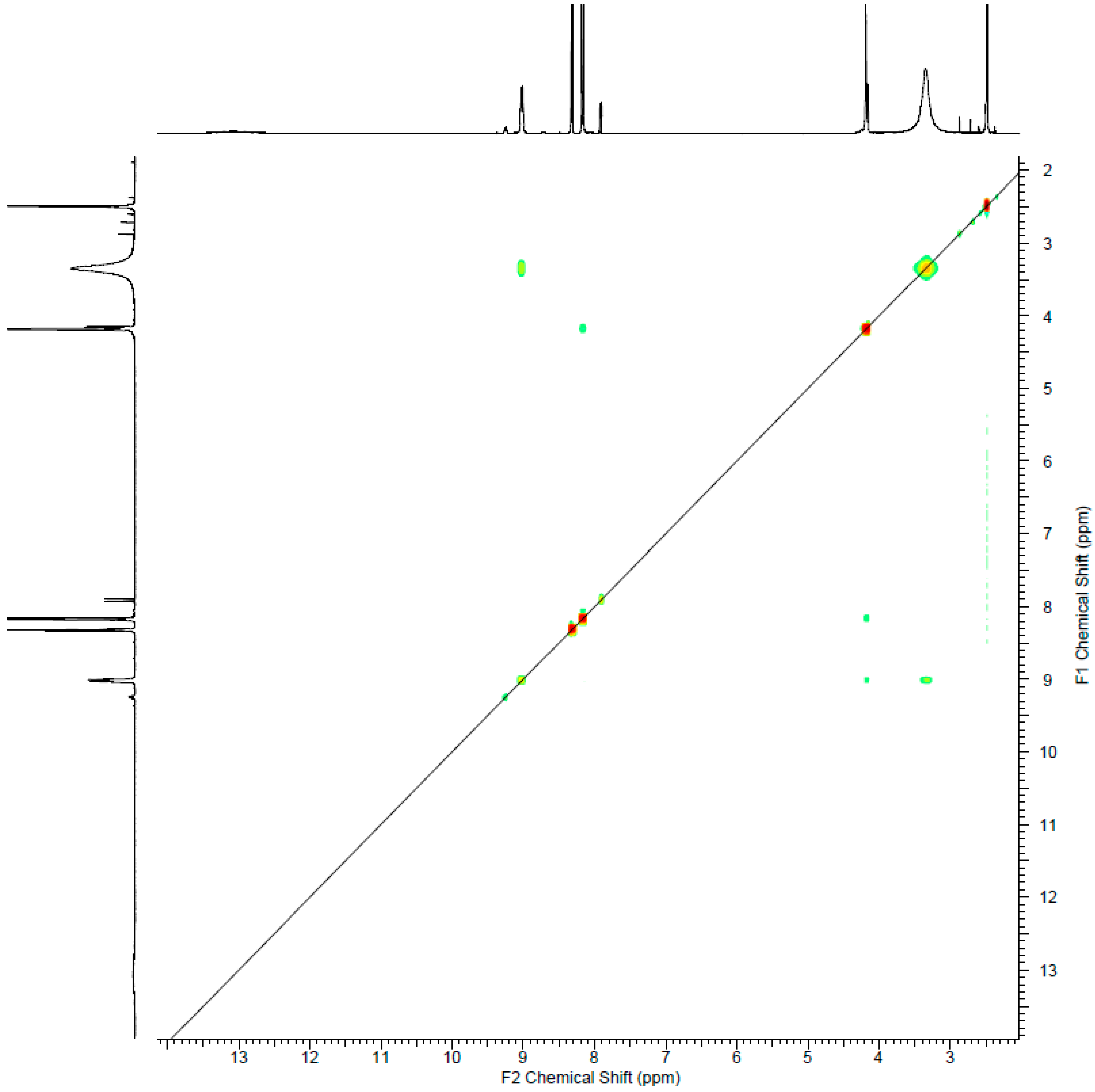

Figure S24. NOESY spectrum **5b**

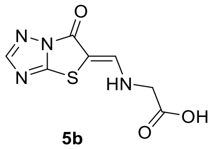

5b

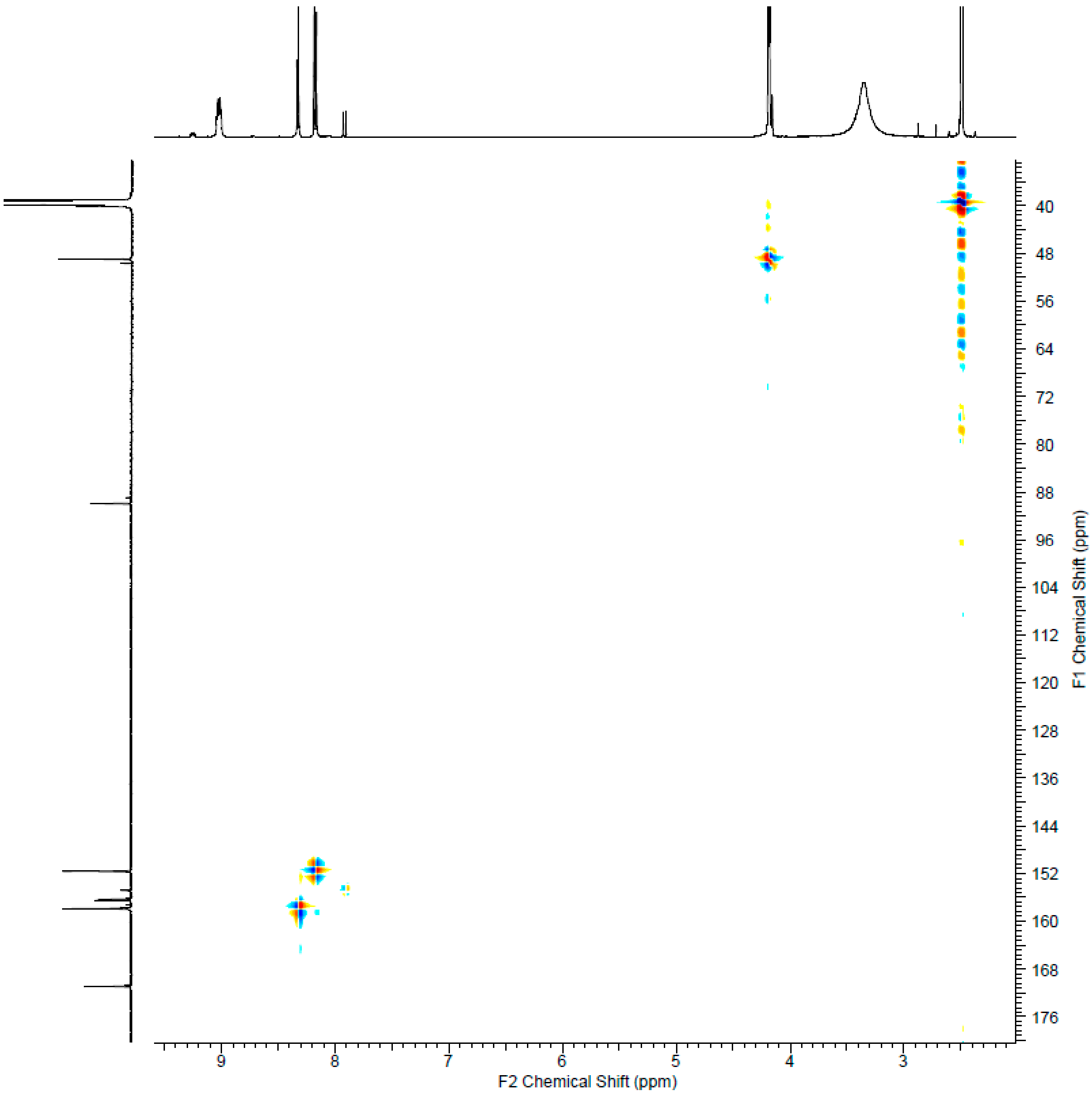

Figure S25. HSQC spectrum **5b**

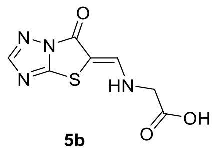

5b

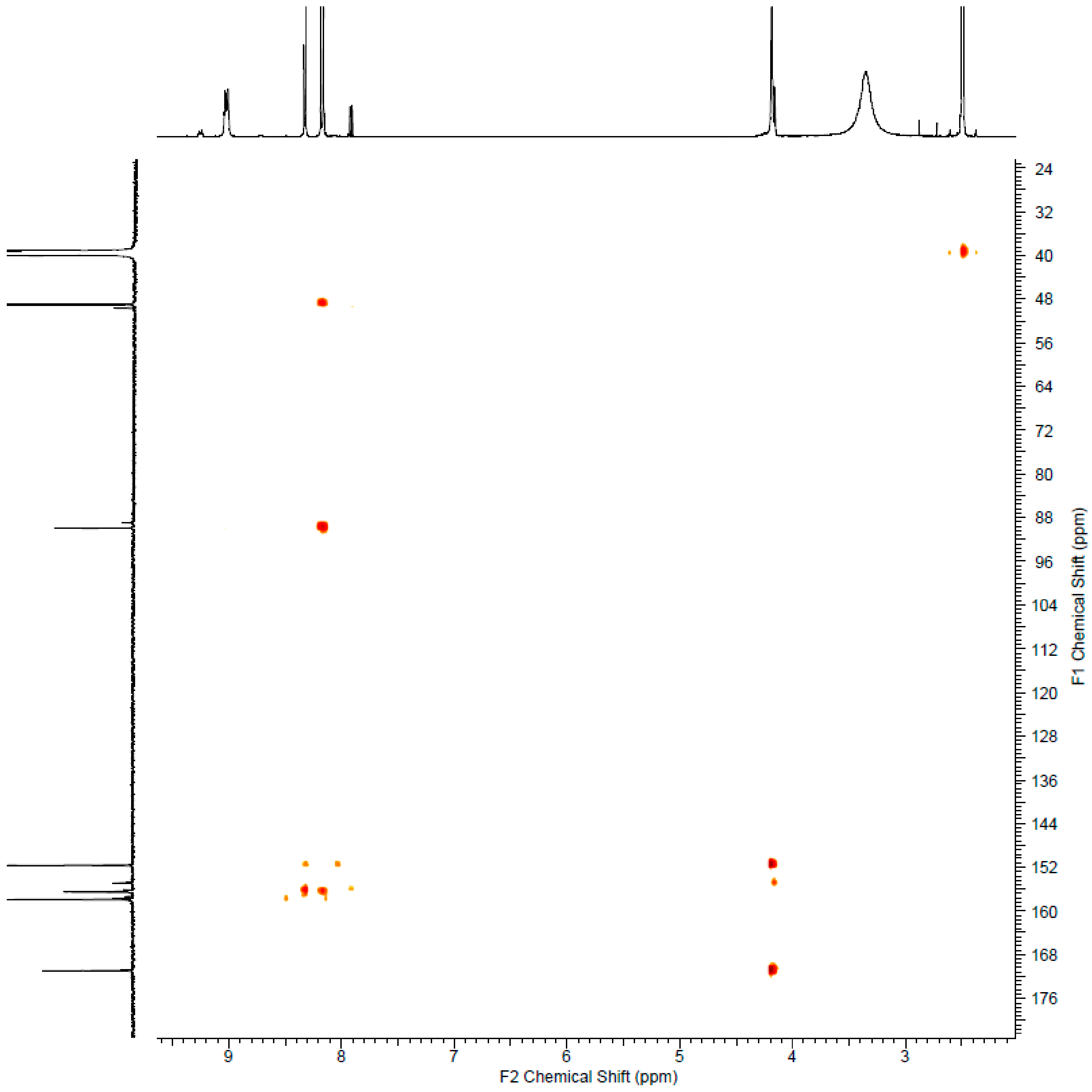

Figure S26. HMBC spectrum **5b**

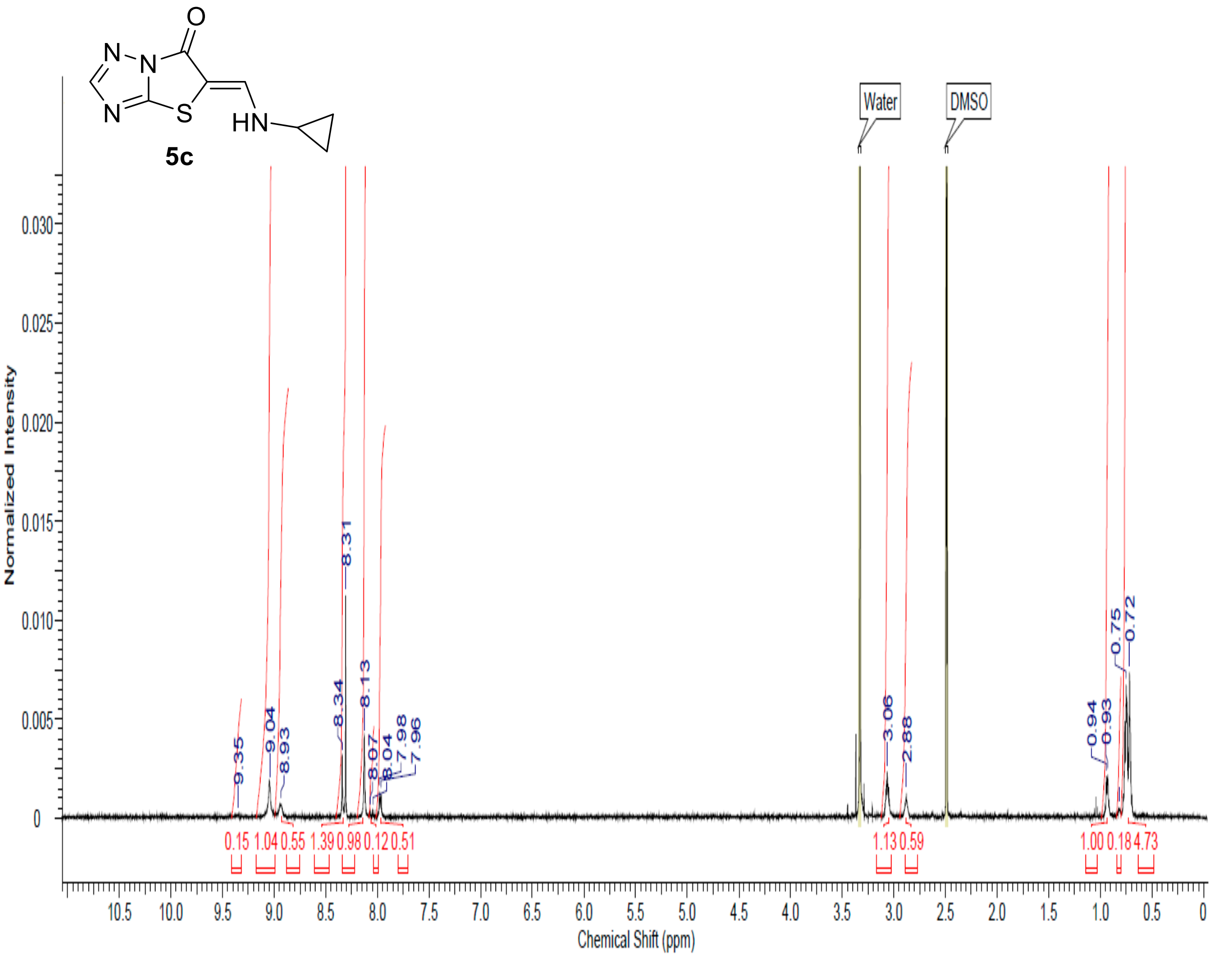

Figure S27. ^{1}H NMR spectrum **5c**

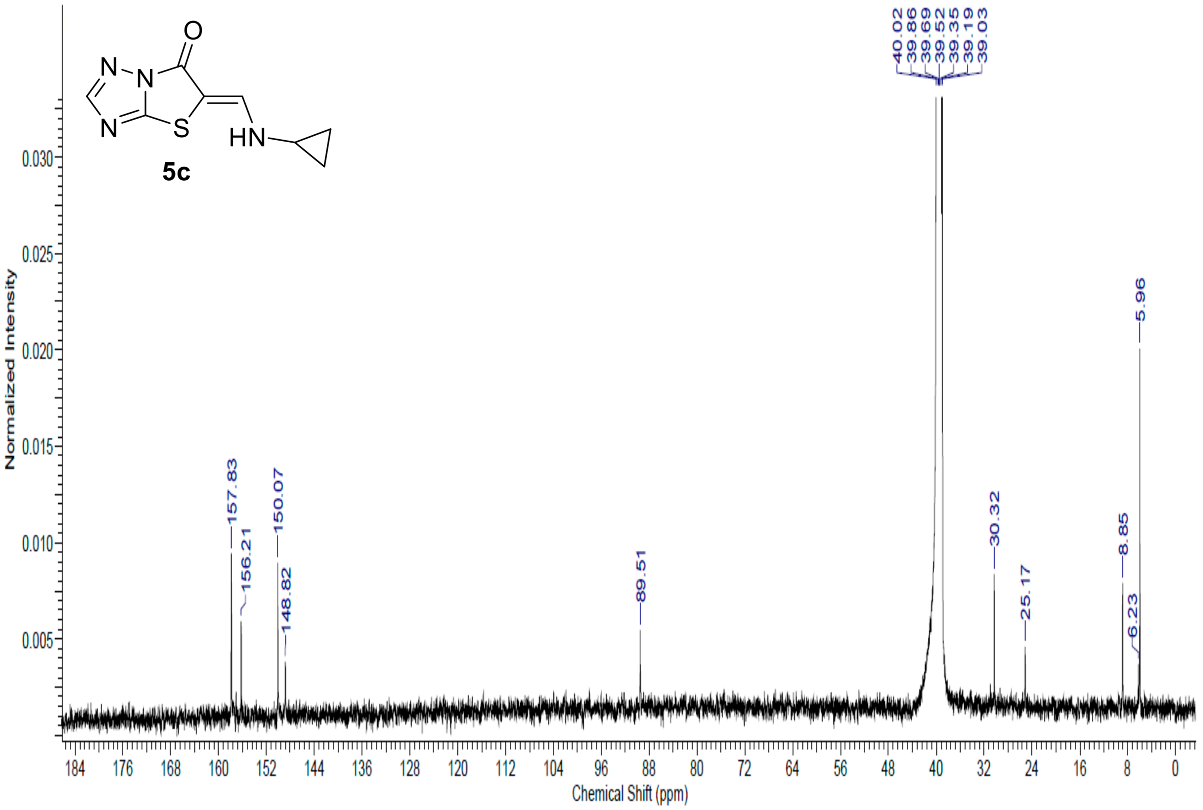

Figure S28. ^{13}C NMR spectrum **5c**

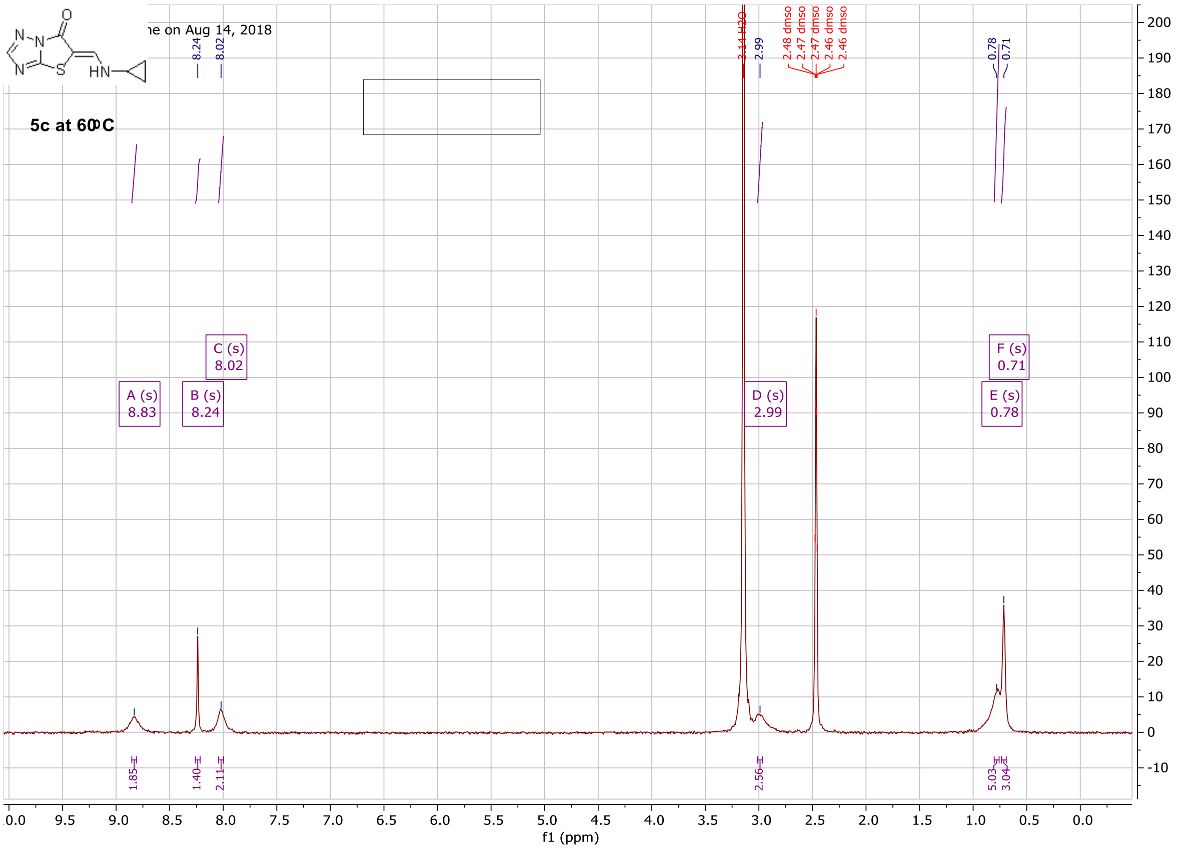

Figure S29. ^{1}H NMR spectrum **5c** at 60°C

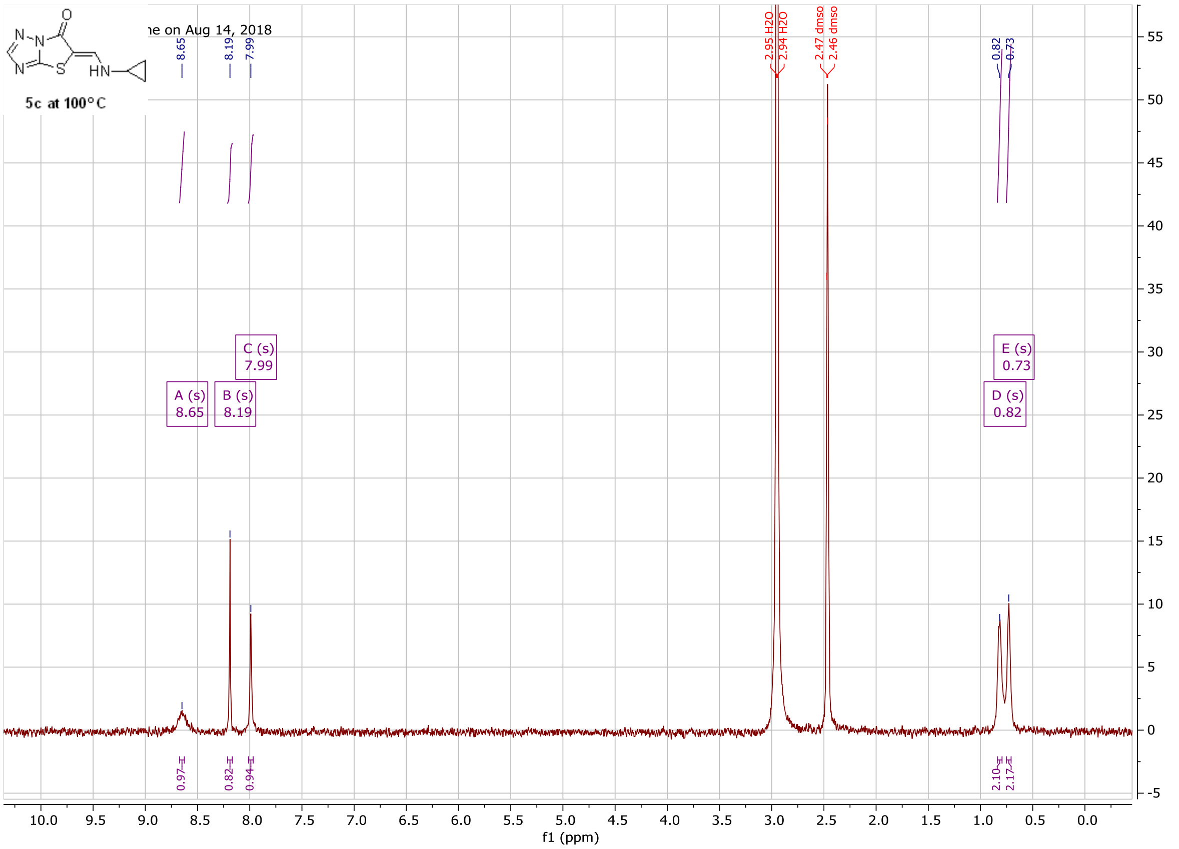

Figure S30. ^{1}H NMR spectrum **5c** at 100°C

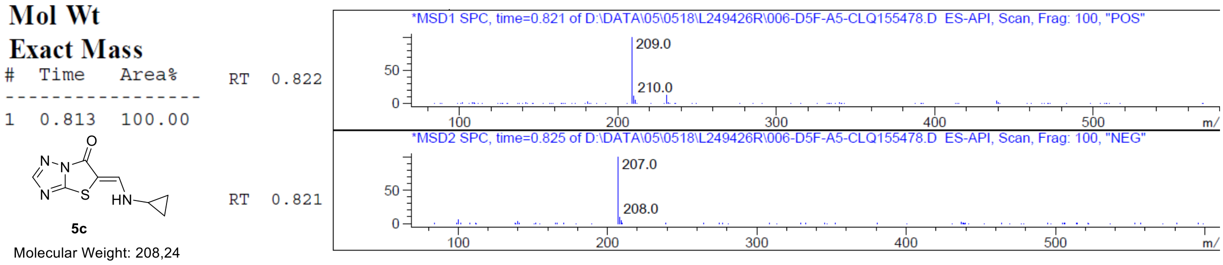

Figure S31. LC-MS spectrum **5c**

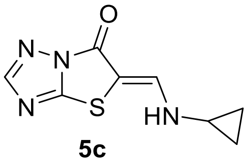

5c

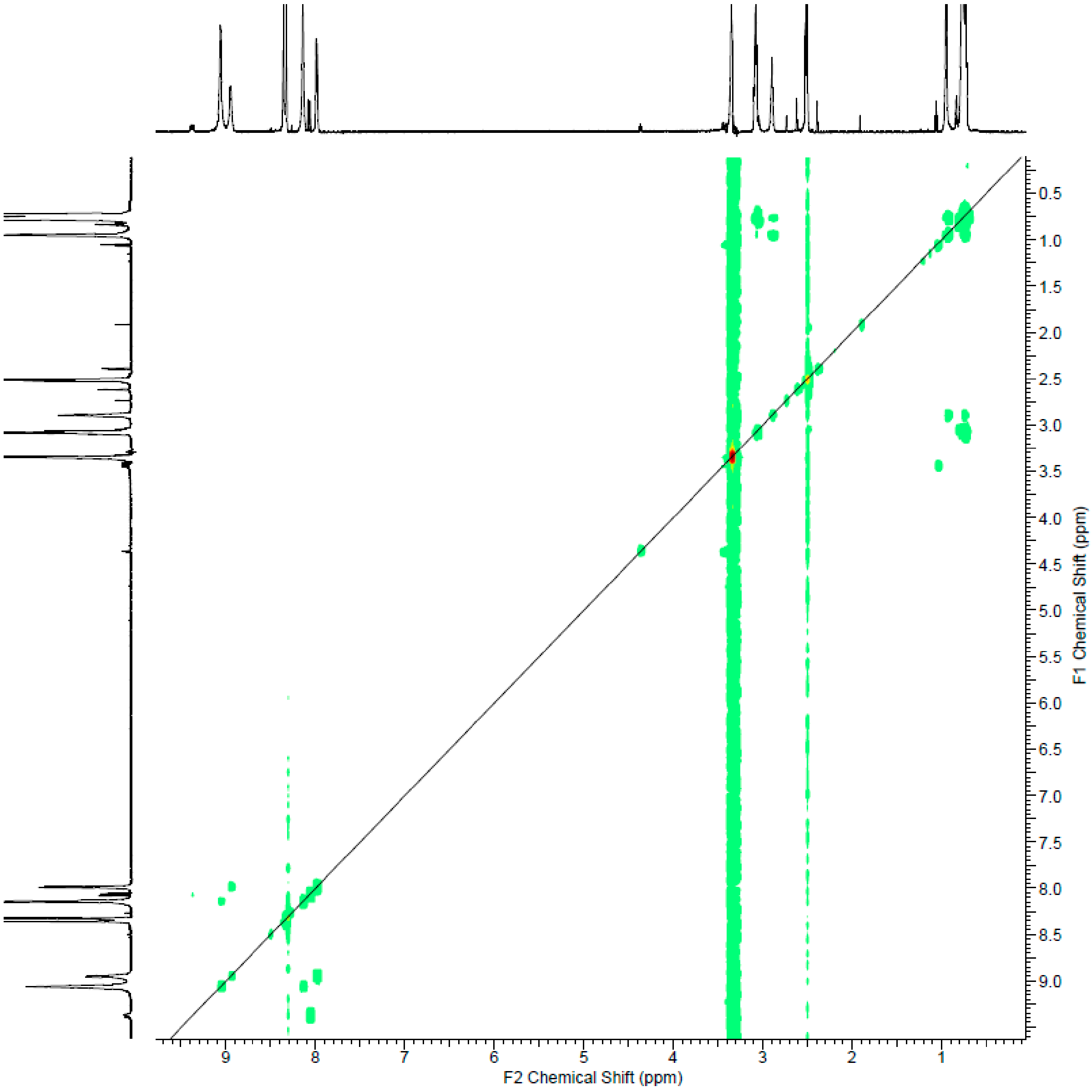

Figure S32. COSY spectrum **5c**

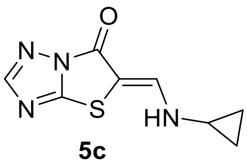

5c

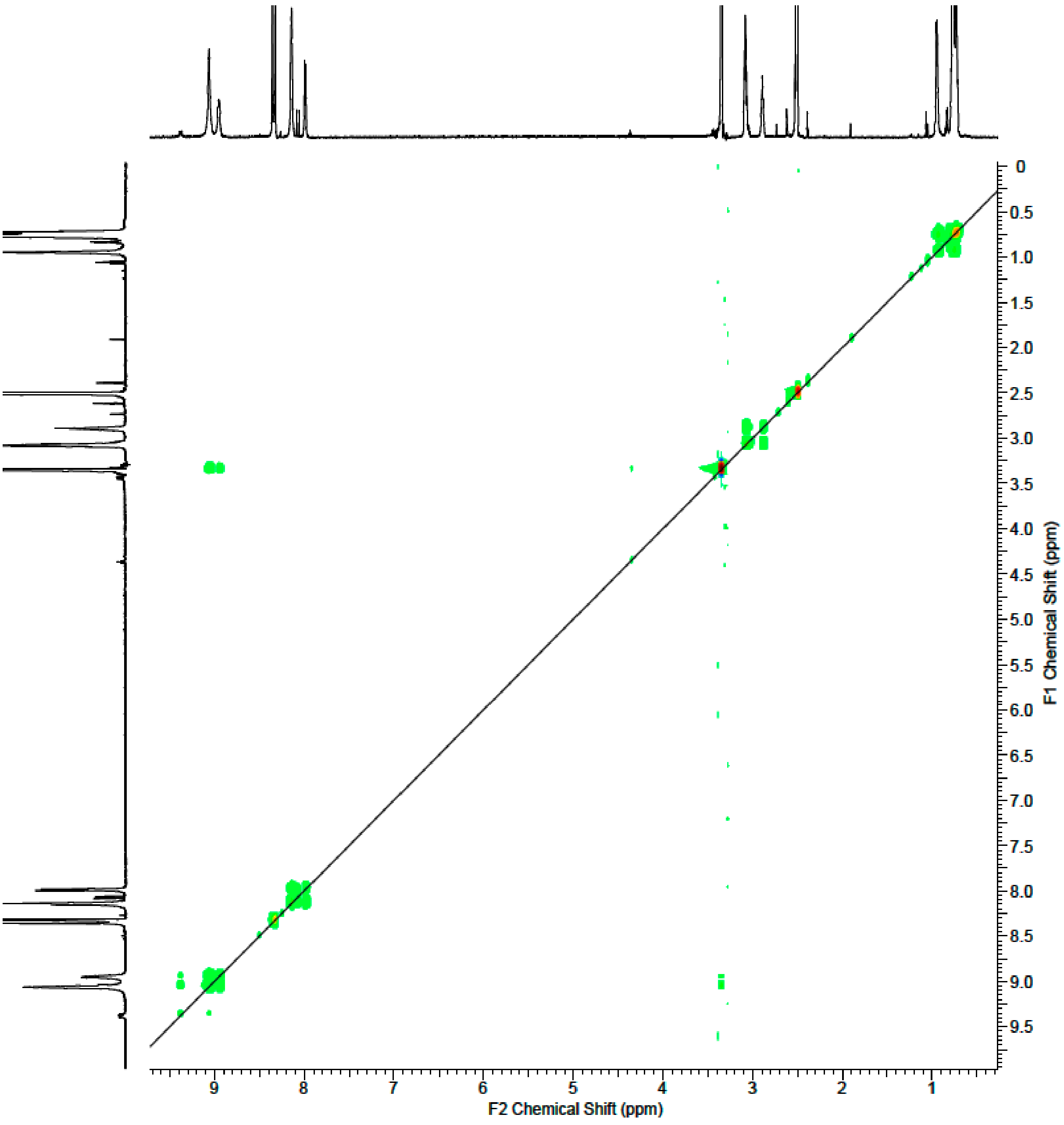

Figure S33. NOESY spectrum **5c**

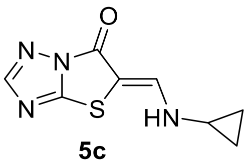

5c

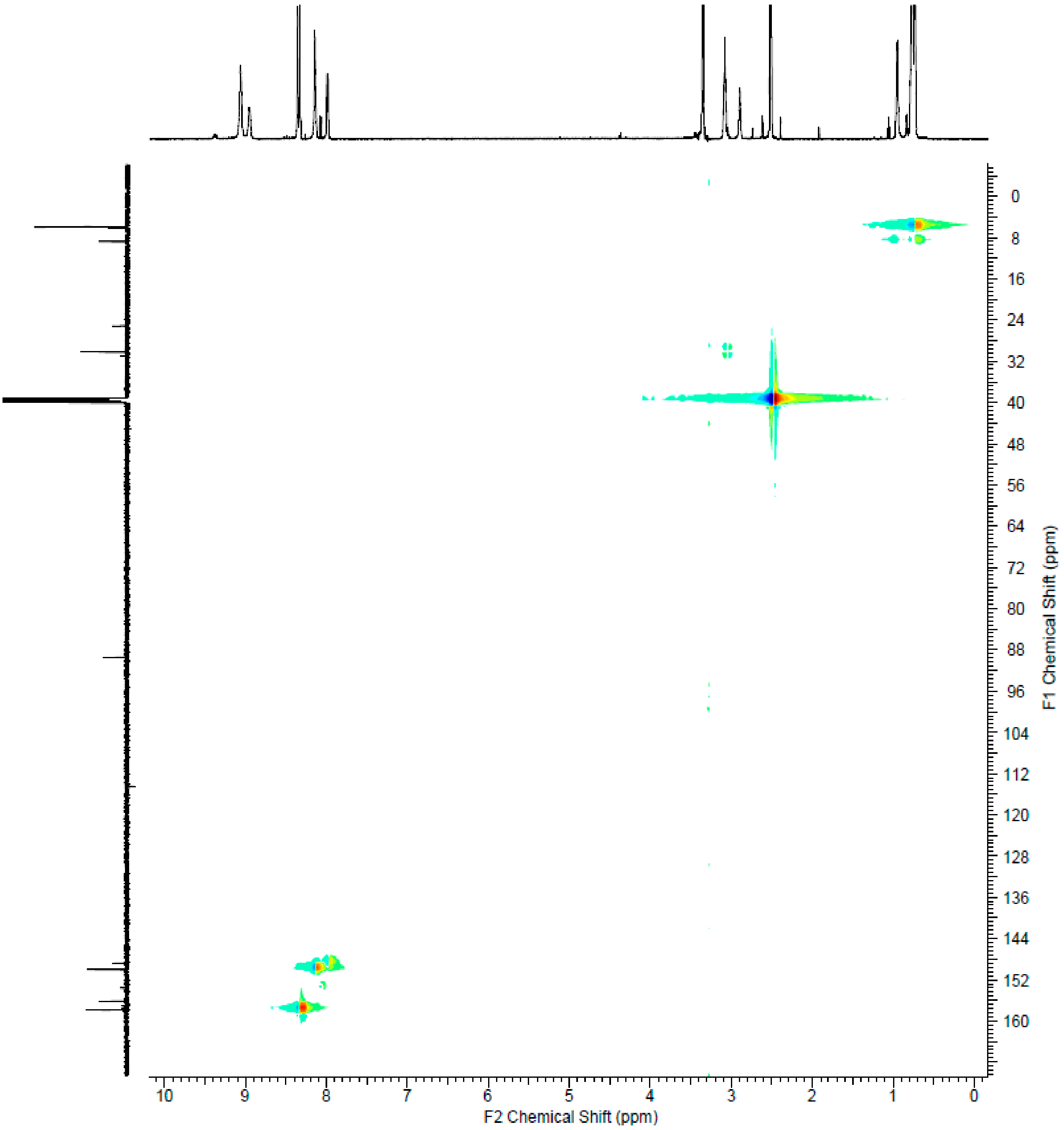

Figure S34. HSQC spectrum **5c**

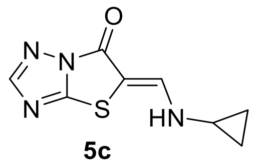

5c

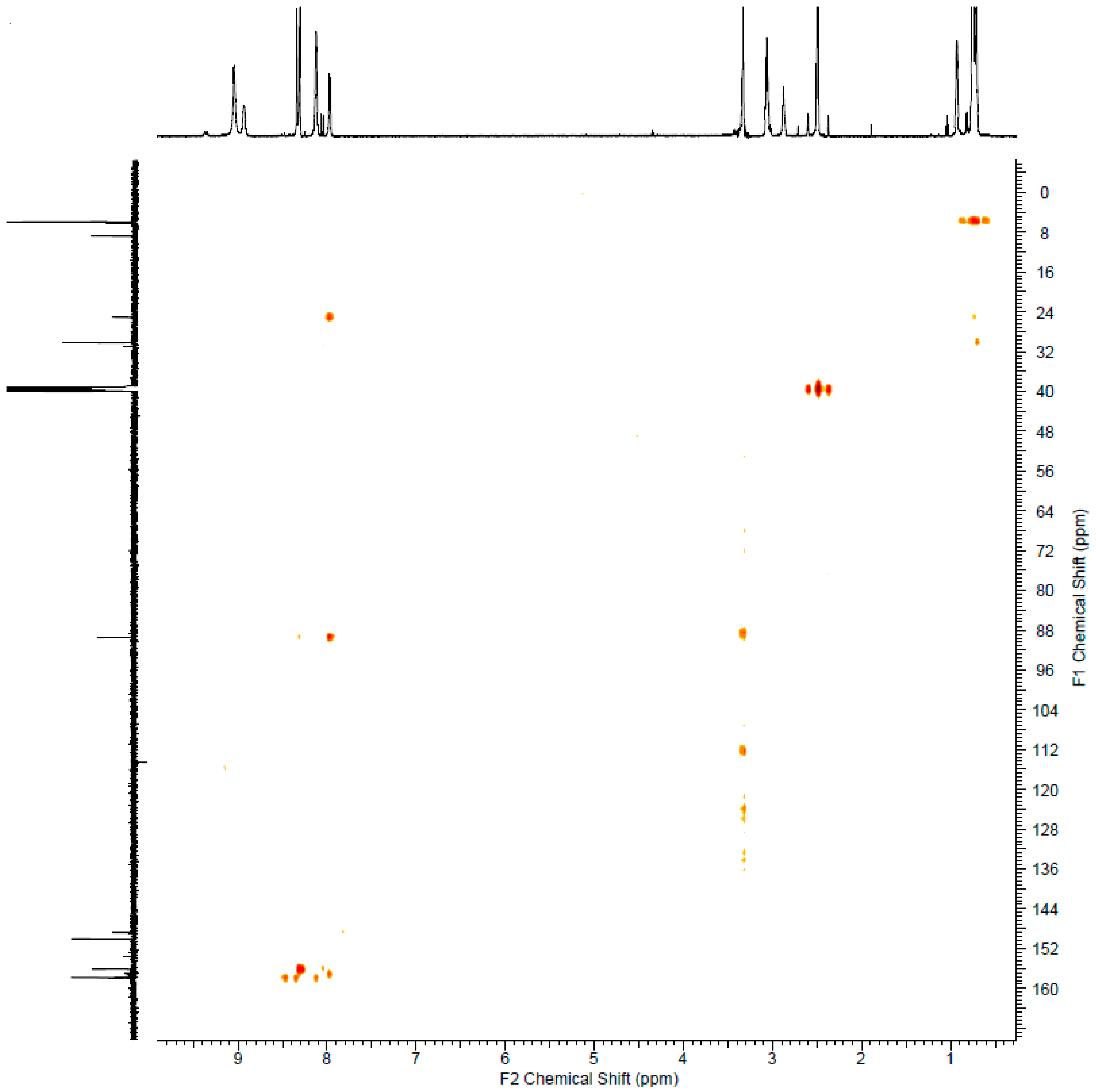

Figure S35. HMBC spectrum **5c**

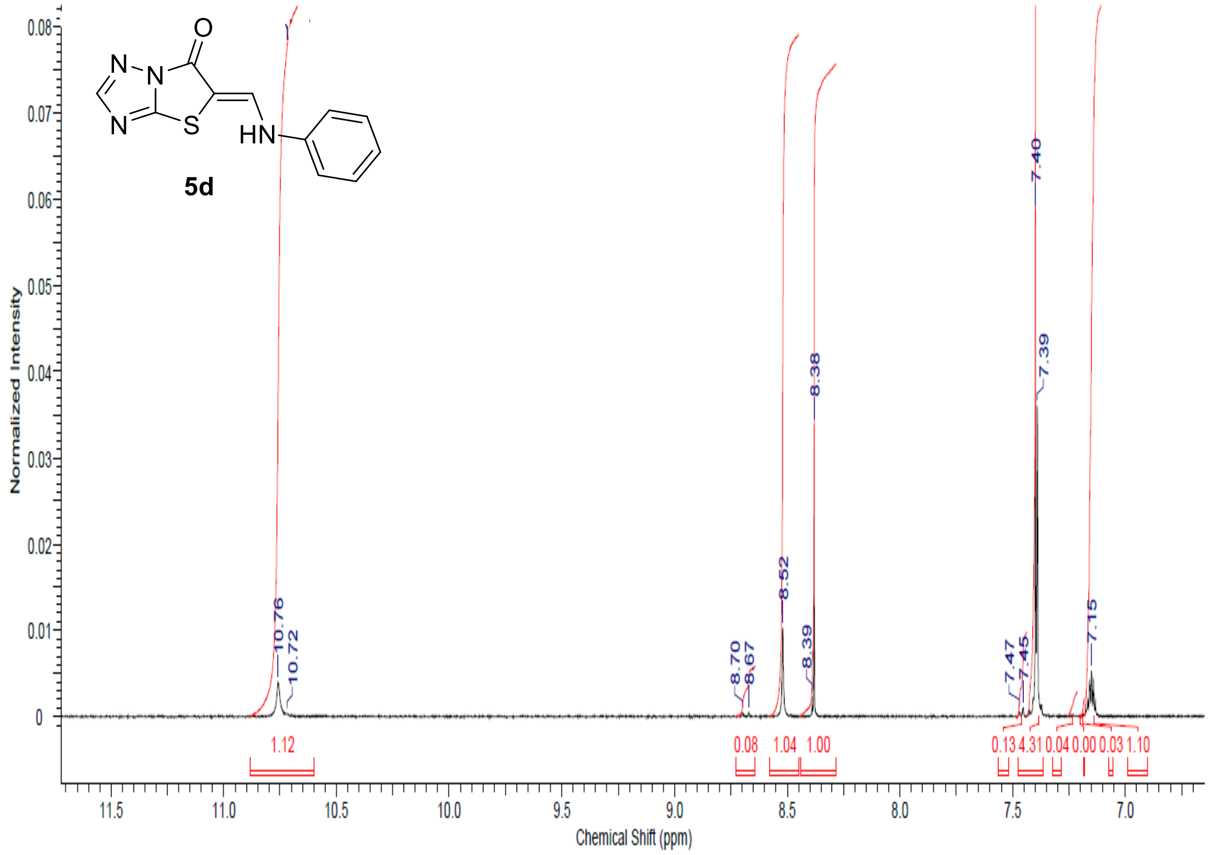

Figure S36. ^{1}H NMR spectrum **5d**

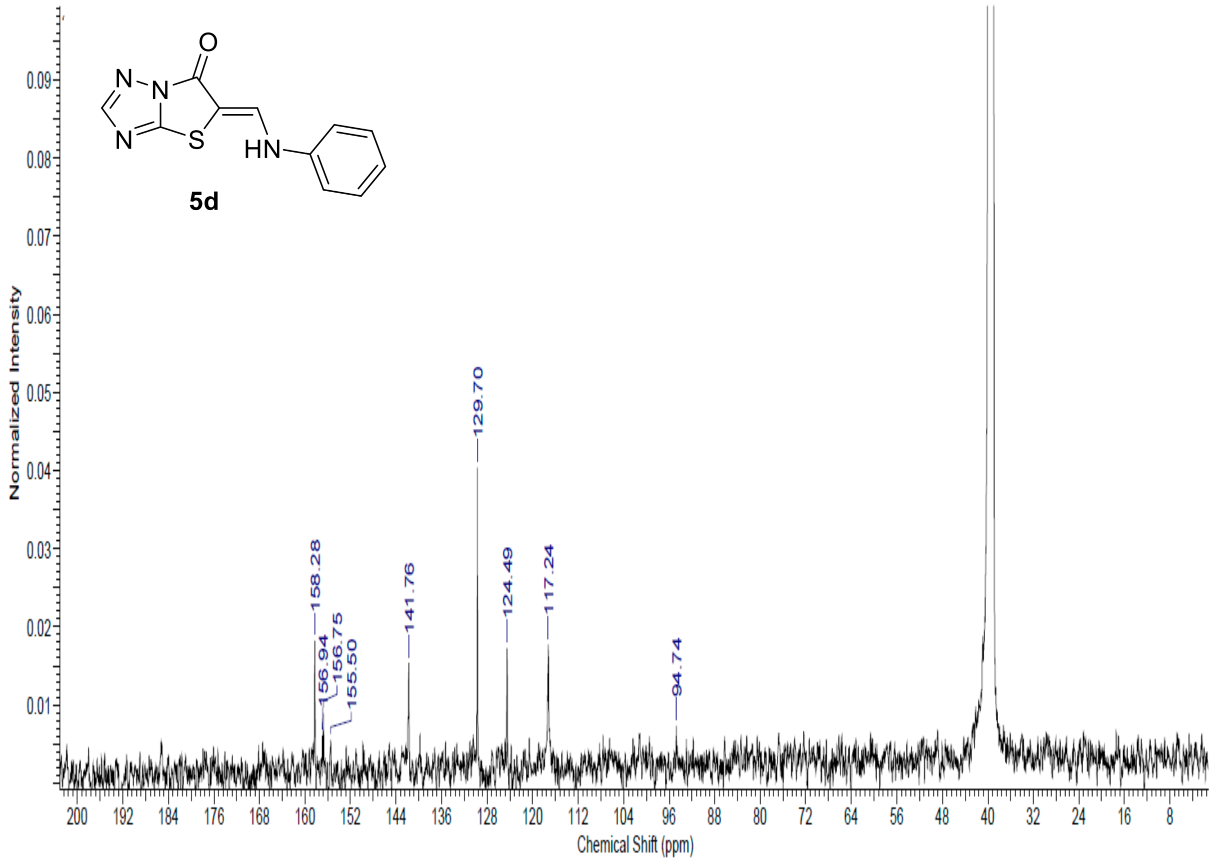

Figure S37. ^{13}C NMR spectrum **5d**

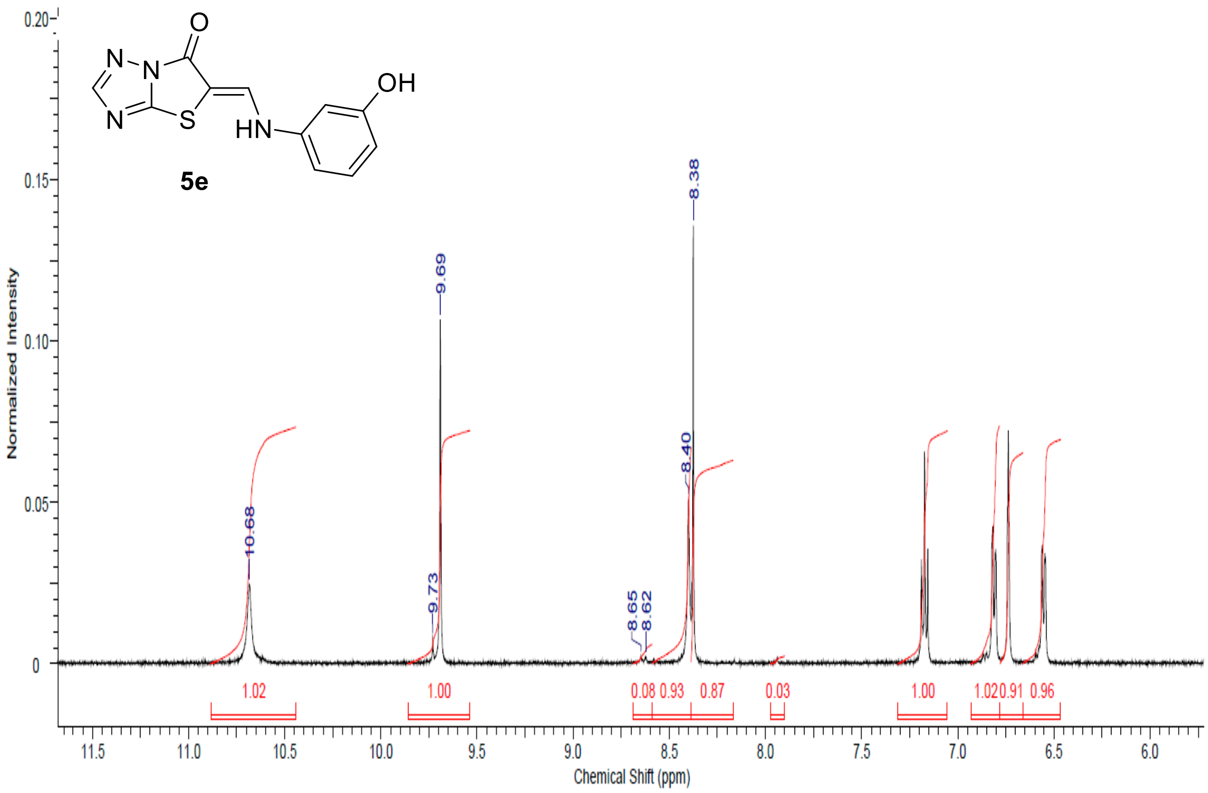

Figure S38. ^{1}H NMR spectrum **5e**

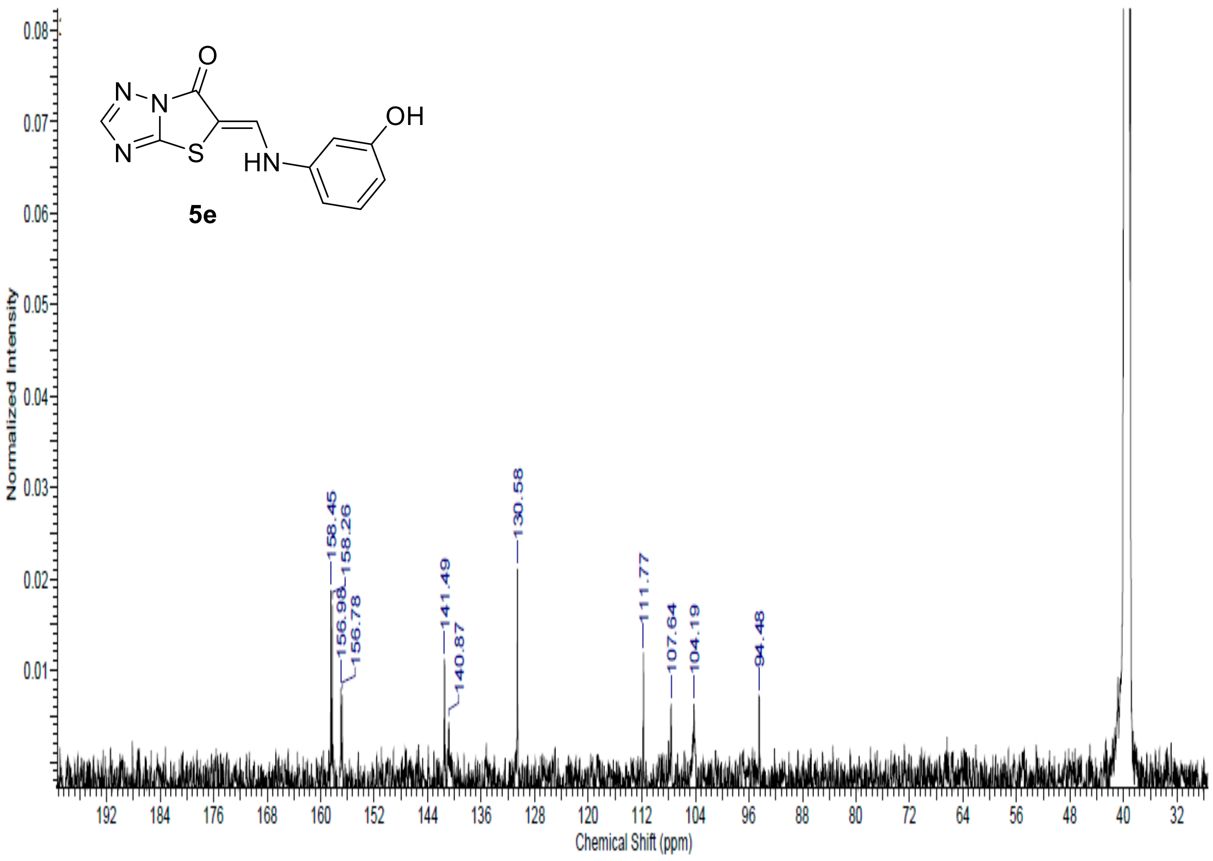

Figure S39. ^{13}C NMR spectrum **5e**

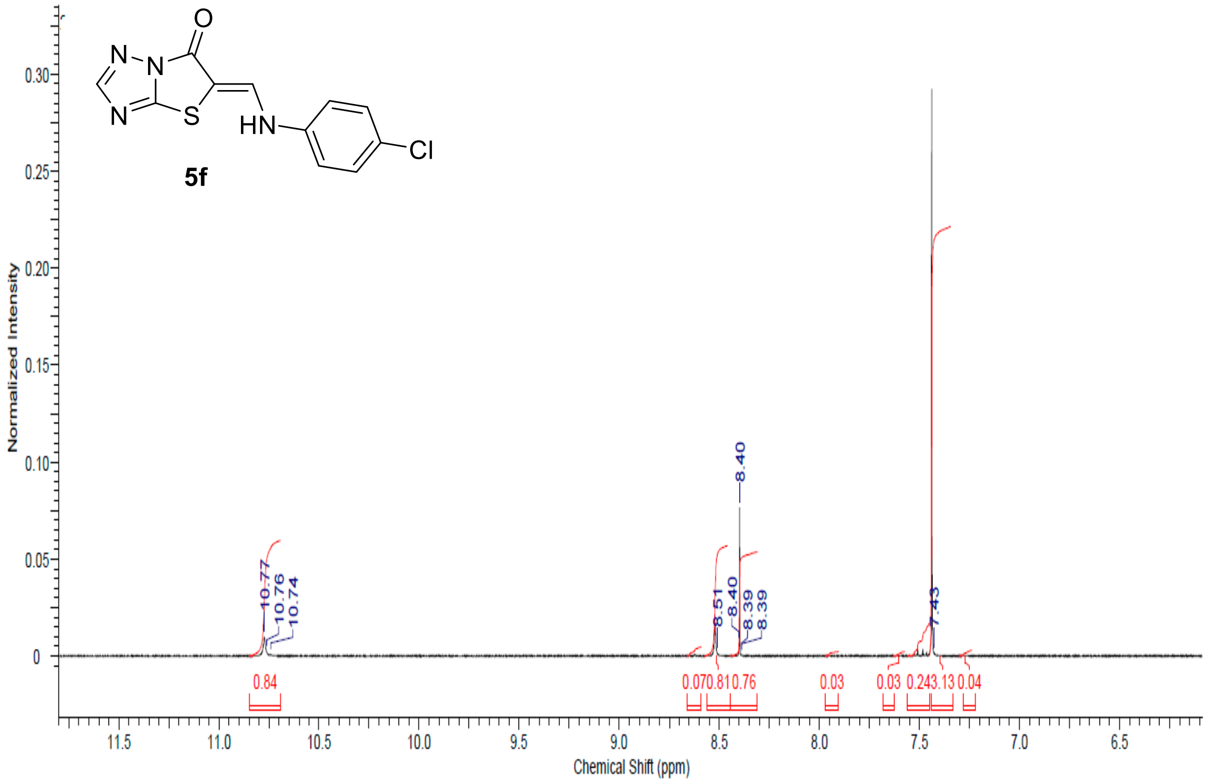

Figure S40. ^{1}H NMR spectrum **5f**

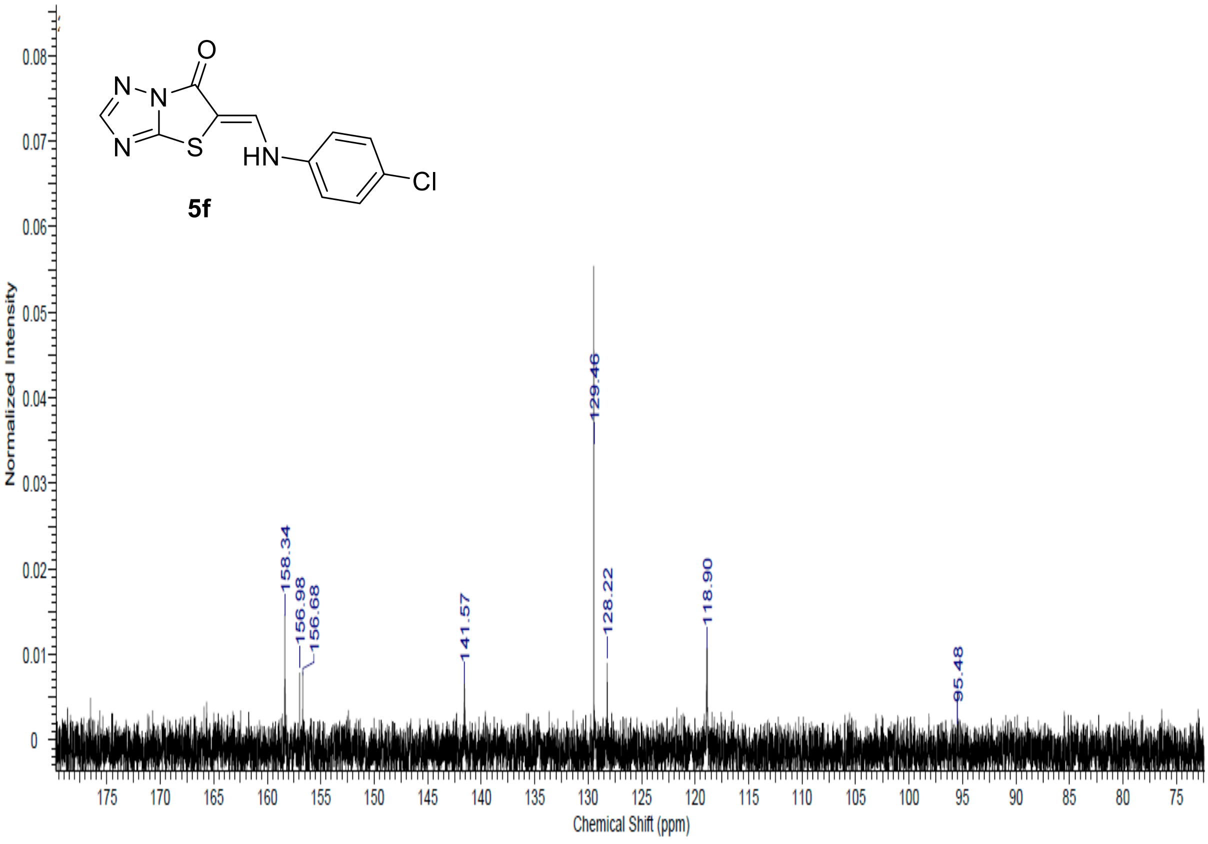

Figure S41. ^{13}C NMR spectrum **5f**

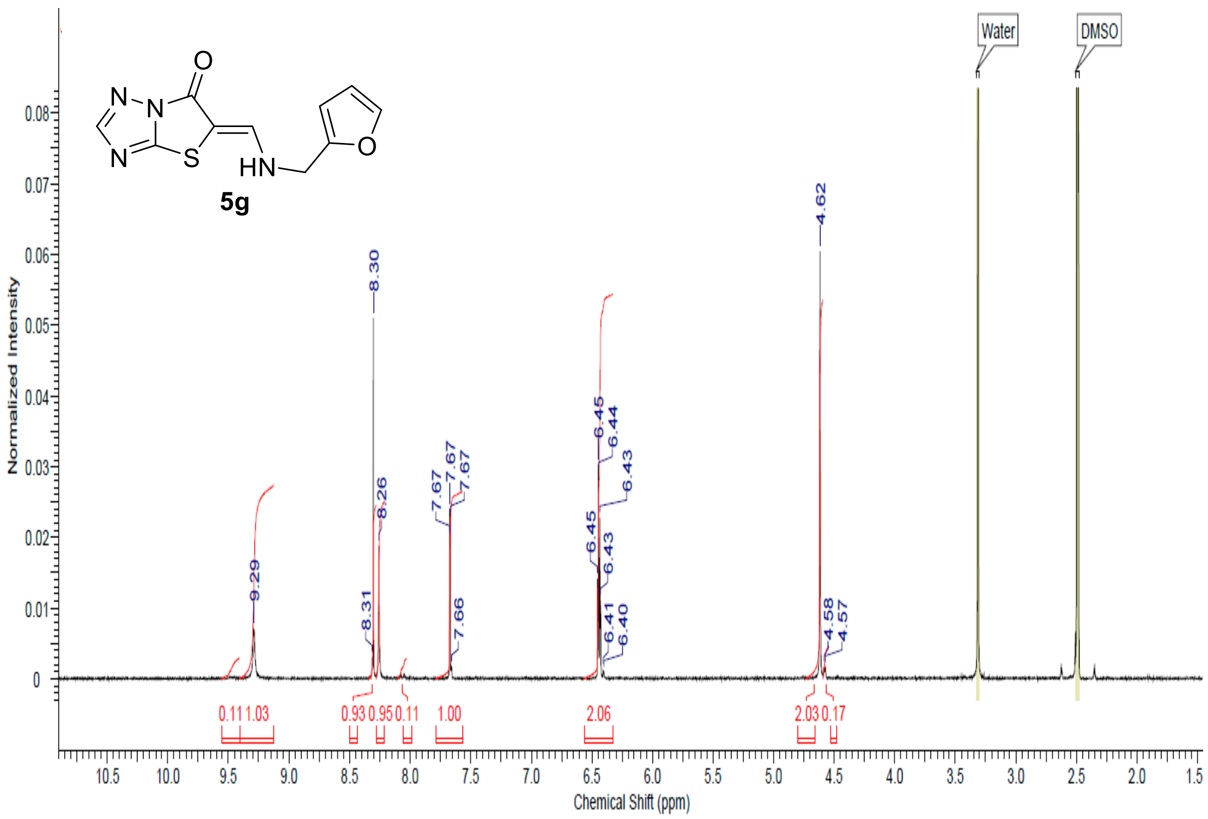

Figure S42. ^{1}H NMR spectrum **5g**

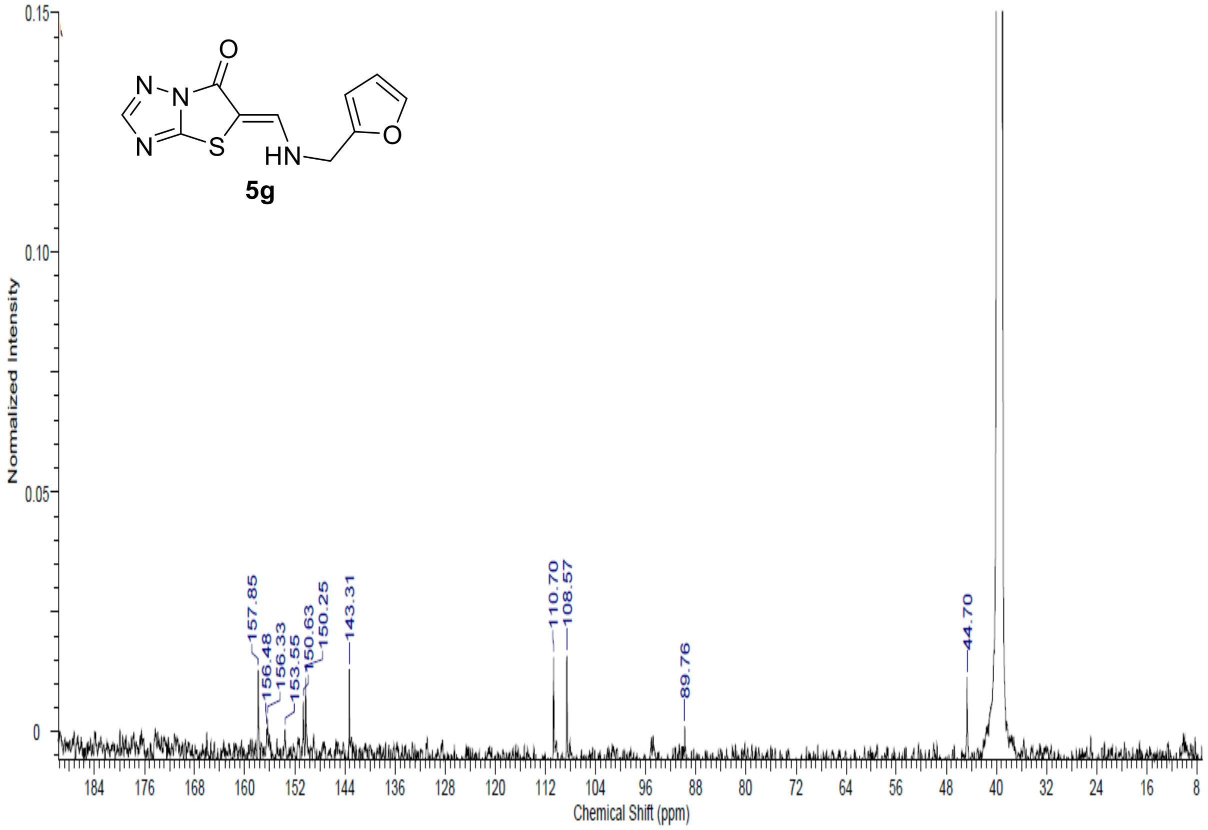

Figure S43. ^{13}C NMR spectrum **5g**

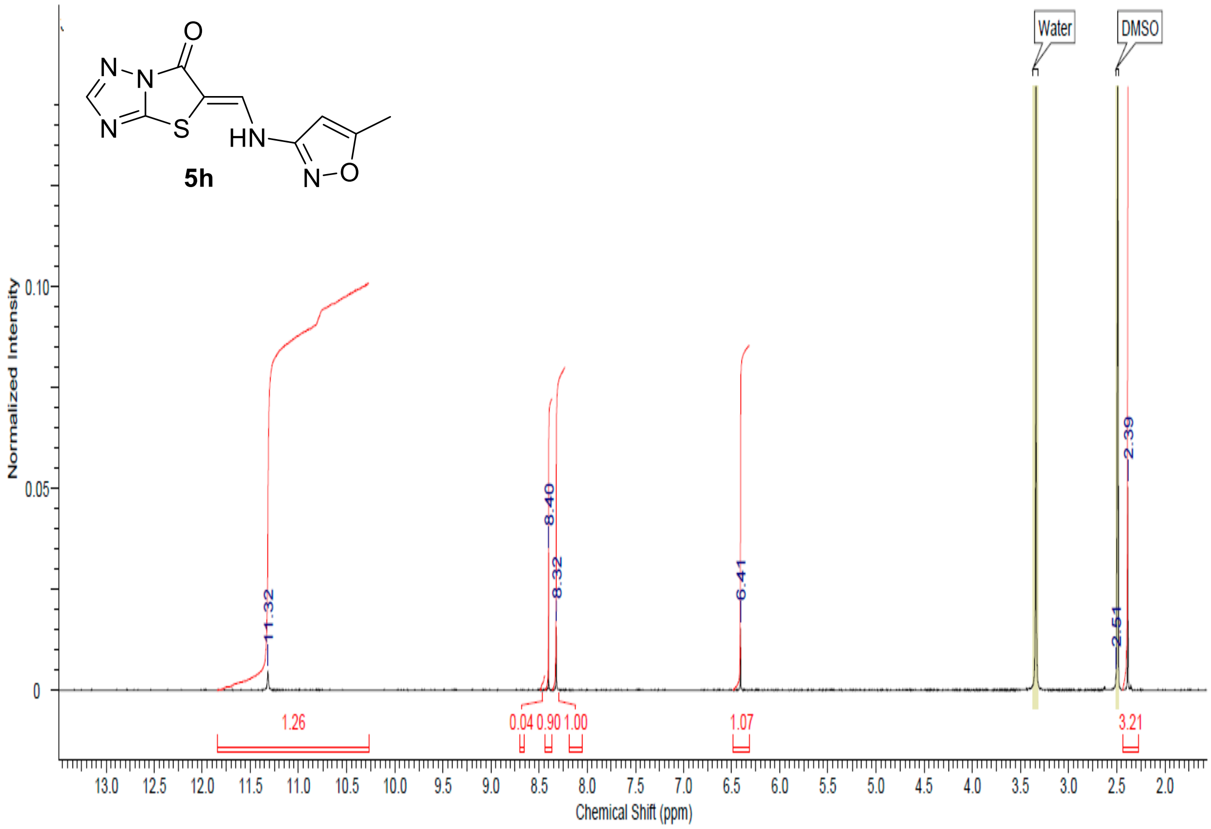

Figure S44. ^{1}H NMR spectrum **5h**

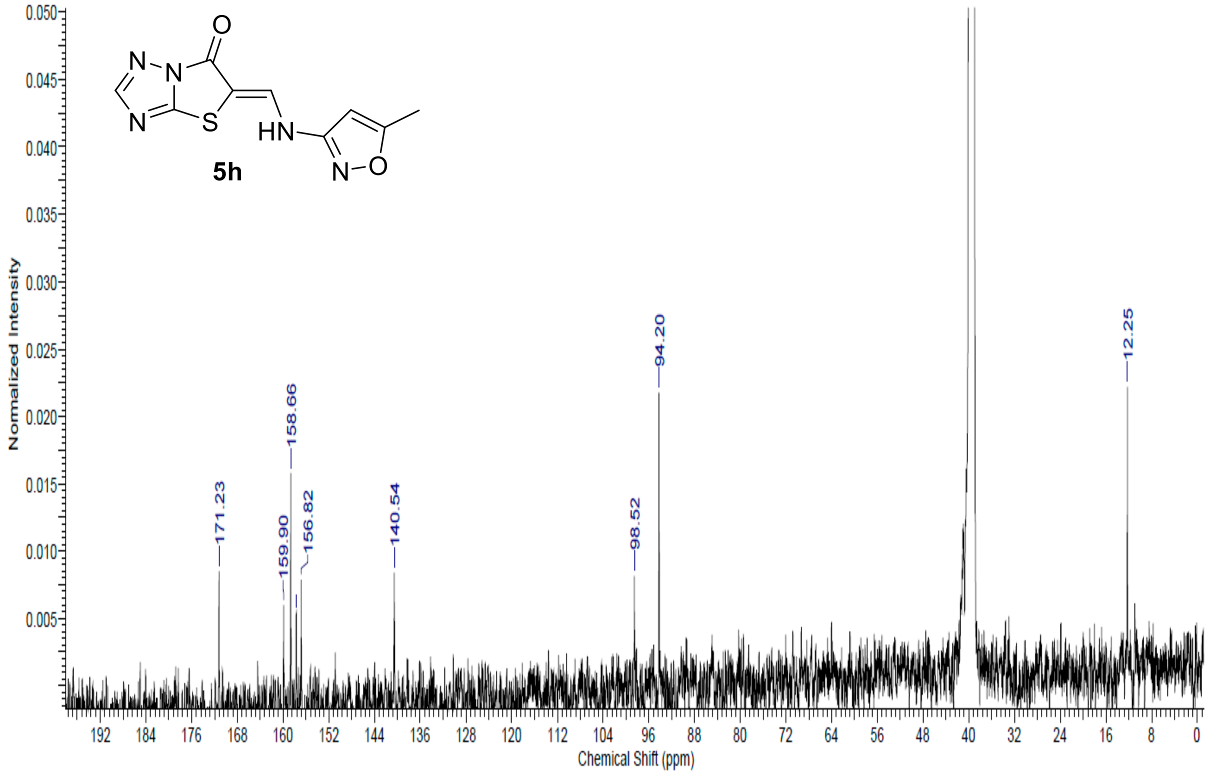

Figure S45. ^{13}C NMR spectrum **5h**

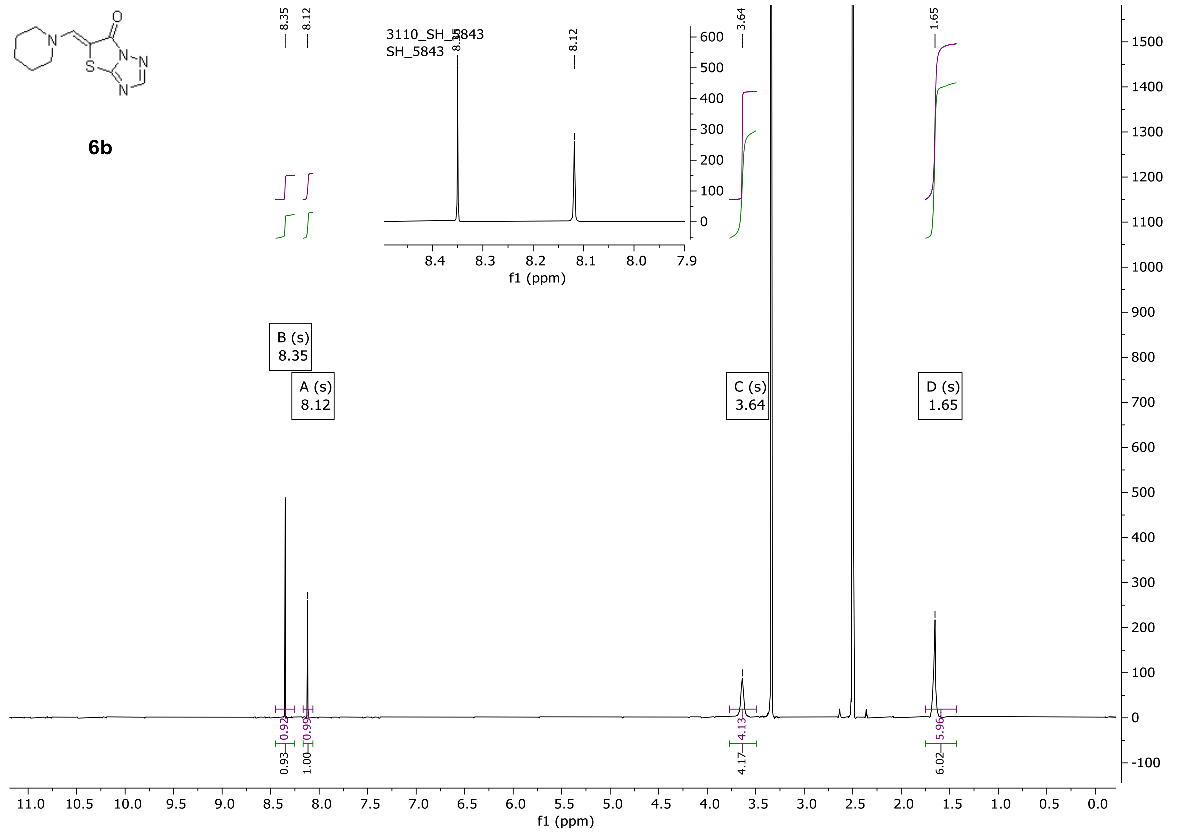

Figure S46. ^{1}H NMR spectrum **6b**

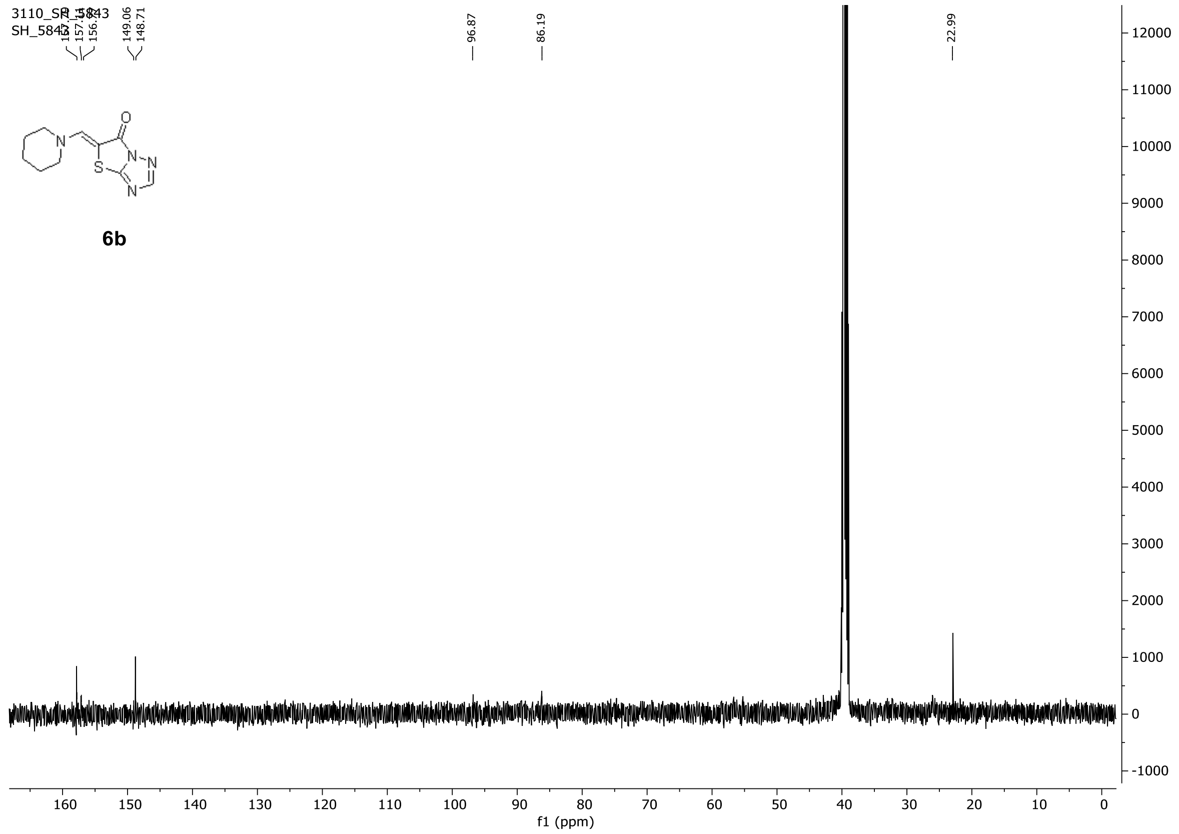

Figure S47. ^{13}C NMR spectrum **6b**

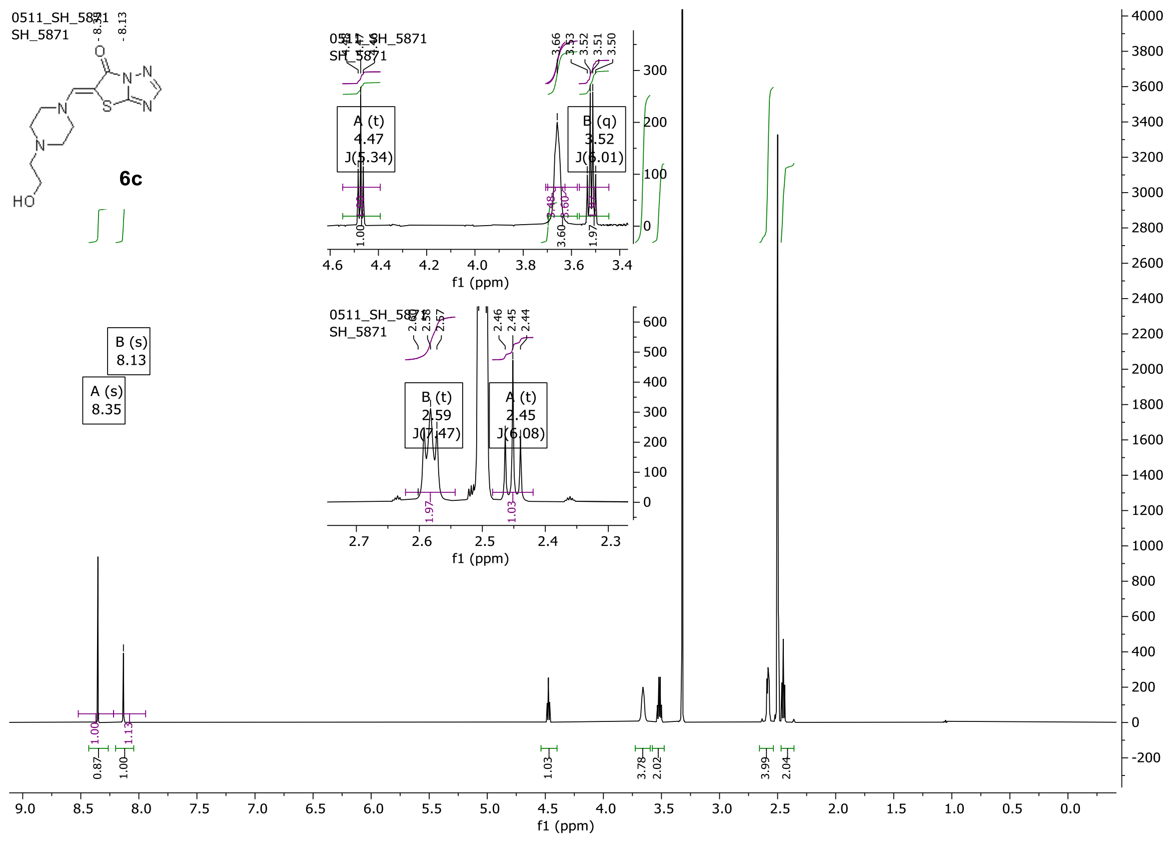

Figure S48. ^{1}H NMR spectrum **6c**

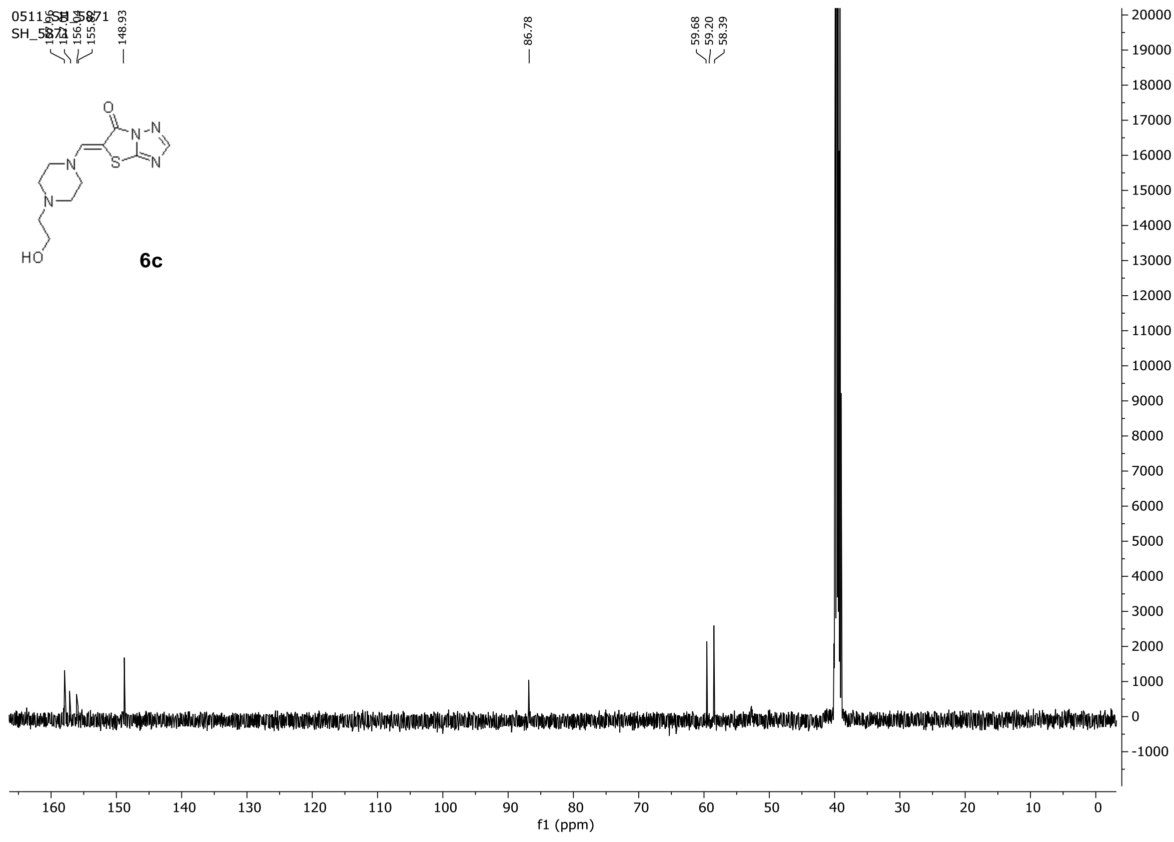

Figure S49. ^{13}C NMR spectrum **6c**
